# Supplementary material for: Single-Domain Antibodies as Crystallization Chaperones to Enable Structure-Based Inhibitor Development for RBR E3 Ubiquitin Ligases
Source: Cell Chem Biol. 2020 Jan 16;27(1):83–93.e9. doi: 10.1016/j.chembiol.2019.11.007 (PMC6963773; doi:10.1016/j.chembiol.2019.11.007)
Supplement: Document S1. Figures S1–S6 and Tables S1–S3 [file mmc1.pdf]

Cell Chemical Biology, Volume *27*

Supplemental Information

Single-Domain Antibodies as Crystallization Chaperones to Enable Structure-Based Inhibitor Development for RBR E3 Ubiquitin Ligases

Yi-Chun Isabella Tsai, Henrik Johansson, David Dixon, Stephen Martin, Chun-wa Chung, Jane Clarkson, David House, and Katrin Rittinger

Supplemental Information

SI Item List

Figure S1

Figure S2

Figure S3

Figure S4

Figure S5

Figure S6

Table S1

Table S2

Table S3

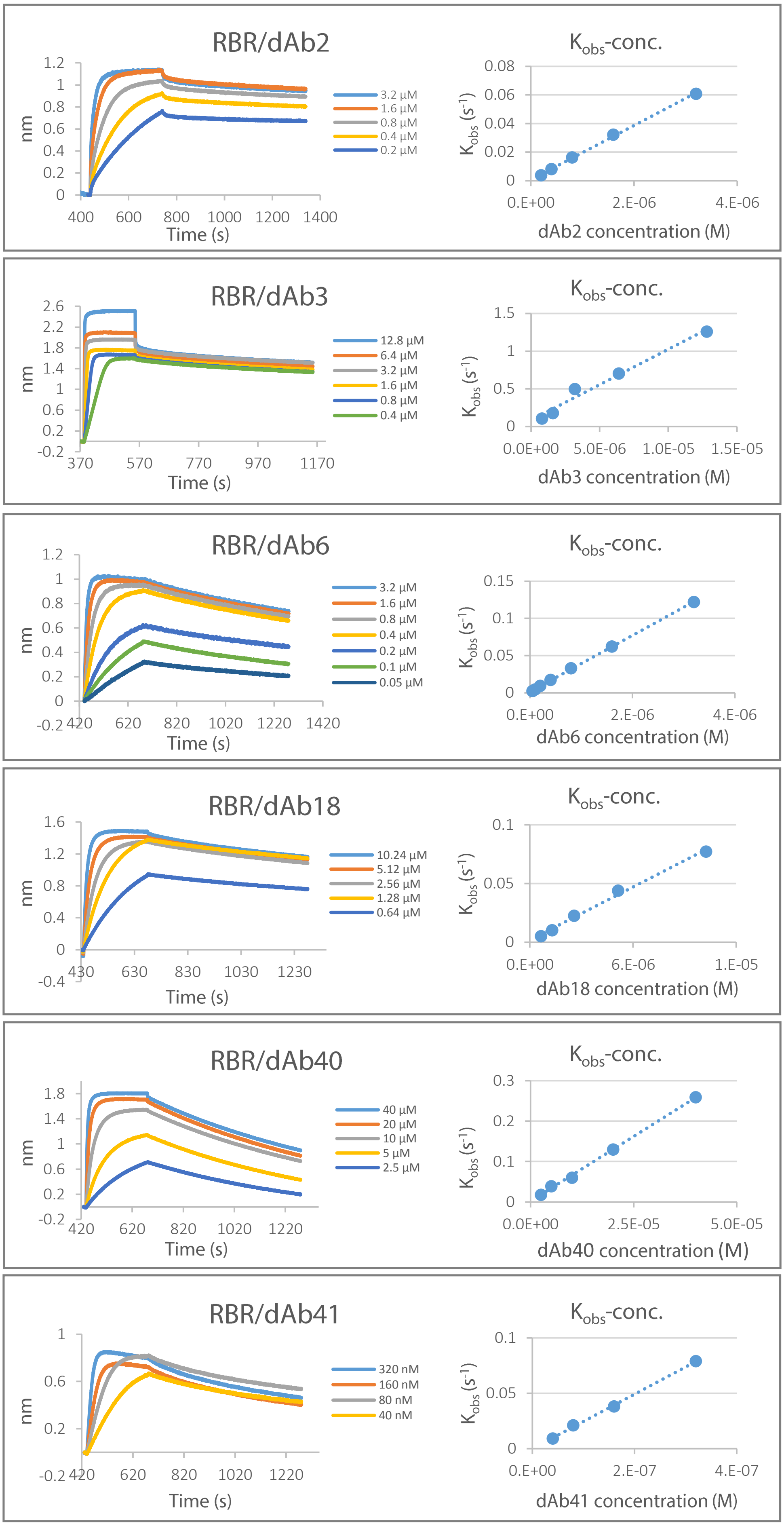

Figure S1. Related to Table 1. Kinetic analysis of the interaction between HOIP RBR and selected dAbs
Sensorgram traces displaying association and dissociation steps. Association rate constants (k_{on}) were obtained from the slope of the observed rate (k_{obs}) plotted versus dAb concentration.

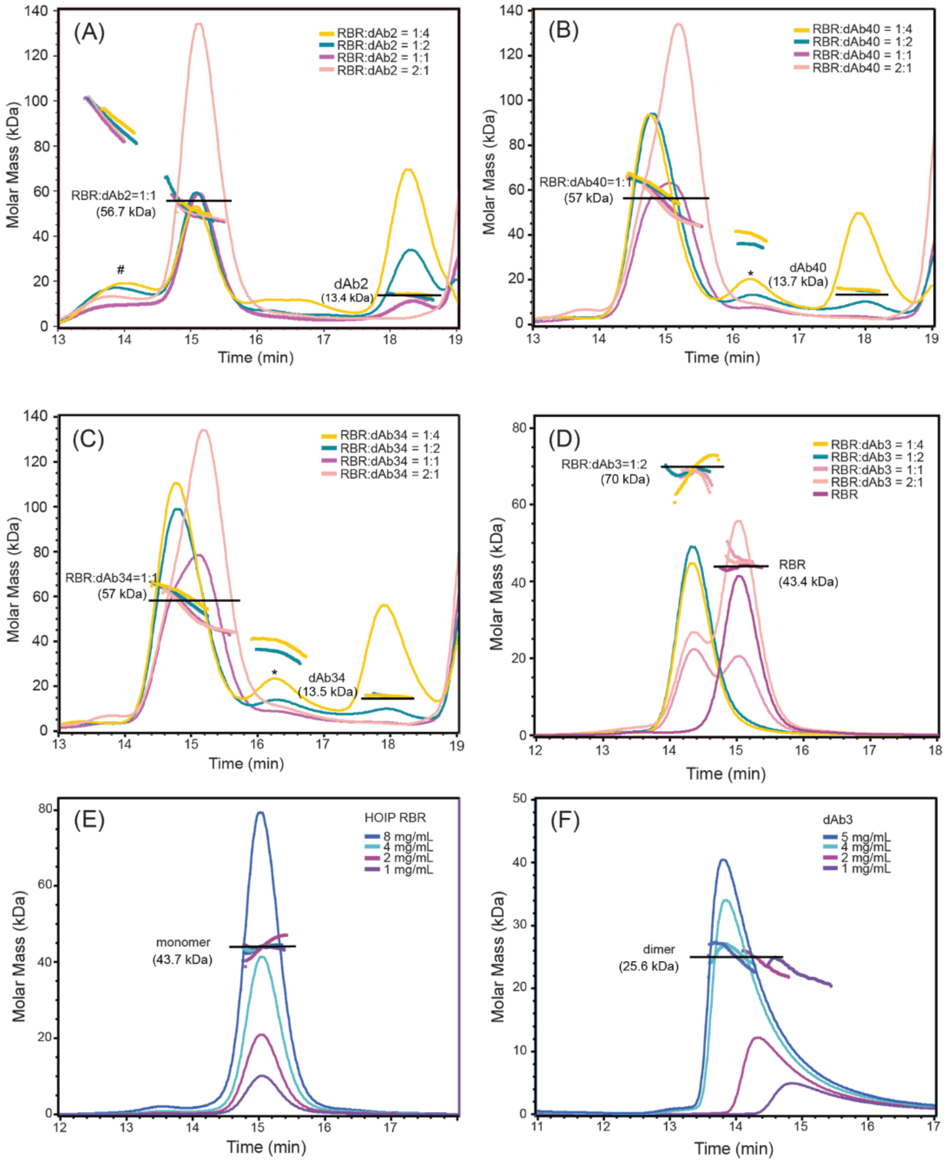

Figure S2. Related to Figure 1. SEC-MALLS analysis

(A) HOIP RBR and dAb2 (neutral dAb) were mixed at different ratios. All combinations demonstrated the same complex MW at 56.7 kDa, which is the MW of a 1:1 complex. # marks a HMW impurity present in the dAb2 preparation.
(B) HOIP RBR and dAb40 (neutral dAb) were mixed at different ratios. All combinations demonstrate the same complex MW at 57 kDa, which is the MW of a 1:1 complex. * marks a small fraction of self-associating dAb.
(C) HOIP RBR and dAb34 (differential dAb) were mixed at different ratios. All combinations demonstrate the same complex MW at 57 kDa, which is the MW of a 1:1 complex. * marks a small fraction of self-associating dAb.
(D) HOIP RBR and dAb3 were mixed at different ratios. HOIP RBR and dAb3 were saturated at a 1:2 ratio with an estimated complex MW at 70 kDa.
(E) HOIP RBR was analysed at 1-8 mg/mL. The estimated MW of HOIP RBR (43.7 kDa) is independent of the concentration indicating a monomeric behaviour in solution.
(F) dAb3 was analysed at 1-5 mg/mL. Protein at lower concentrations had longer retention times, which is likely due to sample-resin interaction under the buffer condition used. dAb3 is dimeric in solution.

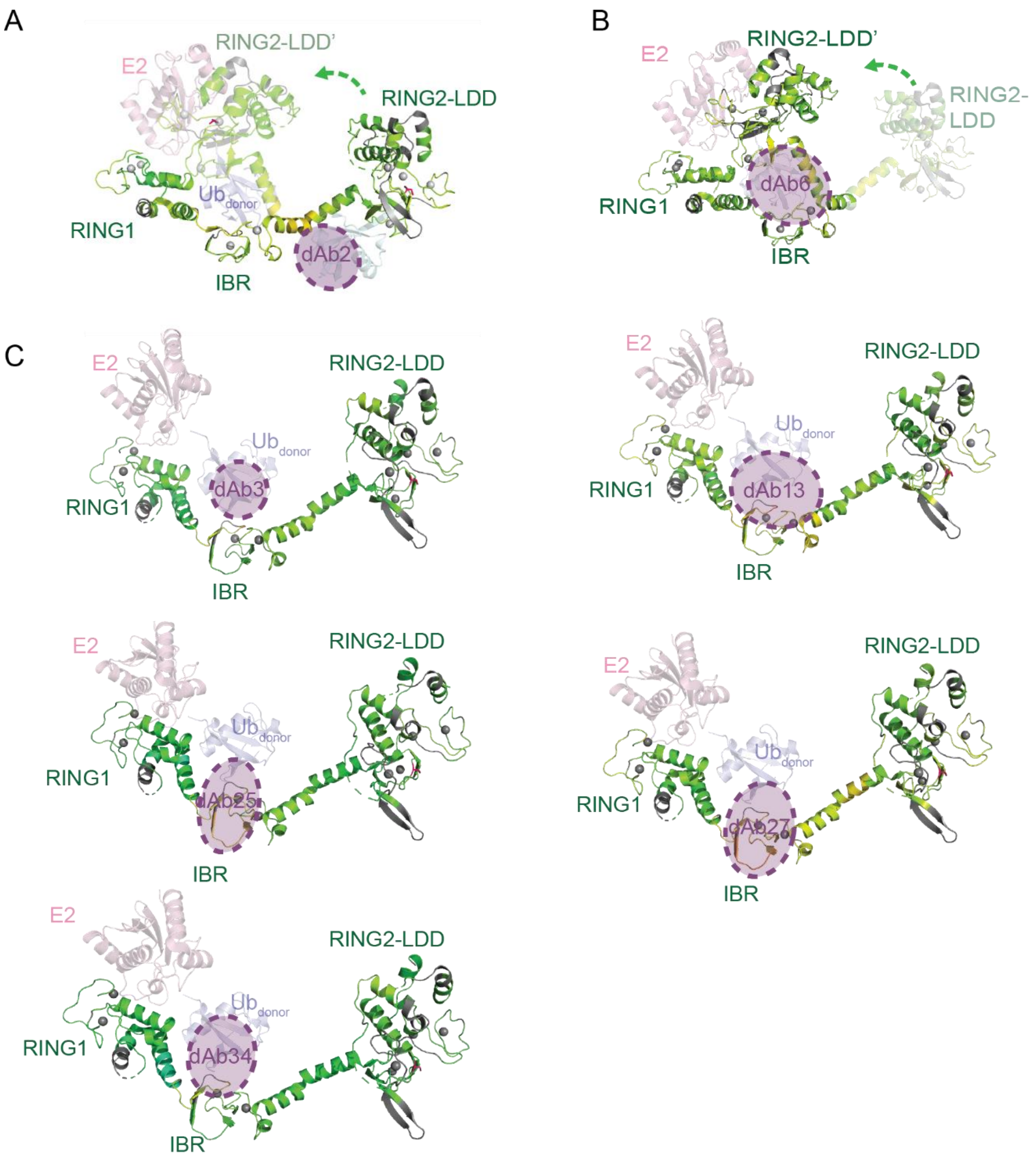

Figure S3. Related to Figure 2. Binding models of HOIP/dAb complexes

HDX-MS heatmaps displayed on the structure of HOIP RBR in complex with UbcH5B-Ub (PDB 5EDV). The elongated HOIP RBR molecule found in the crystal structure is indicated by RING2-LDD, whereas the closed form that is suggested to bind UbcH5B-Ub is indicated by RING2-LDD' (Lechtenberg et al., 2016). Structures are coloured according to the deuteration heatmap shown in Figure 2A, grey indicates regions for which no peptides were recovered.
(A) Model of possible binding mode of dAb2. For the neutral dAb2 it is suggested to bind to a site close to the linker between IBR and RING2-LDD.
(B) Model of possible binding mode of dAb6. Like dAb41 (Figure 2D) both inhibitory dAbs may bind close to active site C885 and possibly trap HOIP RBR in a closed conformation.
(C) Models of possible binding modes of dAb13, dAb25, dAb27 and dAb34, including dAb3 for comparison. These 5 differential dAbs are likely to bind close to the IBR and limit the flexibility of its adjacent linkers and thereby impact ubiquitin transfer.

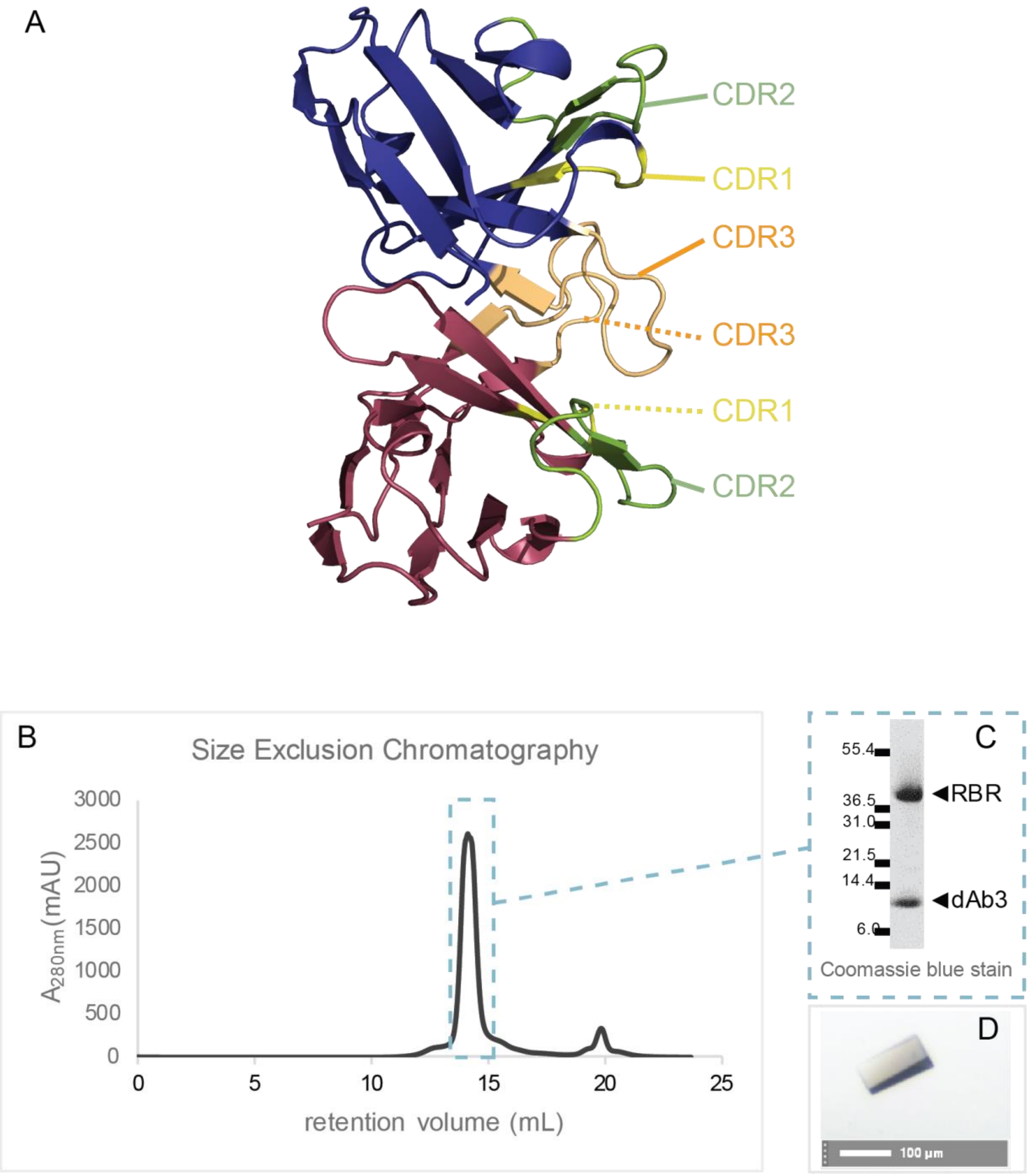

Figure S4. Related to Figure 3. Crystal structure of apo dAb3 and preparation of HOIP RBR/dAb3 complex for crystallization

(A) Colour scheme of the two dAb3 molecules (red and blue) is the same as presented in HOIP RBR/dAb3 structure in Figure 3A. CDR1 of the two dAbs is highlighted in yellow, CDR2 in green and CDR3 in orange.
(B) The HOIP RBR/dAb3 complex eluted in a single peak in size exclusion chromatography.
(C) The composition and purity of the complex examined by SDS-PAGE. The gel has been stained with Coomassie Blue and converted to grey scale.
(D) Crystal produced under the condition of 1.22 M ammonium sulfate, 30-100 mM NaCl, 0.1 M HEPES pH 7.0. Scale bar of 100 µm is shown.

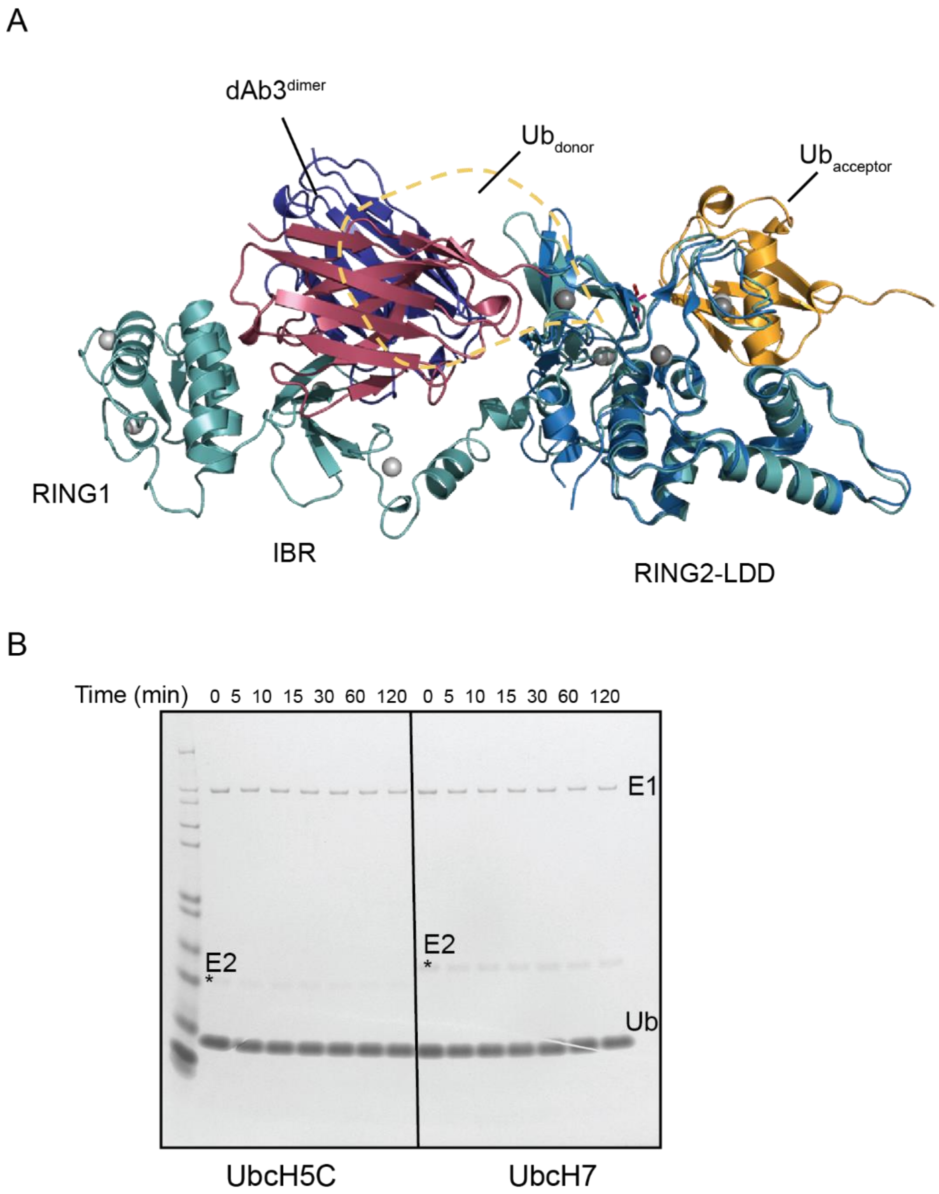

Figure S5. Related to Figures 1 and 4. Structural comparison of dAb-bound and ubiquitin-bound HOIP and control ubiquitination assays without E3 ligase

(A) Structural comparison of the HOIP RBR domain (in teal) in complex with dAb3 (in red and blue) and ubiquitin-bound RING2-LDD (4LJO, in light blue), overlapped via the RING2-LDD domain. The acceptor ubiquitin is shown in orange and the positon of the donor ubiquitin is indicated by yellow-dotted line.

(B) Control ubiquitination assays without E3 ligase to test for possible background activities of UbcH5C and UbcH7. Assays were carried out under the same conditions as those presented in Figure 1. The assays show that there is no background activity without the RBR domain of HOIP.

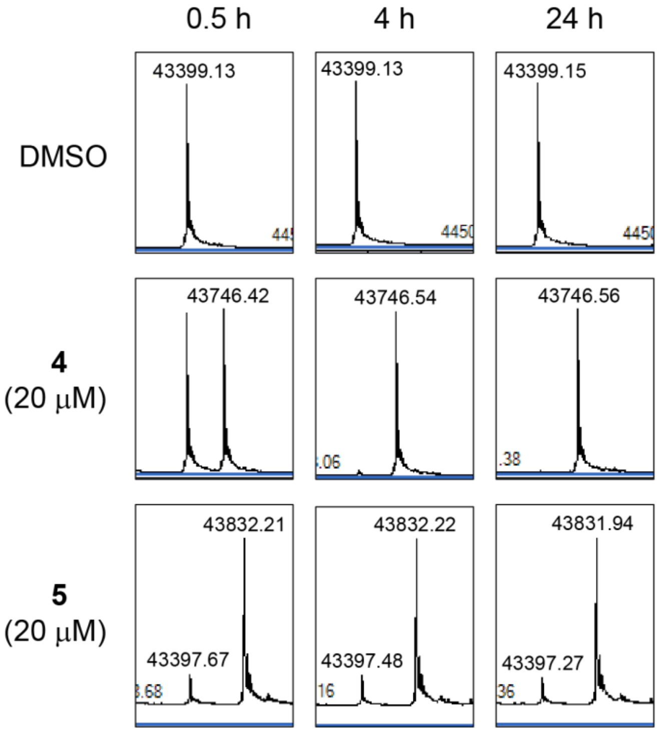

Figure S6. Related to Figure 5. Analysis of labelling of HOIP RBR (2 μM) by covalent compounds 4 and 5 (20 μM) after 0.5, 4 and 24 h incubation.

Table S1. Related to STAR Methods section of data collection and processing. Data collection and refinement statistics

	dAb3/HOIP-RBR (apo)	dAb3/HOIP-RBR - compound (**2**)	dAb3/HOIP-RBR - compound (**3**)	dAb3/HOIP-RBR - compound (**4**)	dAb3/HOIP-RBR - HOIPIN-8	dAb3 (apo)
PDB	6SC6	6SC5	6SC7	6SC8	6SC9	6T2J
**Data collection**						
Space group	I 2 2 2	I 2 2 2	I 2 2 2	I 2 2 2	I 2 2 2	C 1 2 1
Cell dimensions						
a, *b*, *c* (Å)	65.95, 87.49, 241.93	65.58, 86.66, 240.22	65.71, 86.70, 240.55	65.92, 87.77, 241.83	66.13, 87.05, 241.54	85.74, 59.31, 63.85
α, β, γ (°)	90, 90, 90	90, 90, 90	90, 90, 90	90, 90, 90	90, 90, 90	90, 131.97, 90
R_{merge}	2.86 (26.09)*	2.48 (26.38)	3.66 (50.46)	2.94 (77.70)	4.89 (70.14)	7.44 (38.36)
$I / \sigma I$	21.22 (4.97)	13.75 (2.29)	9.93 (1.33)	13.86 (0.77)	5.57 (1.02)	11.36 (2.78)
Completeness (%)	98.95 (96.62)	99.93 (99.90)	99.89 (99.60)	99.35 (95.48)	99.81 (98.51)	99.78 (99.35)
Multiplicity	2.0 (2.0)	2.0 (2.0)	2.0 (2.0)	2.0 (2.0)	2.0 (2.0)	4.8 (4.8)
**Refinement**						
Resolution (Å)	60.48 - 2.25 (2.33 - 2.25)	63.26 - 2.1 (2.18 - 2.1)	60.14 - 2.56 (2.65 - 2.56)	48.32 - 2.11 (2.18 - 2.11)	81.9 - 2.47 (2.56 - 2.47)	43.42 - 1.70 (1.76 - 1.70)
No. reflections	33399 (3205)	40481 (3997)	22647 (2218)	40843 (3847)	25596 (2510)	26230 (2586)
R_{work}	19.93 (29.17)	21.92 (29.57)	22.38 (31.74)	21.87 (38.34)	20.98 (31.78)	17.51 (23.64)
R_{free}	24.62 (34.88)	25.29 (34.91)	26.71 (36.43)	26.08 (40.96)	25.25 (39.55)	20.81 (27.95)
No. atoms						
Macromolecules	4724	4631	4620	4647	4597	1828
Ligands	35	50	91	50	52	5
Solvent	169	105	74	151	68	179
B-factors						
Macromolecules	52.28	56.87	66.14	66.05	67.36	17.0
Ligand/ion	75.47	66.30	73.60	76.85	70.14	40.4
Water	48.24	51.97	59.28	64.80	63.49	28.3
R.m.s. deviations						
Bond lengths (Å)	0.003	0.002	0.002	0.003	0.002	0.009
Bond angles (°)	0.61	0.46	0.47	0.57	0.50	1.04

*Values in parentheses are for highest-resolution shell.

Table S2. Related to STAR Methods section of oligonucleotides in key resources table.

Oligonucleotides		
HOIP-(697-1072)-AviTag FW primer	Sigma Aldrich Oligos	5'CAGGGACCCGGTCAGGAGTGTGCCGTGTGTGGC
HOIP-(697-1072)-AviTag RV primer 1	Sigma Aldrich Oligos	5'CTTCAAAGATGTCGTTCAGACCCTTCCGCCTGCGGGGGATACTC
HOIP-(697-1072)-AviTag RV primer 2	Sigma Aldrich Oligos	5'TTCGTGCCATTCGATTTTCTGCGCTTCAAAGATGTCGTTCAGACC
HOIP-(697-1072)-AviTag RV primer 3	Sigma Aldrich Oligos	5'GGCACCAGAGCGTTATTCGTGCCATTCGATTTTCTGCG
HOIP-(697-859)-AviTag FW primer	Sigma Aldrich Oligos	5'CCTCTTTCAGGGACCCGGTCAGGAGTGTGCCGTGTGTGGC
HOIP-(697-859)-AviTag RV primer 1	Sigma Aldrich Oligos	5'CTTCAAAGATGTCGTTCAGACCGCCCTGGGCCTGGTATTCTGGG
HOIP-(697-859)-AviTag RV primer 2	Sigma Aldrich Oligos	5'TTCGTGCCATTCGATTTTCTGCGCTTCAAAGATGTCGTTCAGACC
HOIP-(697-859)-AviTag RV primer 3	Sigma Aldrich Oligos	5'CCGCGTGGCACCAGAGCGTTATTCGTGCCATTCGATTTTCTGCG
HOIP-(748-859)-AviTag FW primer	Sigma Aldrich Oligos	5'CCTCTTTCAGGGACCCGGTGGCCGCCCCGACCTCACCG
HOIP-(748-859)-AviTag RV primer 1	Sigma Aldrich Oligos	5'CTTCAAAGATGTCGTTCAGACCGCCCTGGGCCTGGTATTCTGGG
HOIP-(748-859)-AviTag RV primer 2	Sigma Aldrich Oligos	5'TTCGTGCCATTCGATTTTCTGCGCTTCAAAGATGTCGTTCAGACC
HOIP-(748-859)-AviTag RV primer 3	Sigma Aldrich Oligos	5'CCGCGTGGCACCAGAGCGTTATTCGTGCCATTCGATTTTCTGCG
HOIP-(853-1072)-AviTag FW primer	Sigma Aldrich Oligos	5'CCTCTTTCAGGGACCCGGTCCAGAATACCAGGCCCAGGGCC
HOIP-(853-1072)-AviTag RV primer 1	Sigma Aldrich Oligos	5'CTTCAAAGATGTCGTTCAGACCCTTCCGCCTGCGGGGGATACTC
HOIP-(853-1072)-AviTag RV primer 2	Sigma Aldrich Oligos	5'TTCGTGCCATTCGATTTTCTGCGCTTCAAAGATGTCGTTCAGACC
HOIP-(853-1072)-AviTag RV primer 3	Sigma Aldrich Oligos	5'CCGCGTGGCACCAGAGCGTTATTCGTGCCATTCGATTTTCTGCG
DOM006	Sigma Aldrich Oligos	5'ATGGTTGTTGTCATTGTCGGCGCA
DOM008	Sigma Aldrich Oligos	5'AGCGGATAACAATTTCACACAGGA
DOM009	Sigma Aldrich Oligos	5'CGCCAGGGTTTTCCCAGTCACGAC
DOM57	Sigma Aldrich Oligos	5'ATGAGGTTTTGCTAAACAACTTTC
V_H clones FW primer	Sigma Aldrich Oligos	5'CCGTCTCGAGCTAATAAGAATTCACTGGCCGTCGT
V_H clones RV primer	Sigma Aldrich Oligos	5'GAATTCTTATTAGCTCGAGACGGTGACCAGGGTTCC
Vκ clones FW primer	Sigma Aldrich Oligos	5'GGAAATCGAACGGTAATAAGAATTCACTGGCCGTCGT
Vκ clones RV primer	Sigma Aldrich Oligos	5'GAATTCTTATTACCGTTCGATTTCCACCTTGGTCCC

Table S3. Related to STAR Methods section of soluble dAb construction. CDR sequences of select dAbs defined by Kabat numbering scheme

Clone ID	Clone Name		CDR1	CDR2	CDR3
CL-208735	49D077-26F11-1	dAb 3	GYSMA	TISPIGTYTYYADSVKG	GSYSRGTPF---------DY
CL-208673	49D077-26H02-1	dAb 2	FYSME	SIDAIGGRTYYADSVKG	VLPVSAENRF--------DY
CL-209011	49D077-30B01-1	dAb 6	DVDMG	AISEKGSGTYYADSVKG	SPSFGHF-----------DY
CL-209398	49D077-34E05-1	dAb 13	PAVMG	GIQEYGSKTYYADSVKG	DPYPRALGVF--------DV
CL-210069	49D077-42D01-1	dAb 18	RGWMY	SIPALGPDTYYADSVKG	RGSHGF------------DY
CL-210519	49D077-47F02-1	dAb 25	QPSML	SISGDGFHTYYADSVKG	TPTYAVEVLPVGEY----DV
CL-210525	49D077-47D03-1	dAb 27	GYSMI	SITEDGSATYYADSVKG	TPTYAVEVLPVGEY----DV
CL-209486	49D077-35E05-1	dAb 34	GRIMG	TISDEGPRTYYADSVKG	PVVLGYEGGPTPDF----DV
CL-210427	49D077-46B02-1	dAb 40	KAKMG	TIGHTGVPTYYADSVKG	PLPTSRPTGF--------DY
CL-209805	49D077-39D01-1	dAb 41	EYPMR	TISYDGSHTYYADSVKG	VILAQSSPLTRF------DY
