# Supplementary material for: Single-Domain Antibodies as Crystallization Chaperones to Enable Structure-Based Inhibitor Development for RBR E3 Ubiquitin Ligases
Source: Cell Chem Biol. 2020 Jan 16;27(1):83–93.e9. doi: 10.1016/j.chembiol.2019.11.007 (PMC6963773; doi:10.1016/j.chembiol.2019.11.007)

Data S1 - Related to *Compound Synthesis* in STAR Methods. Compound NMR and UPLC data.

2-(Methylamino)-2-oxoethyl (E)-4-(2-oxo-1,2,5,6,7,8-hexahydroquinoline-3-carboxamido)but-2-enoate (4)

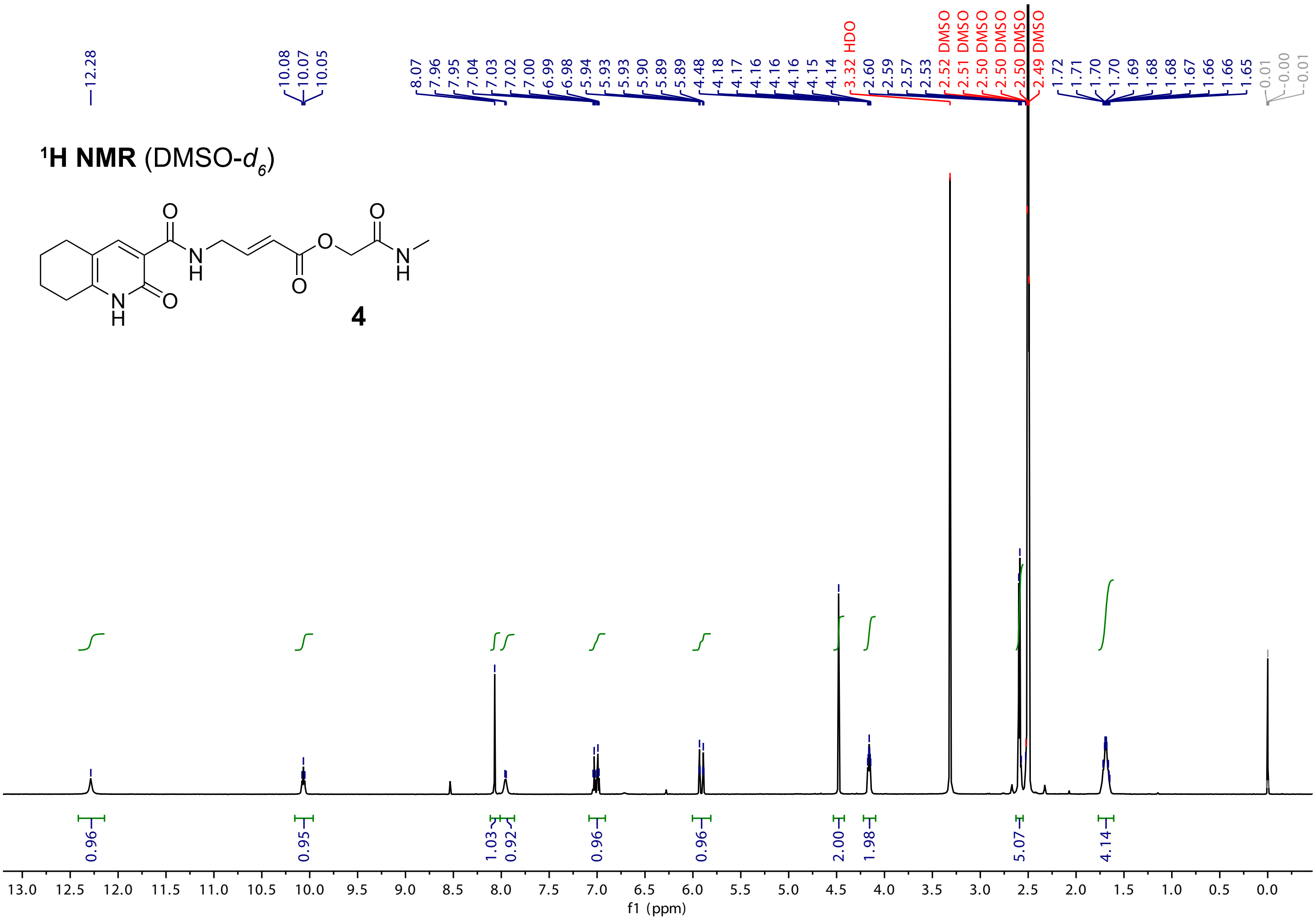

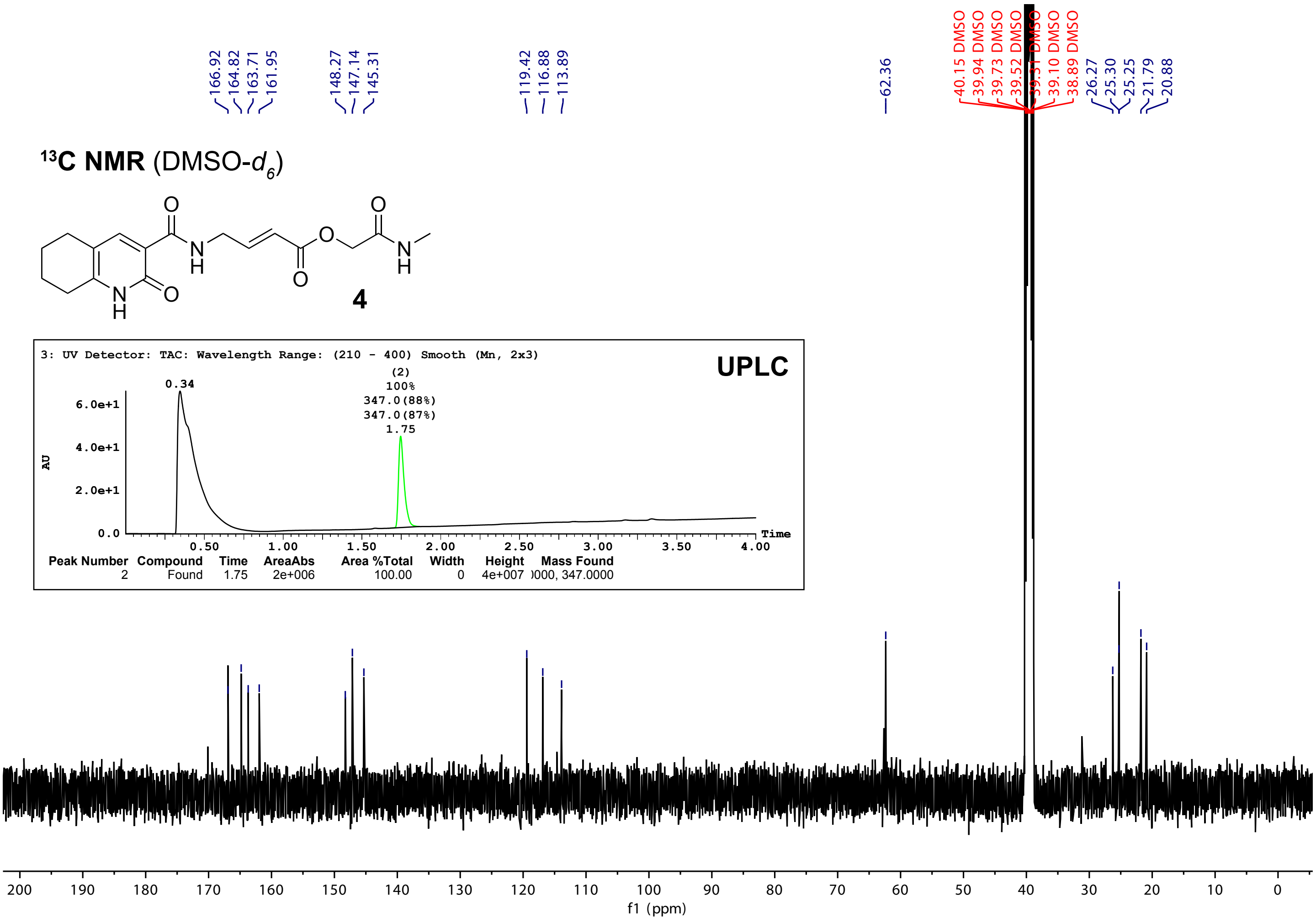

3,5-Dibromoisobenzofuran-1(3H)-one (SI-3-1)

^{1}H NMR ($CDCl_3$)

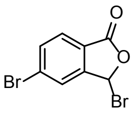

SI-3-1

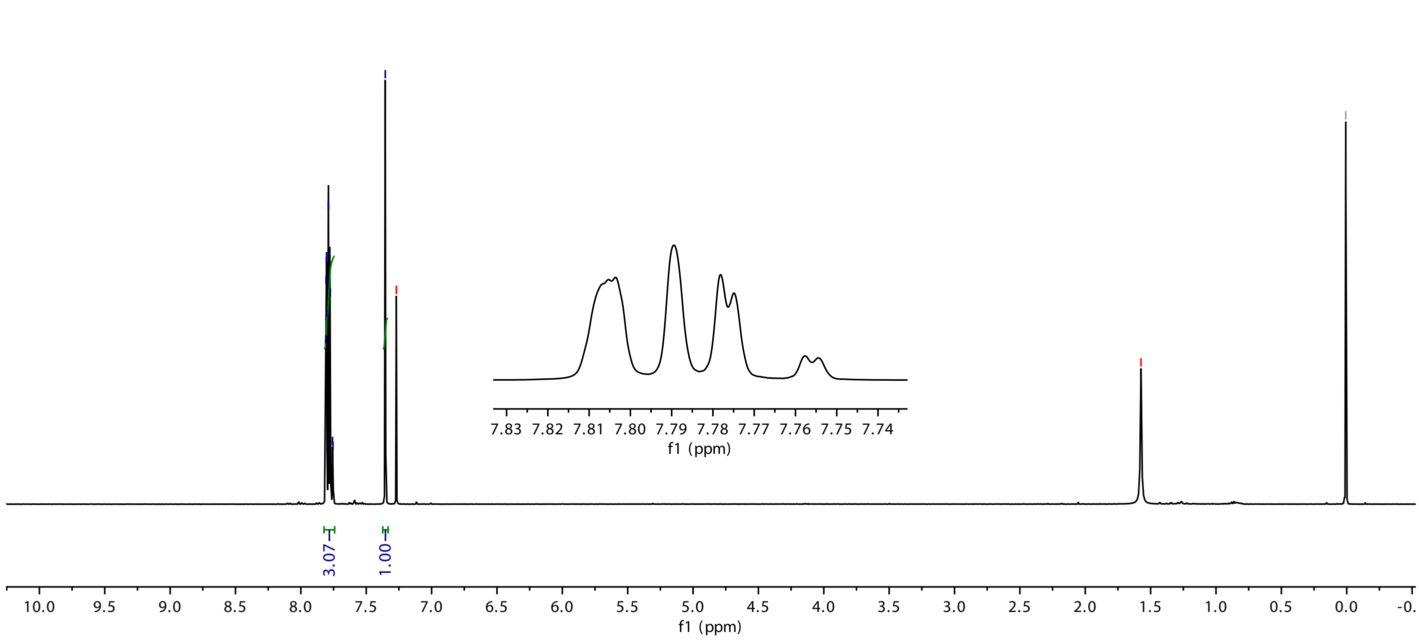

^{13}C NMR ($CDCl_3$)

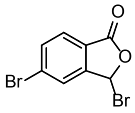

SI-3-1

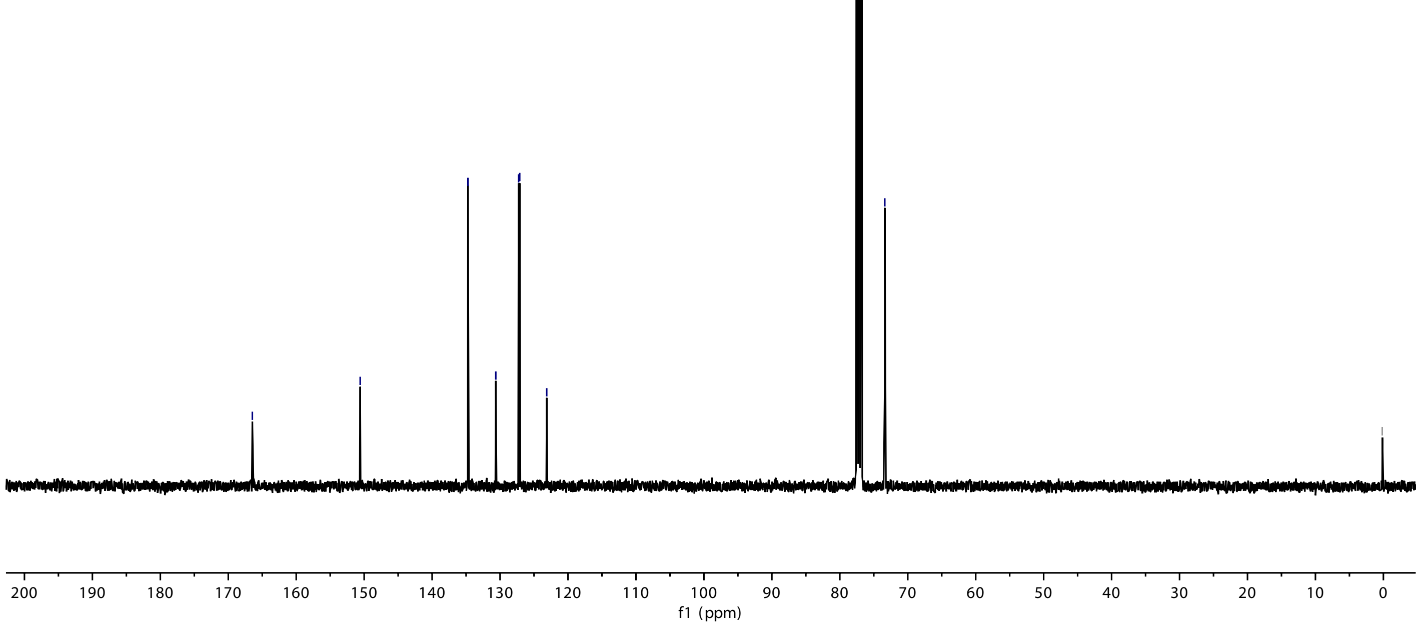

5-Bromo-3-hydroxyisobenzofuran-1(3H)-one (SI-3)

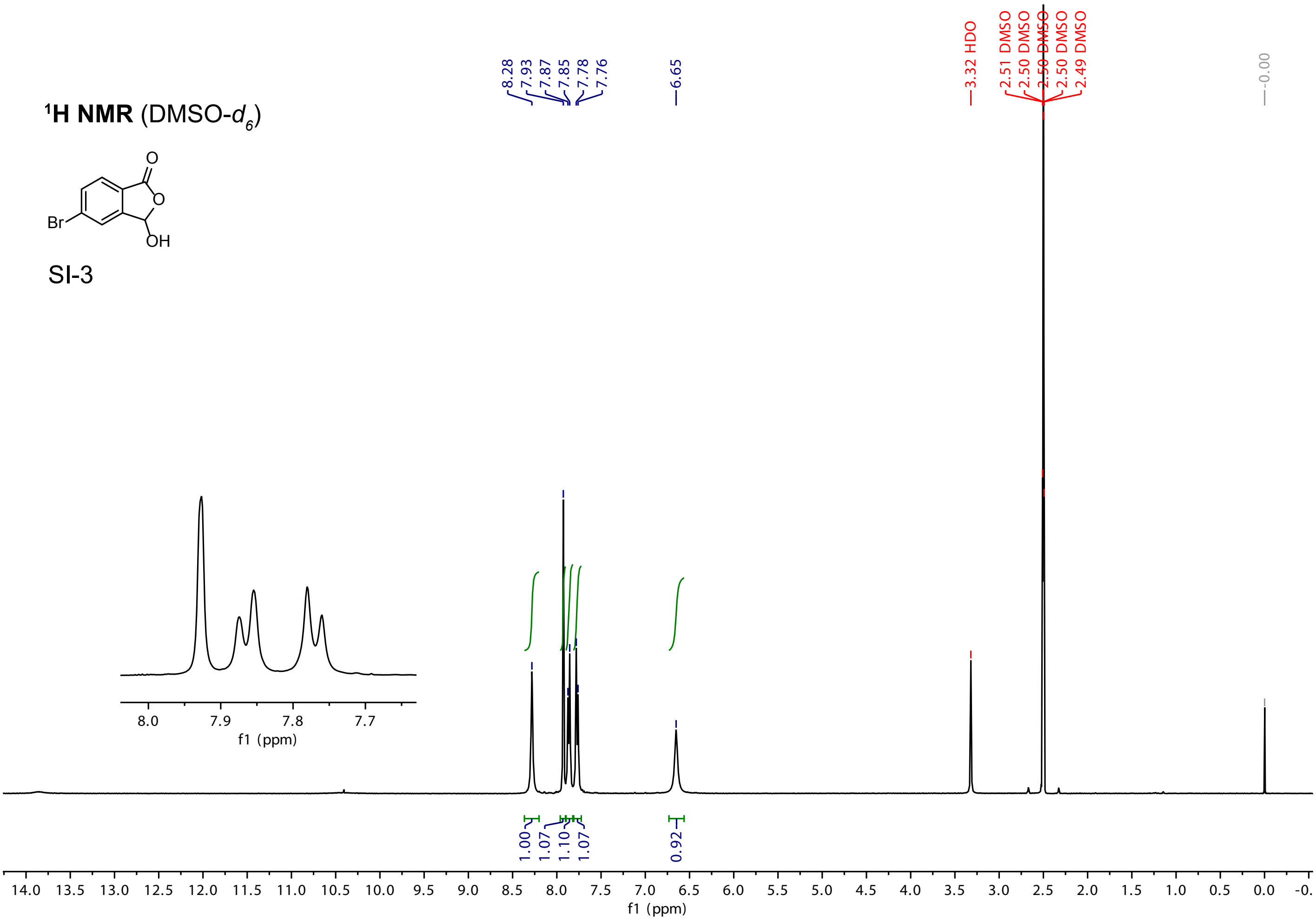

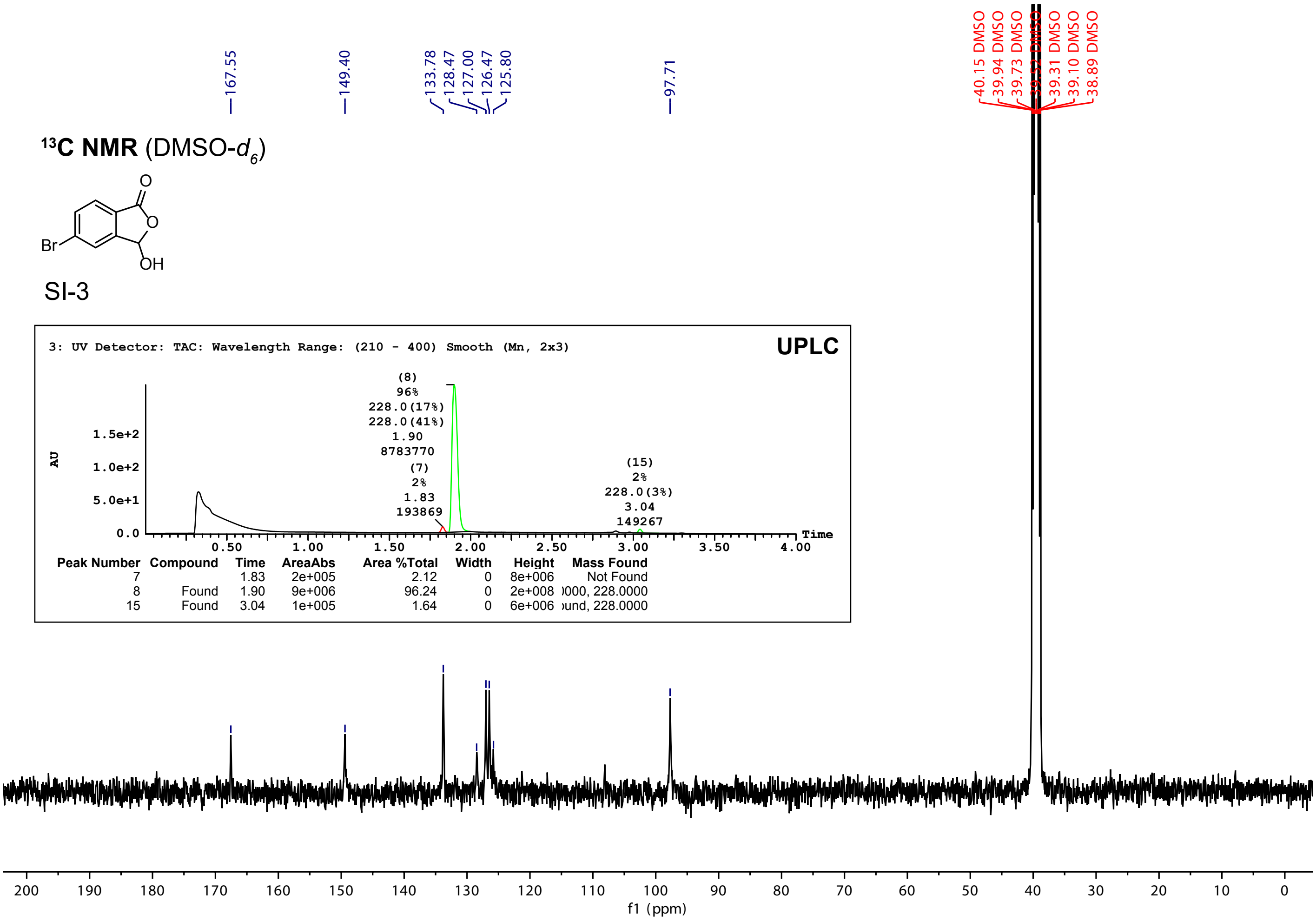

3-Hydroxy-5-(1-methyl-1H-pyrazol-4-yl)isobenzofuran-1(3H)-one (SI-4)

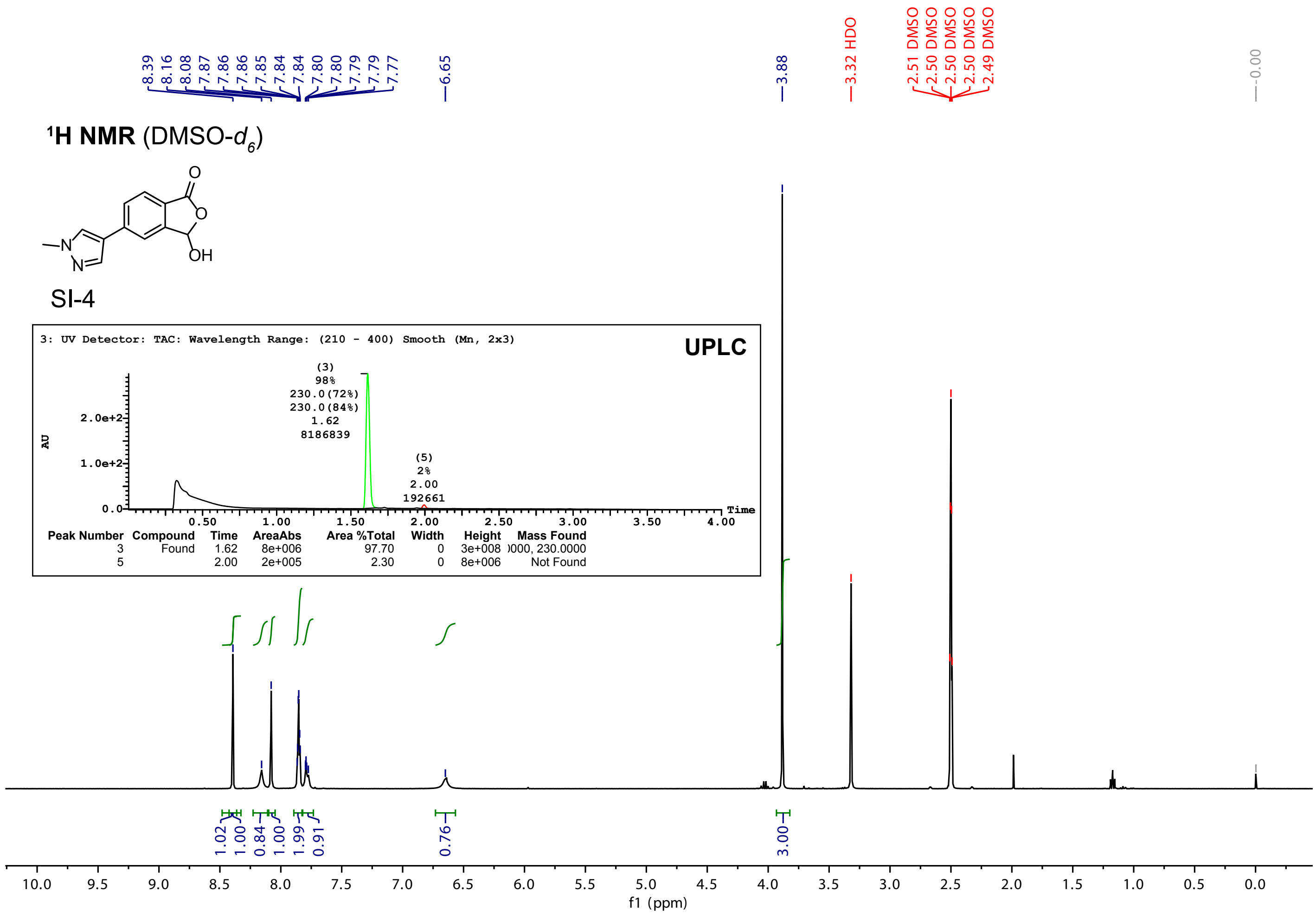

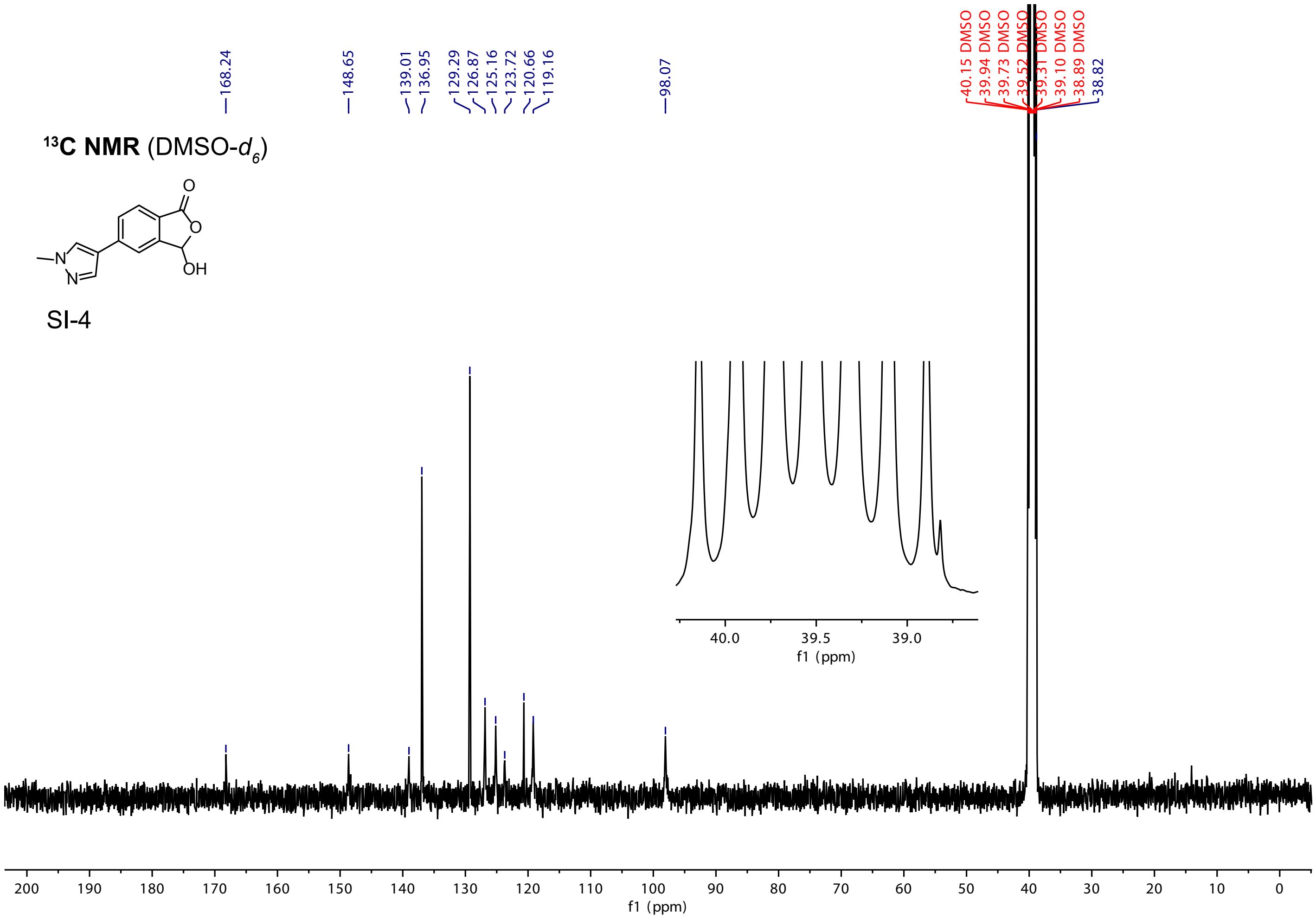

Sodium (E)-2-(3-(2,6-difluoro-4-(1H-pyrazol-4-yl)phenyl)-3-oxoprop-1-en-1-yl)-4-(1-methyl-1H-pyrazol-4-yl)-benzoate (5)

^{1}H NMR (DMSO-d_6)

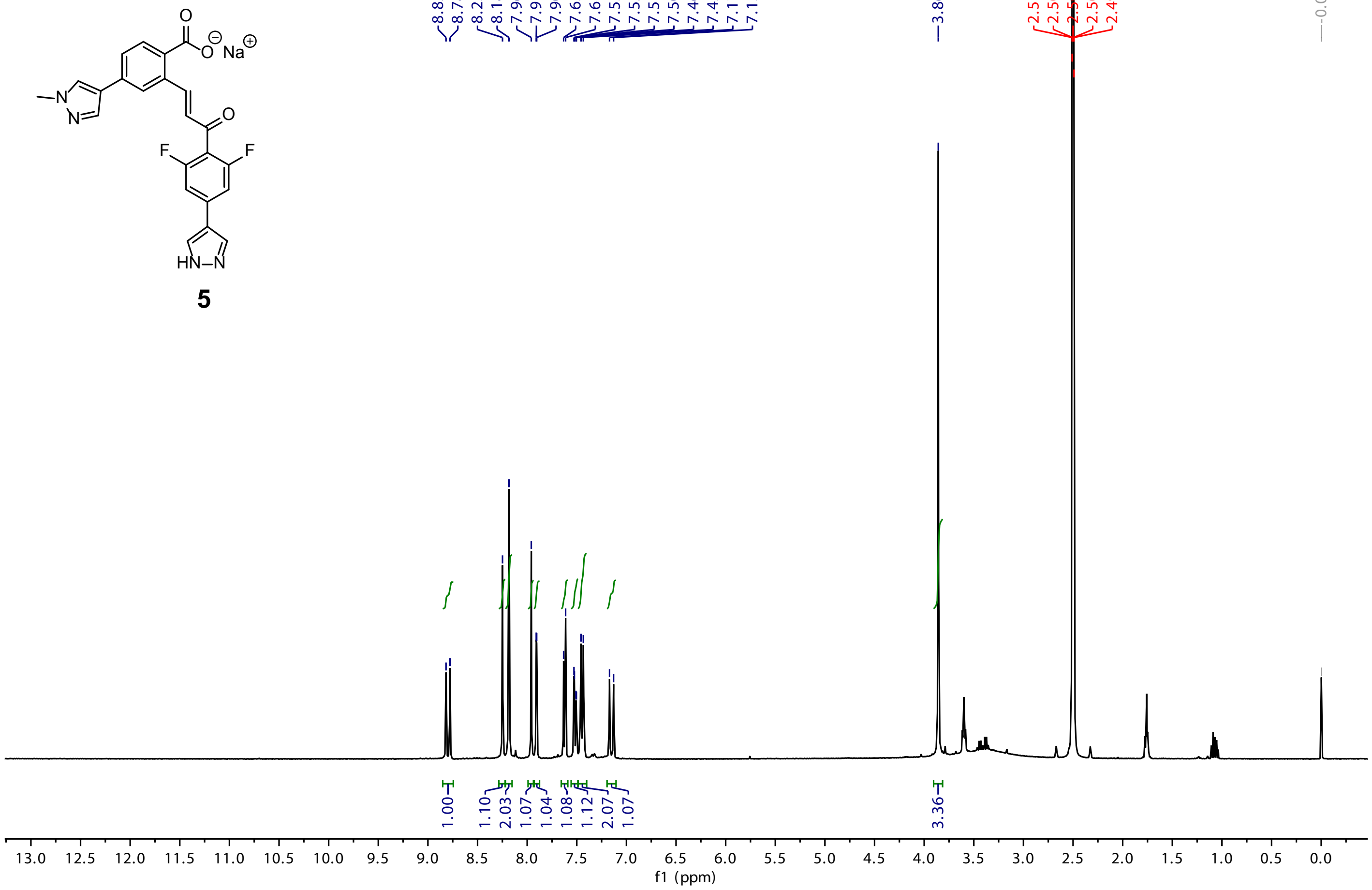

^{13}C NMR (DMSO-d_6)

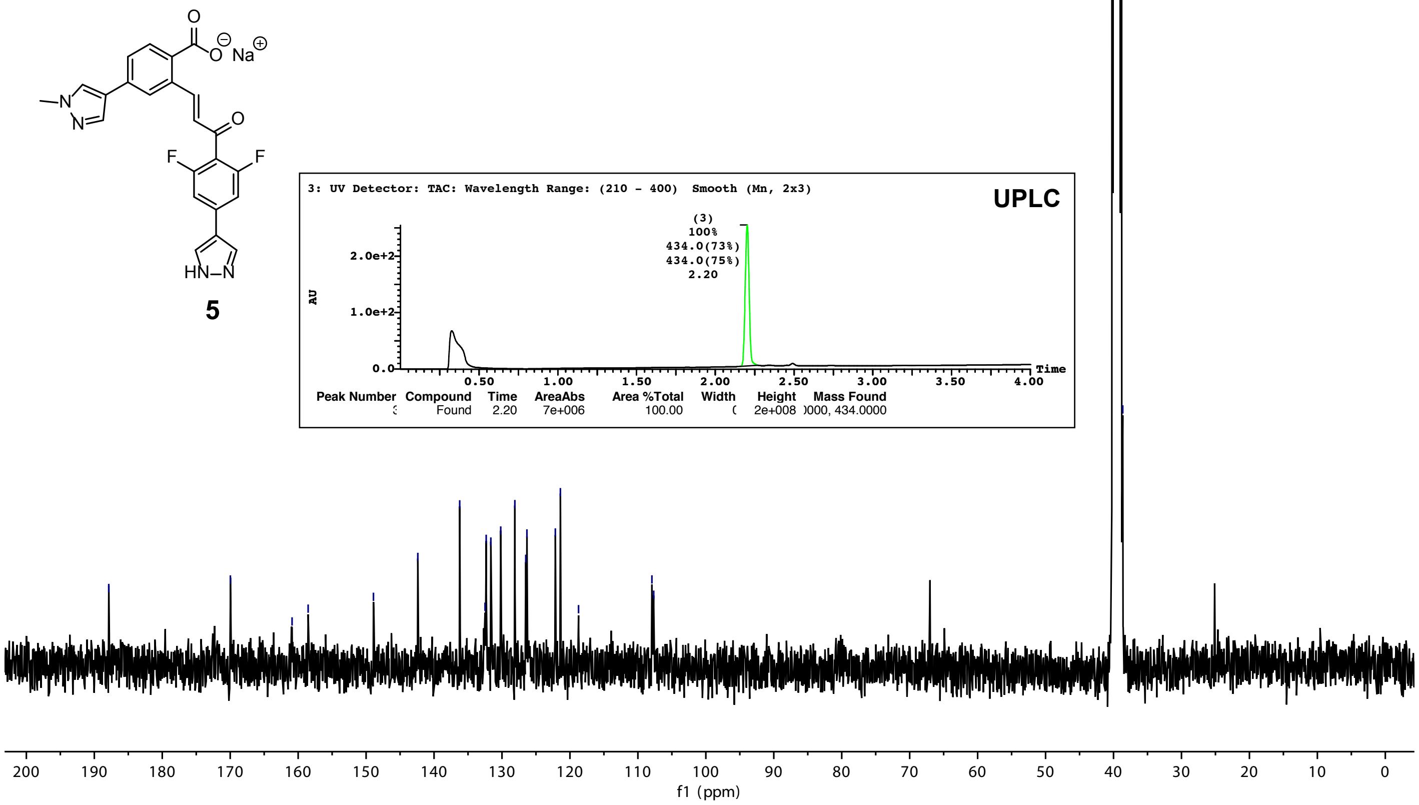

HSQC (DMSO-d_6)

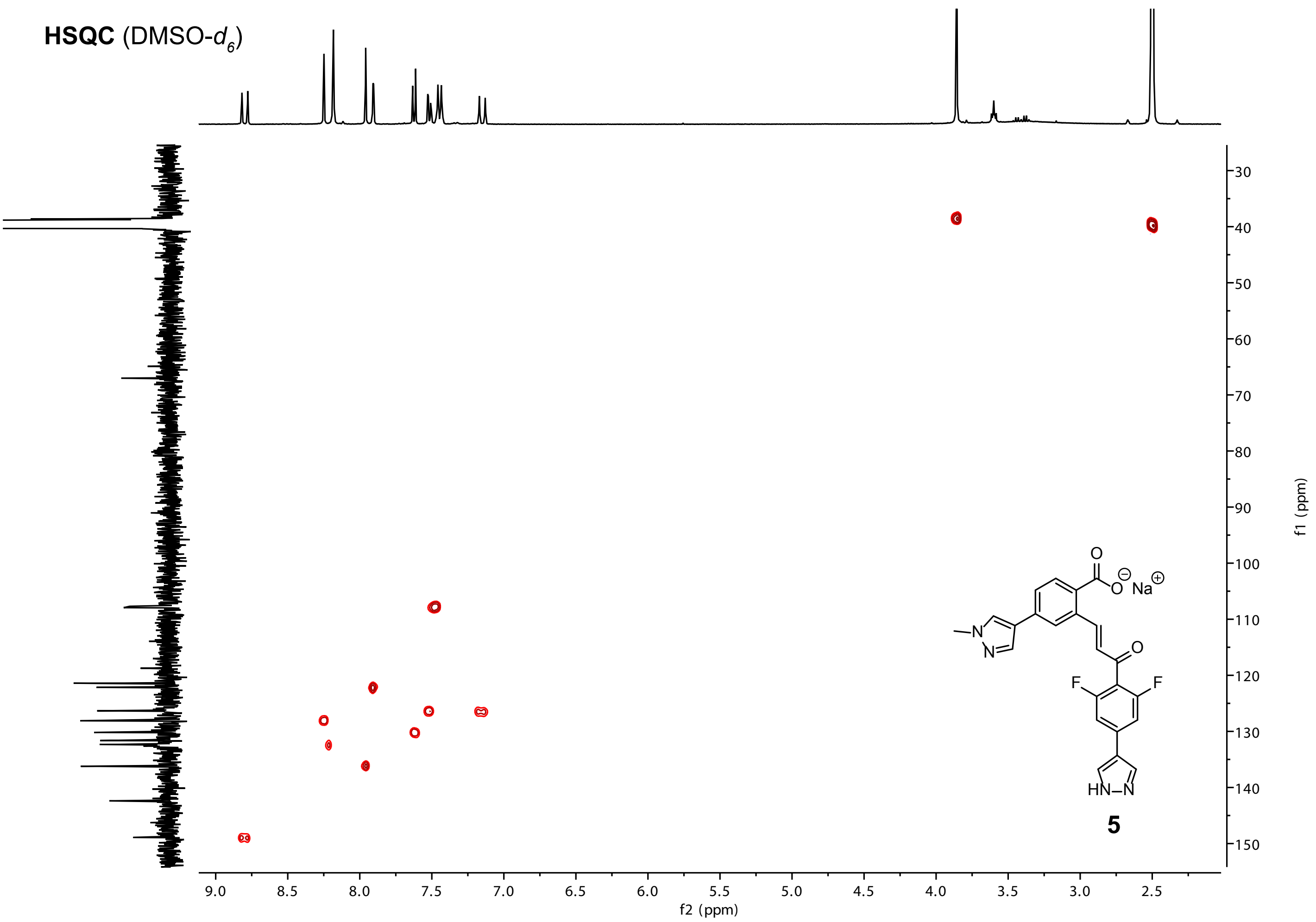

HMBC (DMSO-d_6)

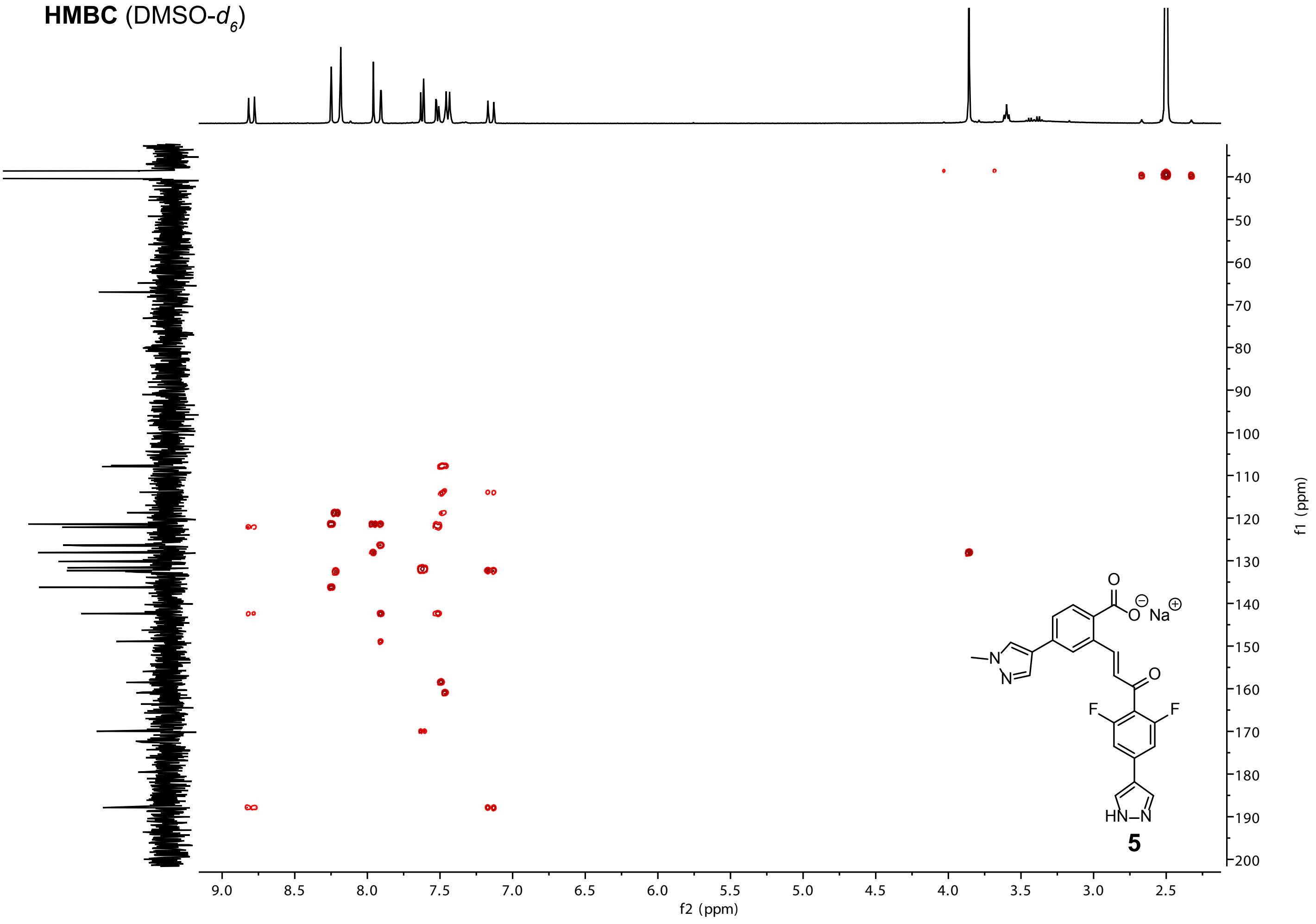

Supplement: Data S1. Compound NMR Spectra and UPLC Data Related to Compound Synthesis in the STAR Methods [file mmc2.pdf]
