# Supplementary material for: Single-Domain Antibodies as Crystallization Chaperones to Enable Structure-Based Inhibitor Development for RBR E3 Ubiquitin Ligases
Source: Cell Chem Biol. 2020 Jan 16;27(1):83–93.e9. doi: 10.1016/j.chembiol.2019.11.007 (PMC6963773; doi:10.1016/j.chembiol.2019.11.007)
Supplement: Scheme S1 Two-step synthesis of amide 4 from (E)-4-(2-oxo-1,2,5,6,7,8-hexahydroquinoline-3-carboxamido)but-2-enoic acid SI-1 [file mmc3.pdf]

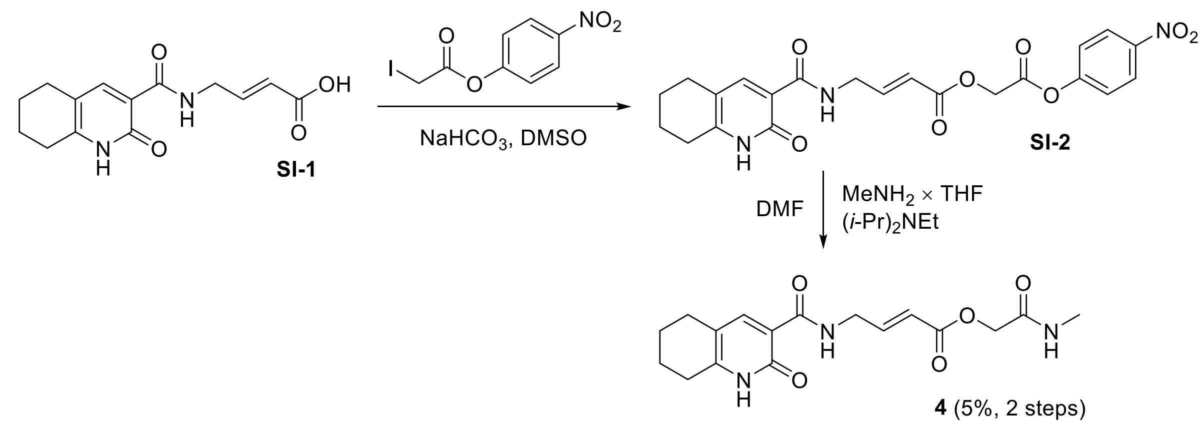

Scheme S1. Two-step synthesis of amide **4** from *(E)*-4-(2-oxo-1,2,5,6,7,8-hexahydroquinoline-3-carboxamido)but-2-enoic acid **SI-1**.
