# Supplementary material for: Single-Domain Antibodies as Crystallization Chaperones to Enable Structure-Based Inhibitor Development for RBR E3 Ubiquitin Ligases
Source: Cell Chem Biol. 2020 Jan 16;27(1):83–93.e9. doi: 10.1016/j.chembiol.2019.11.007 (PMC6963773; doi:10.1016/j.chembiol.2019.11.007)
Supplement: Scheme S2 Synthesis of inhibitor 5 (HOIPIN-8) according to the procedure reported by Katsuya et al. (2019). Boc, tert-butyloxycarbonyl; Bz, benzoyl; dppf, 1,1′-bis(diphenylphosphino)ferrocene; NBS, N-bromosuccinimide [file mmc4.pdf]

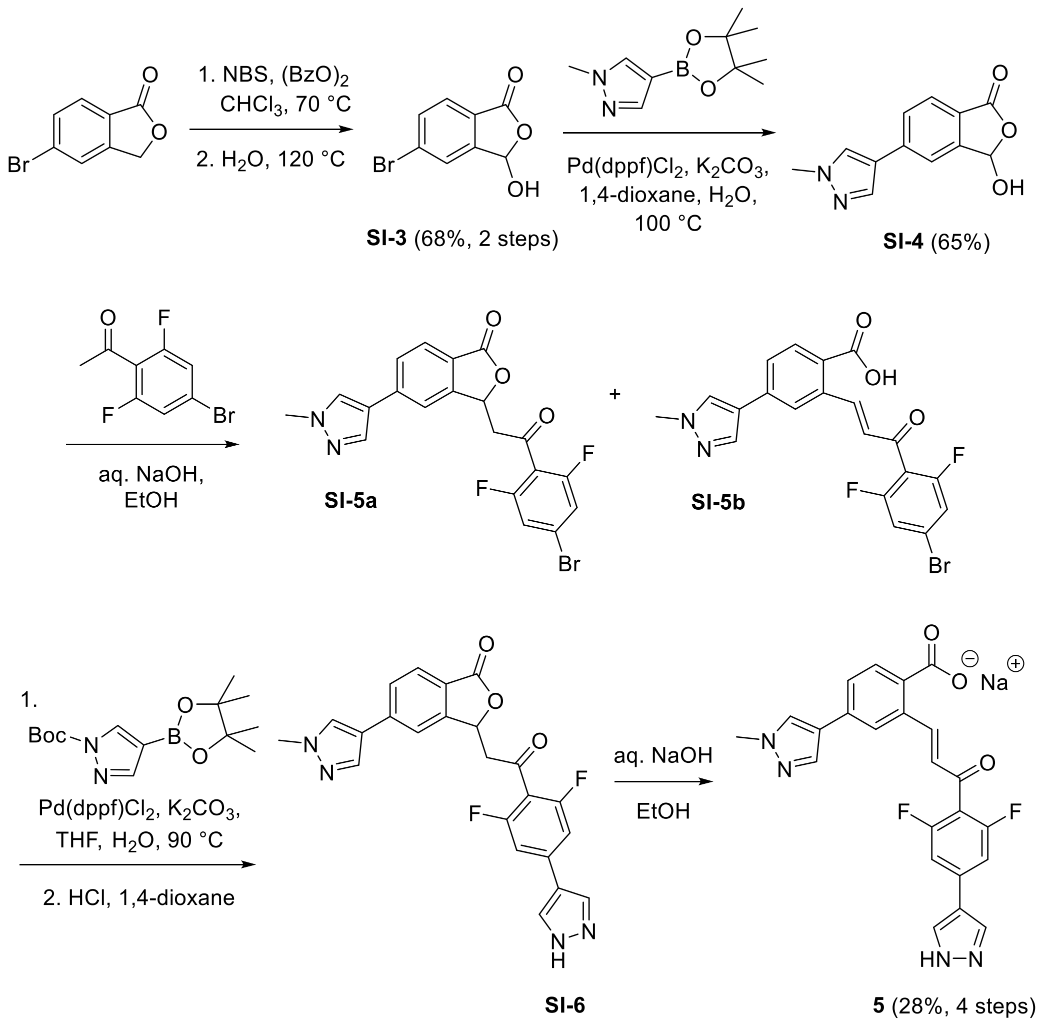

Scheme S2. Synthesis of inhibitor **5** (HOIPIN-8) according to the procedure reported by Katsuya *et al*. (Katsuya et al., 2019) Boc = *tert*-butyloxycarbonyl; Bz = benzoyl; dppf = 1,1'-Bis(diphenylphosphino)ferrocene; NBS = *N*-bromosuccinimide.
