# Supplementary material for: Single-Domain Antibodies as Crystallization Chaperones to Enable Structure-Based Inhibitor Development for RBR E3 Ubiquitin Ligases
Source: Cell Chem Biol. 2020 Jan 16;27(1):83–93.e9. doi: 10.1016/j.chembiol.2019.11.007 (PMC6963773; doi:10.1016/j.chembiol.2019.11.007)
Supplement: Document S2. Article plus Supplemental Information [file mmc5.pdf]

Resource

Cell Chemical Biology

Single-Domain Antibodies as Crystallization Chaperones to Enable Structure-Based Inhibitor Development for RBR E3 Ubiquitin Ligases

Graphical Abstract

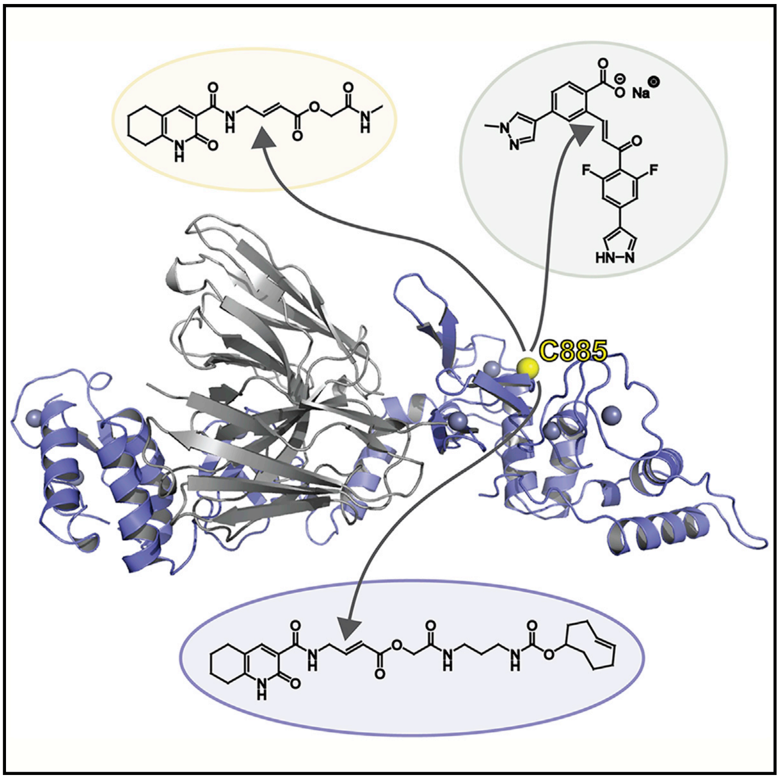

Authors

Yi-Chun Isabella Tsai,
Henrik Johansson, David Dixon, ...,
Jane Clarkson, David House,
Katrin Rittinger

Correspondence

katrin.rittinger@crick.ac.uk

In Brief

Tsai et al. identify single-domain antibodies (dAbs) that modulate the activity of the E3 ligase HOIP and facilitate crystallization of its catalytic RBR domain. HOIP/dAb crystals serve as a robust platform for ligand soaking and provide a novel tool for structure-based inhibitor development of E3 ligases.

Highlights

- Single-domain antibodies (dAbs) modulate the activity of the E3 ligase HOIP
- dAbs facilitate HOIP RBR domain crystallization and ligand soaking
- HOIP/dAb complex crystals provide access to structure-based inhibitor development

Tsai et al., 2020, Cell Chemical Biology *27*, 83–93
January 16, 2020 © 2019 The Author(s). Published by Elsevier Ltd.
https://doi.org/10.1016/j.chembiol.2019.11.007

CellPress

Cell Chemical Biology
Resource

Single-Domain Antibodies as Crystallization Chaperones to Enable Structure-Based Inhibitor Development for RBR E3 Ubiquitin Ligases

Yi-Chun Isabella Tsai,[1] Henrik Johansson,[1,2] David Dixon,[3] Stephen Martin,[4] Chun-wa Chung,[2,3] Jane Clarkson,[3] David House,[2] and Katrin Rittinger[1,5,*]
[1]Molecular Structure of Cell Signalling Laboratory, The Francis Crick Institute, 1 Midland Road, London NW1 1AT, UK
[2]Crick-GSK Biomedical LinkLabs, GlaxoSmithKline, Gunnels Wood Road, Stevenage SG1 2NY, UK
[3]R&D Medicinal Science & Technology, GlaxoSmithKline, Gunnels Wood Road, Stevenage SG1 2NY, UK
[4]Structural Biology Science Technology Platform, The Francis Crick Institute, 1 Midland Road, London NW1 1AT, UK
[5]Lead Contact
*Correspondence: katrin.rittinger@crick.ac.uk
https://doi.org/10.1016/j.chembiol.2019.11.007

SUMMARY

Protein ubiquitination plays a key role in the regulation of cellular processes, and misregulation of the ubiquitin system is linked to many diseases. So far, development of tool compounds that target enzymes of the ubiquitin system has been slow and only a few specific inhibitors are available. Here, we report the selection of single-domain antibodies (single-dAbs) based on a human scaffold that recognize the catalytic domain of HOIP, a subunit of the multi-component E3 LUBAC and member of the RBR family of E3 ligases. Some of these dAbs affect ligase activity and provide mechanistic insight into the ubiquitin transfer mechanism of different E2-conjugating enzymes. Furthermore, we show that the co-crystal structure of a HOIP RBR/dAb complex serves as a robust platform for soaking of ligands that target the active site cysteine of HOIP, thereby providing easy access to structure-based ligand design for this important class of E3 ligases.

INTRODUCTION

Ubiquitin modification of protein substrates provides signals for a myriad of cellular responses, from protein degradation to DNA repair or immune signaling. Ubiquitin can be attached to the substrate in the form of a single ubiquitin molecule, or can form polyubiquitin chains via one of its seven Lys residues or the N-terminal methionine (M1) amine group. Substrate modification with ubiquitin requires an enzymatic cascade involving E1-activating enzymes, E2-conjugating enzymes, and E3 ubiquitin ligases. Ubiquitin is first activated in an ATP-dependent manner to form an E1-Ub conjugate and subsequently transferred onto an E2-conjugating enzyme. E3 ligases mediate substrate selection, while specific combinations of E2 and E3 enzymes can determine the type of ubiquitin modification formed (Hershko and Ciechanover, 1998; Komander and Rape, 2012; Yau and Rape, 2016). A multi-protein E3 complex called LUBAC (linear ubiquitin chain assembly complex) is the only known E3 ligase able to generate polyubiquitin chains via M1 (Kirisako et al., 2006). These unique chains play important roles in the regulation of immune signaling pathways via the activation of nuclear factor κB and apoptotic cell death (Gerlach et al., 2011; Ikeda et al., 2011; Rittinger and Ikeda, 2017; Tokunaga et al., 2009, 2011). Interestingly, inhibition of LUBAC activity has been shown to re-sensitize cells that have become resistant to treatment with cisplatin, suggesting that targeting of LUBAC could be of therapeutic benefit (MacKay et al., 2014; Ruiz et al., 2019). LUBAC consists of HOIP and HOIL-1L, two RBR (RING-between-RING) domain-containing proteins, and SHARPIN (Gerlach et al., 2011; Ikeda et al., 2011; Tokunaga et al., 2011). The active core of LUBAC is located within the HOIP RBR domain, which cooperates with a C-terminal extension (the linear chain determining domain [LDD]) to provide chain linkage specificity (Smit et al., 2012; Stieglitz et al., 2012, 2013). RBR ligases function via a hybrid mechanism that combines mechanistic features from RING and HECT E3 ligases (Wenzel et al., 2011). RING-type E3s simultaneously recognize the ubiquitin-loaded E2 (E2-Ub) and substrate, and facilitate ubiquitin transfer directly from E2 to the substrate. In contrast, HECT-type ligases first transfer ubiquitin from E2 to an active site cysteine on the HECT domain to form a E3-Ub thioester intermediate before transfer onto the substrate (Berndsen and Wolberger, 2014; Buetow and Huang, 2016; Deshaies and Joazeiro, 2009). RBR family members share a common RING1-IBR-RING2 catalytic RBR domain organization where RING1 recognizes E2-Ub in a manner similar to canonical RING domains (Dove and Klevit, 2017; Walden and Rittinger, 2018; Wenzel et al., 2011). In contrast, RING2 is structurally close to an IBR-fold and encompasses a catalytic cysteine that forms a ubiquitin thioester intermediate before ubiquitin transfer onto a substrate (Stieglitz et al., 2013).

At present, there is a paucity of validated tool compounds that can be used to study the function of LUBAC and other RBR ligases in a cellular context. BAY11-7082 and gliotoxin both have been described to inhibit LUBAC activity, but lack substrate specificity (Sakamoto et al., 2015; Strickson et al., 2013). More recently, a compound called HOIPIN-1 and derivatives thereof have been described that show sub-micromolar potency against

This is an open access article under the CC BY license (http://creativecommons.org/licenses/by/4.0/).

Table 1. Dissociation Constants of HOIP RBR/dAb Complexes

RBR/dAb	k_{on} (M^{-1} s^{-1})	k_{off} (s^{-1})	K_D (nM)
RBR/dAb2	$1.9 \times 10^4 \pm 6.7 \times 10^2$	$1.4 \times 10^{-4} \pm 1.2 \times 10^{-5}$	7.5 ± 0.7
RBR/dAb3	$9.4 \times 10^5 \pm 8.3 \times 10^4$	$8.3 \times 10^{-2} \pm 5.5 \times 10^{-2}$	2.9 ± 0.3
RBR/dAb6	$3.8 \times 10^4 \pm 8.2 \times 10^2$	$4.8 \times 10^{-4} \pm 6.6 \times 10^{-5}$	13 ± 1.8
RBR/dAb13	$8.6 \times 10^3 \pm 5.0 \times 10^2$	$6.5 \times 10^{-4} \pm 8.0 \times 10^{-5}$	76 ± 10
RBR/dAb18	$7.5 \times 10^3 \pm 4.3 \times 10^2$	$3.8 \times 10^{-4} \pm 4.3 \times 10^{-5}$	51 ± 6.4
RBR/dAb25	$3.0 \times 10^4 \pm 9.1 \times 10^2$	$1.8 \times 10^{-3} \pm 1.7 \times 10^{-4}$	62 ± 6.2
RBR/dAb27	$1.3 \times 10^4 \pm 5.9 \times 10^2$	$2.3 \times 10^{-3} \pm 2.7 \times 10^{-4}$	170 ± 21
RBR/dAb34	$7.3 \times 10^3 \pm 2.6 \times 10^2$	$1.2 \times 10^{-2} \pm 1.4 \times 10^{-3}$	1700 ± 210
RBR/dAb40	$6.4 \times 10^3 \pm 2.5 \times 10^2$	$2.0 \times 10^{-3} \pm 2.0 \times 10^{-4}$	320 ± 33
RBR/dAb41	$2.5 \times 10^5 \pm 8.7 \times 10^3$	$7.9 \times 10^{-4} \pm 1.2 \times 10^{-4}$	3.2 ± 0.5

LUBAC, but their selectivity and stability have not been characterized (Katsuya et al., 2019). As an alternative to generate small-molecule tool compounds against LUBAC we have recently employed a fragment-based covalent ligand screening approach to identify hits that could be further developed into inhibitors against HOIP, and other active site cysteine-containing E3 ligases. A high-resolution ligand-bound RING2-LDD crystal structure validated our pilot compound and provided a molecular basis for the design of improved molecules (Johansson et al., 2019). However, crystallization of the RING2-LDD fragment was difficult; crystals grown were fragile and X-ray diffraction was highly anisotropic, making this approach unsuitable for routine access to HOIP-small-molecule structures. Similarly, determination of the crystal structure of a complex between the entire RBR domain of HOIP and an UbcH5B-Ub conjugate was very challenging and laborious due to crystal morphology (Lechtenberg et al., 2016). Hence, neither route has the throughput needed to support structure-based inhibitor design and therefore a better validation platform for inhibitor optimization against HOIP is needed.

We aimed to develop single chain antibody-based crystallization chaperones to assist structural studies on the HOIP RBR domain in a straightforward fashion, at high resolution. Furthermore, we wanted to test if single-chain antibodies could be used as activity modulators to provide mechanistic insight into E3 ligase function. Cameloid-derived nanobodies have proved very useful tools to assist crystallizing complex protein architectures; for example, a ubiquitin-bound kinase PINK (Schubert et al., 2017) and active G protein-coupled receptor conformations (Steyaert and Kobilka, 2011). To avoid the need for immunization, we decided to test if synthetic domain antibody (dAb) libraries based on human scaffolds (Enever et al., 2014; Ignatovich et al., 2012) may also serve as crystallization chaperones. Synthetic libraries have the advantage of overcoming poorly immunogenic targets and allow selection in the presence of ligands; however, use of human dAbs as crystallization chaperones is as yet largely unexplored.

Herein, we report the screening of a large synthetic single-dAb library against the RBR of HOIP to successfully obtain tight-binding dAbs which display different functional effects on E3 ligase activity, depending on the identity of the E2-conjugating enzyme. Moreover, we solved the co-crystal structure of a HOIP RBR/dAb complex and demonstrate that an RBR E3 ligase can serve as a robust platform for soaking of ligands, an expedient approach for structure-based ligand design that should aid the development of modulators of this important class of enzymes.

RESULTS

Single-Domain Antibodies Bind HOIP RBR with High Affinities

Domain antibody screening was based on the selection of phage dAb libraries (Ignatovich et al., 2012) against biotinylated antigen captured on magnetic affinity beads. A C-terminal AviTag was introduced to the target protein, HOIP RBR (residues 697–1,072), to allow biotinylation *in vitro* on the Lys residue within the AviTag peptide. Biotinylated HOIP RBR was immobilized on streptavidin resin and three rounds of phage display selection were carried out. Phage ELISAs were evaluated following second and third rounds of selection to determine the enrichment of antigen-specific phage. Sequences of randomly selected clones from second and third rounds were analyzed to confirm binder diversity. After obtaining several hundreds of unique dAb candidates, biolayer interferometry (BLI) was employed to evaluate dAb binding to HOIP RBR. Full-length or truncated versions of RBR were included in the evaluation process to eliminate dAbs that only bind RING1 or RING2-LDD domains, because those dAbs are less likely to contribute toward stabilization of the flexible linkers that connect RBR subdomains. Other selection criteria included fast-association and slow-dissociation rates to identify tight and stable binders. More than 80 binders were selected and purified in soluble form for further assessment, including association/dissociation rate evaluation by BLI, dAb oligomerization state evaluation by SEC-MALLS, and dAb and dAb/RBR thermal stability evaluation by differential scanning fluorimetry. Finally, 10 dAbs were selected to be taken forward and their interaction with HOIP RBR was quantified by BLI, which showed that most of the binding affinities (K_D) of the selected dAbs are in the nanomolar range (Table 1; Figure S1).

Differential Functional Effects of dAbs on HOIP Activity

Members of the RBR family of E3 ligases work with two types of E2-conjugating enzymes: UbcH7, which is strictly cysteine-reactive and not active with RING-type E3s (although in some cases forms stable complexes) and lysine-reactive E2s, especially members of the UbcH5 family, which are highly promiscuous

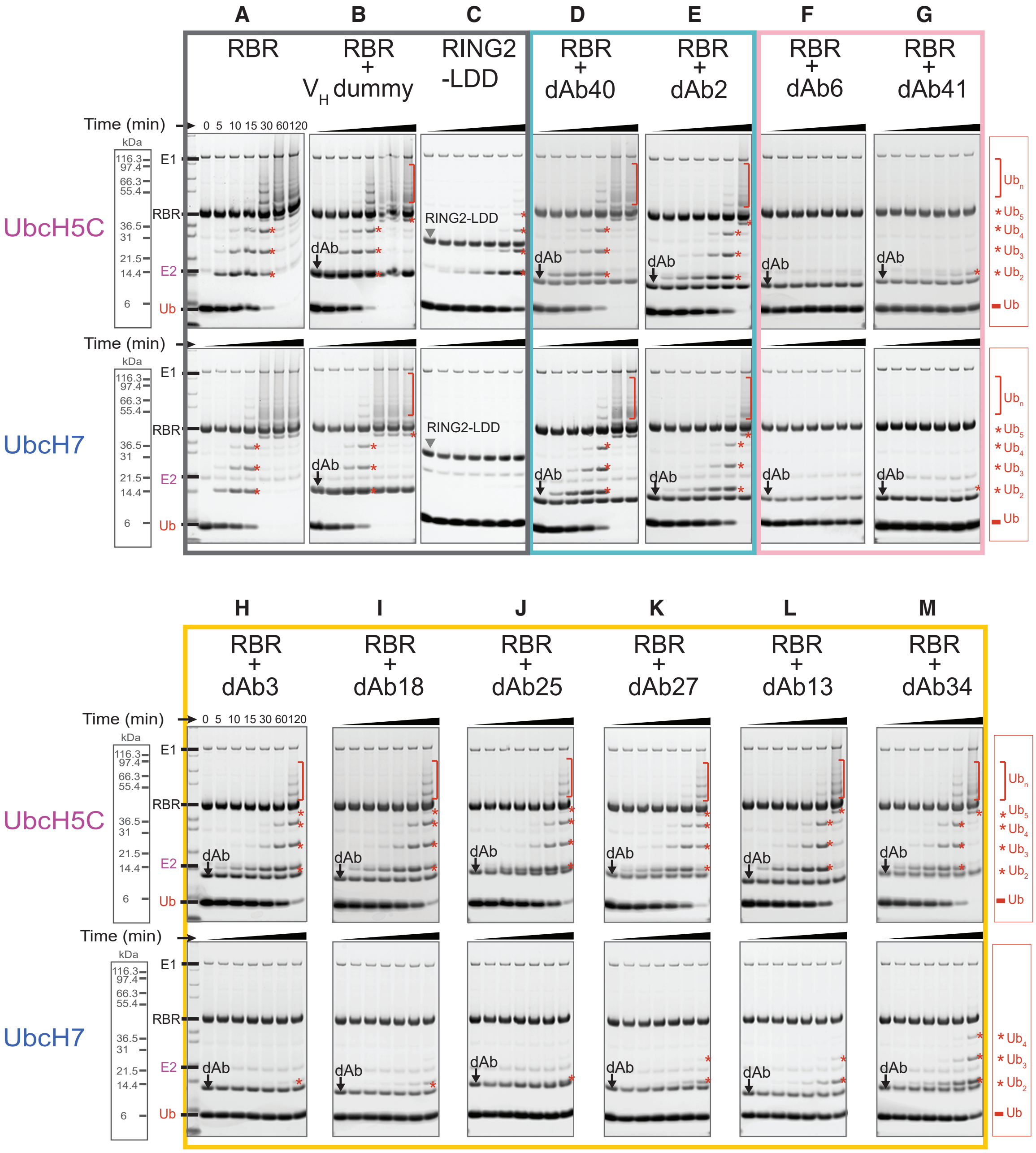

Figure 1. Functional Effects of Select dAbs on HOIP Activity

(A) *In vitro* ubiquitination assays with the RBR domain of HOIP and the E2s UbcH5C and UbcH7. Gels have been stained with Coomassie blue and converted to gray scale.

(B) Ubiquitination assays with a V_H dummy control.

(C) Ubiquitination assays with the RING2-LDD region of HOIP.

(D–M) Ubiquitination assays in the presence of a 3-fold excess of dAbs to assess their effect on catalytic activity.

The gray box around (A)–(C) indicates controls, the blue box around (D) and (E) neutral dAbs, the pink box broadly inhibitory dAbs, and the yellow box differential modulators.

and active with most E3 ligases. To investigate functional effects of the selected dAbs on the activity of HOIP with UbcH7 and UbcH5C, respectively, we carried out *in vitro* ubiquitination assays at an RBR:dAb ratio of 1:3 to ensure complete saturation of HOIP, as we had observed that some dAbs are dimeric in solution. In the absence of dAbs, HOIP RBR performs similarly with UbcH5C or UbcH7 in linear ubiquitin chain formation assays (Figure 1A), and addition of a three times molar excess of a V_H dummy (a control single-dAb) (Ignatovich et al., 2012) had no effect on HOIP activity (Figure 1B).

The ubiquitination assays highlighted that the ten selected dAbs can be divided into three functional groups based on their effect on free linear ubiquitin chain formation (Figure 1): one group containing two dAbs (dAb 40, K_D = 320 nM; and dAb 2, K_D = 7.5 nM) that have only a minor effect on activity with either E2 (Figures 1D and 1E), while another group of two dAbs (dAb6, K_D = 13 nM; and dAb41, K_D = 3.2 nM) (Figures 1F and 1G) inhibited most, if not all, linear chain formation with both E2s equally. However, six dAbs (dAb3, dAb18, dAb25, dAb27, dAb13, and dAb34) behave differently depending on the E2 used: they have a small effect on the observed activity with UbcH5C, but they drastically slow down linear chain formation with UbcH7 (Figures 1H–1M). This difference in activity is reminiscent of the behavior of the isolated HOIP RING2-LDD construct, which is inactive with UbcH7 but retains some activity with UbcH5C (Figure 1C).

Those dAbs that only had a minor effect on catalytic activity with either E2 enzyme, were further examined on SEC-MALLS to investigate the stoichiometry of complex formation and ensure that the RBR domain had been fully saturated in the functional assays. These experiments demonstrated that dAb2 and dAb40 both form a 1:1 complex with HOIP RBR, as does dAb34, a weaker binder (K_D = 1.7 μM) (Figures S2A–S2C). To gain a molecular understanding of these functional effects of different dAbs, we employed hydrogen-deuterium exchange coupled to mass spectrometry (HDX-MS) to identify HOIP epitopes of the selected dAbs.

Mapping HOIP Epitopes by HDX-MS

HDX-MS is useful for monitoring the exchange of peptide backbone amide protons. The technique was used here to map changes in solvent accessibility and hydrogen bonding in HOIP RBR upon dAb complexation, as determined by differential rates of deuterium incorporation of pepsin-derived peptides from HOIP. In simple cases, binding epitopes are revealed by appearance of "protected" patches of surface amides that exchange more slowly as they are shielded from solvent (and potentially form new H-bonds) on antibody binding. Interestingly, this was not what we saw. Firstly, the pattern of changes we observed was varied among the dAbs, suggesting we had identified a range of binding sites and binding modes. Secondly, antibodies induced not only regions of HDX-MS protection, but also showed significant regions of deprotection, suggesting that some dAbs may lock HOIP in a more open conformation. To more easily visualize and compare the behavior of these dAbs, the differential deuteration (as a proportion of the maximum deuteration) was plotted versus the pepsin-derived HOIP peptides for each dAb complex and clustered using a Euclidean distance approach (Figure 2A).

Although the proposed classification does not give an absolute correlation with functional inhibition, the clustering does allow us to better speculate the structural basis for the observed effects on activity. Class 1–3 dAb complexes display the classic protection behavior. Class 1 and class 2 antibodies (dAb40, dAb18, and dAb41) all strongly reduce amide exchange in the linker connecting RING1 and IBR, here referred to as the helix-turn-helix (HTH) region of HOIP, suggesting that this is likely to be an interaction site for all these proteins (Figures 2B–2D). However, as the protection pattern extends more toward IBR and RING2 for dAb18 and dAb41, respectively, we see an increase in these antibodies' ability to inhibit ubiquitination. This is consistent with these antibodies encroaching on firstly, the donor ubiquitin (Ub_{donor}) site on IBR, and, secondly, sterically interfering with the domain movement necessary for catalysis (Figures 2C and 2D). The exchange patterns for most members of class 3 (dAb27, dAb6, and dAb13) are similar to dAb41 in retaining their interaction with IBR and the linker leading to RING2, but lose the protection in the HTH region. The pattern of protection among the class 3 dAbs suggests that binding to IBR can inhibit UbcH7/RBR activity, but to inhibit both UbcH5C/RBR and UbcH7/RBR actions the binding epitope may need to extend to regions close to C885 in RING2, consistent with dAb41 and dAb6 being the only two dAbs with this activity profile. The lack of inhibitor activity of dAb2 may be due to binding to the IBR but oriented away from surfaces required for ubiquitin transfer (Figure S3).

The three antibodies in class 4 (dAb3, dAb25, and dAb34) have non-classical protection patterns with a very small region of protection in IBR and large regions of deprotection in RING1, HTH, and RING2, especially for dAb25 and dAb34. We hypothesize that these dAbs may bind to IBR of HOIP stabilizing a distinct conformation, more open than its average solution conformation, such that many of the backbone amides are more solvent exposed than normal.

High-Resolution Crystal Structure of HOIP RBR in Complex with dAb3

To test the suitability of the selected dAbs to act as crystallization chaperones, ten HOIP RBR/dAb complexes were prepared at a 1:3 ratio, further purified by size-exclusion chromatography and screened in crystallization trials. The RBR/dAb3 complex yielded three-dimensional crystals under two different conditions (see the STAR Methods), one of which contained only dAb3. Apo dAb3 crystallized as a dimer and its structure, which was solved by molecular replacement, revealed that the dimer interface is similar to the interface between V_H and V_K of a conventional antibody (Figure S4A). The RBR/dAb3 complex structure was solved by a combination of molecular replacement and anomalous scattering at 2.25 Å (Figure 3A). In the co-crystal structure, the RBR molecule is in an extended conformation, bound to a dAb3 dimer. To investigate if the oligomeric states seen in the crystal structures also occur in solution, apo dAb3, HOIP RBR and the HOIP RBR/dAb3 complexes were evaluated by SEC-MALLS (Figures S2D–S2F). The estimated molecular weights clearly show that dAb3 is dimeric in solution and binds the HOIP RBR as a dimer.

The dAb3 dimer primarily contacts the IBR domain of HOIP, and the key dAb3 residues contributing to complex formation are all from complementarity determining regions (CDRs) (Figure 3B). Interestingly, the front (red) and back (blue) dAb3 molecules contribute different residues toward interactions with the IBR domain of HOIP, and share 898 and 656 Å of surface buried area, respectively. One dAb3 (red) forms hydrogen bonds between S52, P53, I54, T56, Y57 (from CDR2), R103, and G104 (from CDR3), and R792, D793, C802, and S803 of HOIP (Figures 3B–3D). Additional contacts contributed by the other dAb3 (blue) include S33 from CDR1, T50, and Y59 from CDR2, and S100, Y101, S102, R103, and T105 from CDR3, which form hydrogen

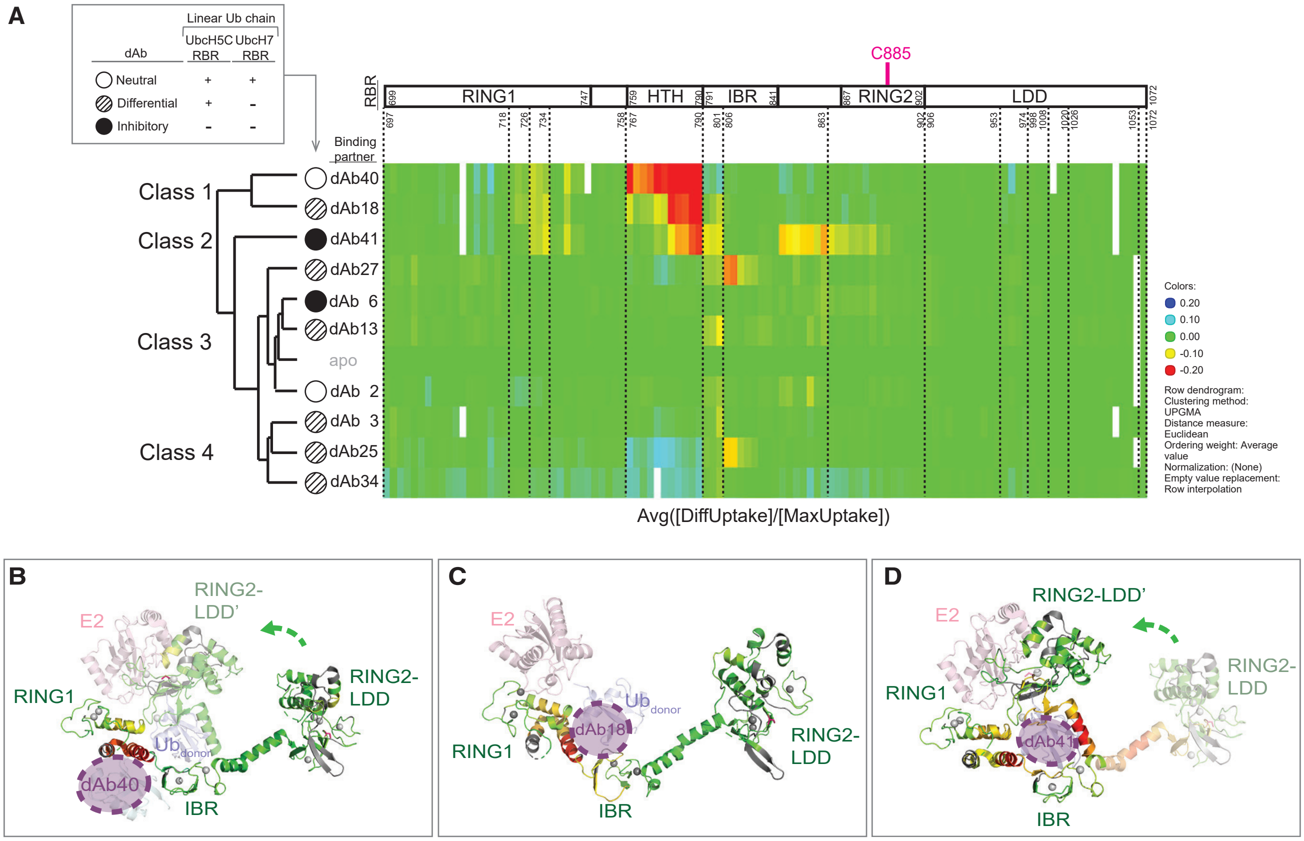

Figure 2. HDX-MS Analysis of HOIP/dAb Complexes

(A) Plot of differential deuteration versus HOIP peptides for each dAb complex. Four classes are presented according to clustering based on Euclidean distance approach. The functional effect of each dAb is symbolized as follows: white circle indicates neutral effect, circle with diagonal fill indicates differential effect, black circle indicates inhibitory effect.

(B–D) Differential deuteration heatmaps displayed on the structure of HOIP RBR in complex with a UbcH5B-Ub conjugate (PDB: 5EDV). The elongated HOIP RBR molecule found in the crystal structure is indicated by RING2-LDD, whereas the closed form that is suggested to bind UbcH5B-Ub is indicated by RING2-LDD′ (Lechtenberg et al., 2016). The position of catalytic C885 located in RING2 is indicated in magenta stick representation. Structures are colored according to the deuteration heatmap shown in (A), gray indicates regions for which no peptides were recovered. (B) Example of a neutral dAb (dAb40) binding to a region of HOIP that has been suggested to bind an allosteric ubiquitin molecule (Lechtenberg et al., 2016). (C) Example of a differential dAb (dAb18) contacting a region close to the IBR domain that overlaps with the binding site of the donor ubiquitin. (D) Example of an inhibitory dAb (dAb41), which may trap the RBR domain in a conformation where the catalytic cysteine C885 is occluded.

bonds with Q801, S803, Q822, R827, and K829 (Figures 3B, 3E, and 3F). Therefore, each dAb3 molecule has a unique interface with different sections of the IBR.

In the HOIP/dAb3 complex the RBR domain is trapped by dAb3 in an extended conformation. The dimension of the extended molecule is similar to the RBR monomer observed in complex with UbcH5B-Ub (Lechtenberg et al., 2016), but with a different linker torsion between RING1 and IBR. Overlaying the two RBR structures via the IBR domain shows that RING1 and RING2 of RBR^{dAb3} and $RBR^{UbcH5B-Ub}$ are tilted at different angles with respect to the IBR (Figure 4A). This is the result of linker twisting and highlights a degree of flexibility and movement in the linker regions that is likely important for ligase function.

In the complex structure described here, the E2 binding interface on RING1 is available and access to the active site cysteine in RING2 is not occluded (Figure 4B). However, dAb3 occupies the position where the donor ubiquitin would be located (Figure S5A), explaining why binding of the dAb affects catalytic activity. Nevertheless, while ubiquitination assays show that binding of dAb3 completely abolishes activity with UbcH7, activity with UbcH5C is only reduced, reminiscent of the behavior of a RING2-LDD construct (Figures 1C and 1H). All activity detected in ubiquitination assays depends on the presence of HOIP and no background ubiquitination from E2 is observed in its absence (Figure S5B).

RBR/dAb3 Crystals Enable Small-Molecule Soaking and Structure-Based Ligand Design

We recently identified Cys-reactive HOIP inhibitor (**1**) through fragment-based covalent ligand screening and reported a ligand-bound RING2-LDD structure solved at 2.15 Å resolution (referred to as compound (**5**) in [Johansson et al., 2019]). However, crystal formation required rounds of seeding, crystal morphology was delicate, and diffraction was anisotropic. Similar challenges were reported for the HOIP RBR/UbcH5B-Ub crystals, which diffracted only to medium resolution,

A

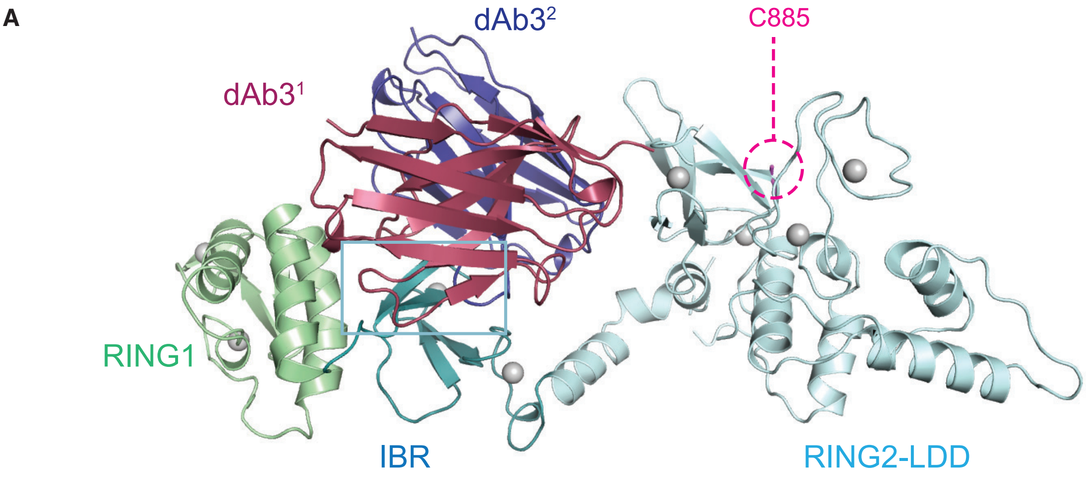

B

	C & D		E & F	
CDR	dAb3¹ residues	HOIP RBR	dAb3² residues	HOIP RBR
1	G31		G31	
	Y32		Y32	
	S33		S33	Q801
	M34		M34	
	A35		A35	
2	T50		T50	S803
	I51		I51	
	S52	D793	S52	
	P53	D793	P53	
	I54	D793	I54	
	G55		G55	
	T56	D793	T56	
	Y57	R792	Y57	
		D793		
	T58		T58	
	Y59		Y59	Q822
	Y60		Y60	
	A61		A61	
	D62		D62	
	S63		S63	
	V64		V64	
	K65		K65	
	G66		G66	
3	G99		G99	
	S100		S100	K829
	Y101		Y101	Q801
	S102		S102	K829
	R103	C802	R103	R827
	G104	S803	G104	
	T105		T105	K829
	P106		P106	
	F107		F107	
	D108		D108	
	Y109		Y109	

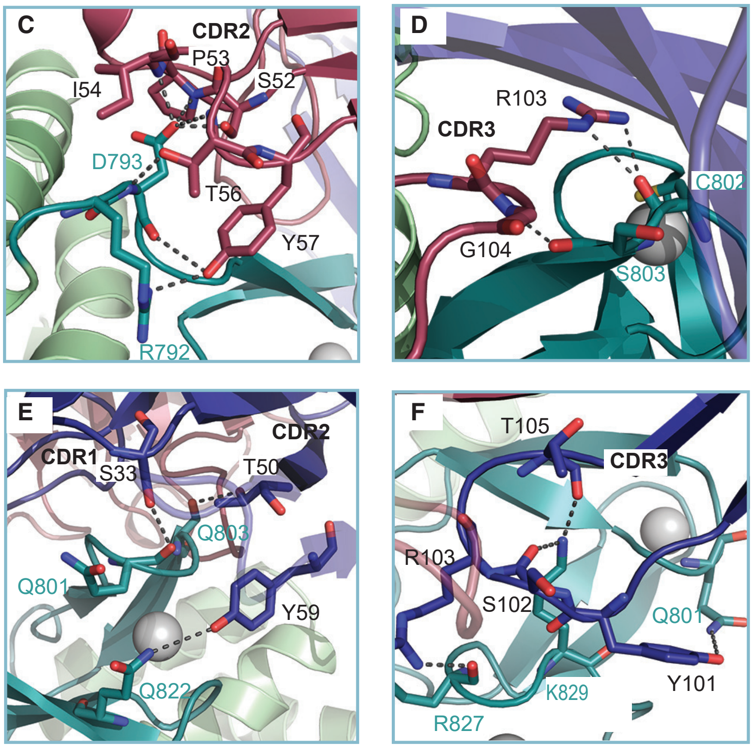

Figure 3. Structure of a HOIP RBR/dAb Complex

(A) X-ray structure of the HOIP RBR-dAb3 complex with HOIP shown in green (RING1), teal (IBR), and cyan (RING2-LDD), and the two monomers of dAb3 in red and blue, respectively. Zn^{2+} ions are shown as gray spheres and the catalytic C885 in RING2, which is accessible is indicated.

(B) Details of the interface between HOIP and dAb3. The sequences of CDR1, CDR2, and CDR3 and residues making contacts are listed.

(C) Interface of CDR2 of dAb3 (in red) and HOIP.

(D) Interface of CDR3 of dAb3 (in red) and HOIP.

(E) Interface of CDR1 and CDR2 of dAb3 (in blue) and HOIP.

(F) Interface of CDR3 of dAb3 (in blue) and HOIP.

exhibited inhomogeneity, and required extensive dehydration procedures (Lechtenberg et al., 2016). Thus, neither approach is suitable for reliable crystallization of HOIP to enable structure-based inhibitor design. Therefore, we envisioned that the HOIP RBR/dAb3 complex could provide a reproducible route to high-quality crystals for ligand soaking. To test the utility of HOIP RBR/dAb3 crystals for ligand soaking we sought to obtain co-crystal structures with covalent inhibitors bearing different scaffolds and a range of molecular complexity. In addition to recently identified inhibitor (**1**), which labels the active site C885 of HOIP via the α,β-unsaturated ester, we showed that analogs (**2**) and (**3**) represent valuable small-molecule chemical

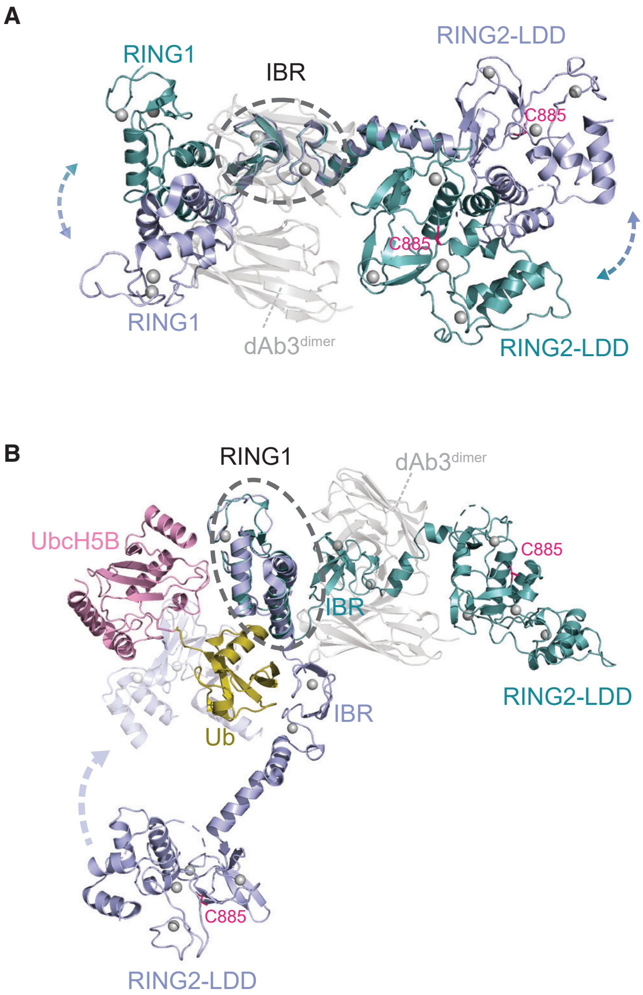

Figure 4. Structural Comparison of Different HOIP Conformations

(A) Structural comparison of the HOIP RBR domain from the dAb3 complex structure (in teal) with one of the RBR monomers from the HOIP RBR/UbcH5B-Ub structure (PDB: 5EDV, in blue), overlapped via the IBR domain. The dAb3 dimer is shown in transparent gray. The catalytic cysteine is shown in magenta as sticks and Zn^{2+} ions are shown as gray spheres.

(B) Structural comparison of the HOIP RBR domain from the dAb3 complex structure (in teal) with one of the RBR monomers from the HOIP RBR/UbcH5B-Ub structure (PDB: 5EDV, in blue), overlapped via the RING1 domain. The UbcH5B-Ub conjugate bound to RING1 is shown in pink and yellow. The catalytic cysteine is shown in magenta as sticks and Zn^{2+} ions are shown as gray spheres.

tools to target HOIP in cells (Figures 5A and 5B) (Johansson et al., 2019). Compared to compound (**2**), compound (**3**) is extended at the ester and bears a *trans*-cyclooctene (TCO) moiety. Despite the significant increase in molecular size and shape, compound (**3**) displays an increased labeling rate of HOIP compared with inhibitor (**2**), and efficiently engages in TCO-tetrazine click chemistry (Johansson et al., 2019). To further investigate the contribution of the ester substituent on inhibitor activity we synthesized analog (**4**) as an intermediate between compounds (**2**) and (**3**) (Scheme S1). Finally, the recently reported α,β-unsaturated ketone HOIP inhibitor HOIPIN-8 (**5**), which is based on a significantly different molecular scaffold (Scheme S2), was synthesized and included in our soaking experiments (Katsuya et al., 2019).

Ligand-bound HOIP RBR/dAb3 structures were solved by molecular replacement at 2.1–2.56 Å (Table S1) with clear electron density for the linkage between C885 and the beta carbon of the unsaturated ester (Figures 5C–5E). Inhibitor (**1**) (Figure 5B) and analogs (**2**)–(**4**) are all based on a bicyclic scaffold containing a pyridone heterocycle and are accommodated in the active site in a highly similar manner. Inhibitors (**1**)–(**4**) all engage HOIP via hydrogen bonds to the backbone of H889 and H887, as well as through aromatic interactions with F888 (Figures 5B–5E). In addition, compounds (**2**)–(**4**) engage in H-bonding with the side chain of H889, which adopts a different conformation in the HOIP RBR/dAb3 complex compared with the HOIP RING2-LDD structure (Johansson et al., 2019). This additional interaction may account for the small increase in labeling rates observed for (**2**) compared with compound (**1**) (Johansson et al., 2019). The structures of HOIP RBR/dAb3 in covalent complex with inhibitors (**3**) and (**4**) also shed some light on the role of the extended ester substituent. The TCO-linked ester extension of compound (**3**) adopts two different conformations in the complex, highlighting a significant flexibility for this part of the molecule. Furthermore, removing the majority of this ester extension in compound (**4**) maintains labeling activity at HOIP RBR (Figure S6), indicating that the extension does not contribute to molecular recognition by HOIP and instead protrudes toward the protein surface and hence is available for TCO-tetrazine click chemistry. Consequently, the observed increased labeling activity observed for inhibitors (**3**) and (**4**) compared with compound (**2**) is likely attributable to increased electrophilicity of the α,β-unsaturated ester.

To further illustrate the utility of our crystallization platform, we also soaked HOIP RBR/dAb3 crystals with the recently reported LUBAC inhibitor HOIPIN-8 (**5**) (Katsuya et al., 2019) to provide a co-crystal structure of compound (**5**) with HOIP. The structure reveals a very different binding mode of (**5**) compared with the pyridone derivatives (**1**)–(**4**). Compound (**5**) sits on a ledge on the surface opposite to the active site C885 and is engaged in a number of specific interactions with HOIP, most notably a salt bridge between the ligand carboxylate and R935 in HOIP and hydrogen bonds between the ligand pyrazole group and D936 (Figure 5F).

Together these structures demonstrate that HOIP RBR/dAb3 crystals provide a straightforward and reproducible platform to access high-resolution structures of HOIP in covalent complex with inhibitors of different molecular scaffolds and complexity (Figures 5G and 5H). The approach constitutes a powerful tool to enable structure-based inhibitor development for HOIP and might be generally applicable to other RBR E3 ligases.

DISCUSSION

Single-dAbs have proven to be powerful research tools for structural and functional studies. Very often they are derived from heavy-chain antibodies of cameloids. Here, we set out to test if dAb libraries based on human scaffolds could be used as tools to gain insight into HOIP E3 ligase function and facilitate structural studies. Using non-immune synthetic libraries, we have successfully obtained tight nanomolar dAb binders to the HOIP

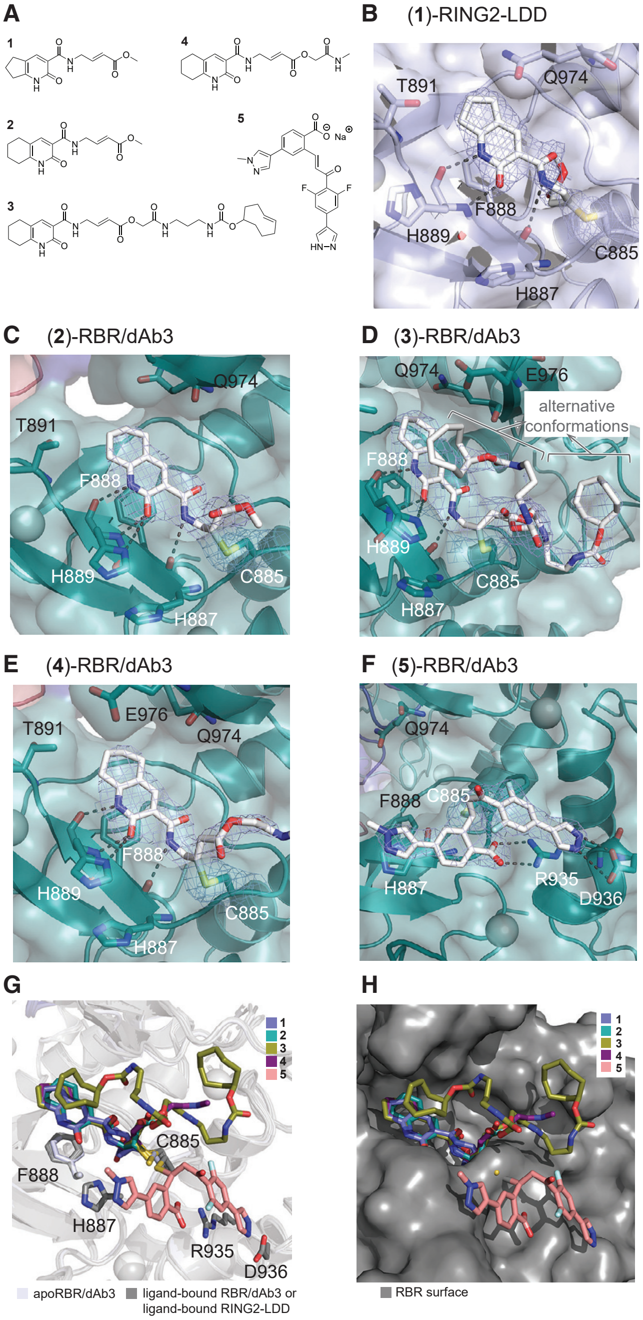

Figure 5. Structural Analysis of Covalent Fragment-HOIP/dAb Complexes

(A) Structure of compounds.

(B) Covalent complex of compound (**1**)-RING2-LDD. Electron density ($2F_o$–F_c) map of the compound is presented in a blue mesh and contoured at 1.2σ.

(C–F) Covalent complexes of compounds (**2**), (**3**), (**4**), and (**5**) with RBR/dAb3. Electron density ($2F_o - F_c$) map of compound is presented in a blue mesh and contoured at 0.8σ for compound (**2**), 0.1σ for compound (**3**), 1.0σ for compound (**4**), and 1.6σ for compound (**5**). Density for the TCO moiety of compound (**3**) is very weak and was modeled as two alternative conformations.

(G) Overlap of all fragment-bound structures with fragments shown in sticks and HOIP shown in ribbons.

(H) Overlap of all fragment-bound structures with fragments shown in sticks and HOIP shown in surface representation.

RBR that have different functional effects on HOIP RBR activity. Although some dAbs affect the activity with the cysteine-only reactive E2 UbcH7 and the broad-specificity E2 UbcH5C to a similar extent, most have a significantly stronger inhibitory effect on the activity with UbcH7. We employed HDX-MS analyses to identify the surfaces of HOIP RBR that are contacted by the ten selected dAbs. Those dAbs that had only a minor effect on linear chain formation, either did not allow the identification of a clear epitope (dAb2), or likely contact a surface of the linker connecting RING1 and IBR that does not interfere with the domain movements required for ubiquitin transfer (dAb40). In contrast, dAb41, which inhibits activity with both E2s likely interferes with the positioning of the donor ubiquitin and possibly obstructs access to the active site cysteine in RING2. Interestingly, all dAbs that show E2-dependent modulation of catalytic activity, contact the IBR domain, and in some cases adjacent linkers, and thereby may interfere with the positioning of the donor ubiquitin during transthiolation from E2 to E3. This differential functional behavior is most likely linked to the dynamic properties of E2-Ub conjugates, which exist in a conformational equilibrium between open and closed states, and to the mechanism by which they recognize the RBR domain. Canonical RING domains stabilize a closed state of the E2-Ub conjugate to activate the thioester bond for ubiquitin transfer onto lysine residues (Dou et al., 2012; Plechanovova et al., 2012; Pruneda et al., 2011, 2012). In contrast, RING1 domains actively prevent formation of a closed E2-Ub conformation and instead promote an open state to suppress non-functional ubiquitin discharge onto a lysine residue and instead allow formation of the E3-Ub intermediate (Dove et al., 2016, 2017; Pruneda et al., 2012; Yuan et al., 2017). Activity with UbcH5C is reduced but not fully abolished, likely because UbcH5-Ub conjugates exist predominantly in open conformations, in which ubiquitin is able to make transient contacts with RING2 and the preceding linker to promote transthiolation, although at a reduced rate (Dove et al., 2016, 2017). In contrast, UbcH7-Ub conjugates preferentially occupy closed states, in which the hydrophobic surface of ubiquitin that is likely to be recognized by RING2 is occluded. Thus, UbcH7-Ub conjugates require binding to RING1 to stabilize an open conformation and promote ubiquitin transfer in *cis*.

Protein ubiquitination by RBR ligases is a multi-step process involving the three subdomains of the RBR motif to transfer ubiquitin from the E2-Ub conjugate to the catalytic cysteine, and finally onto the protein substrate. The linkers connecting the subdomains are flexible, allowing them to undergo the conformational changes necessary to transfer ubiquitin along the reaction pathway. However, such flexibility can make crystallization challenging, and no structures of isolated RBR domains are available at present. Instead, all known crystal structures of RBR ligases are either in an auto-inhibitory form, where domains outside the RBR such as the Ubl and R0 domains in Parkin (Kumar et al., 2015; Riley et al., 2013; Trempe et al., 2013; Wauer and Komander, 2013) or the Ariadne domain in HHARI (Duda et al., 2013) contact the RBR and thereby stabilize otherwise flexible linkers, or they are in complex with functional molecules such as E2-Ub conjugates (Dove et al., 2017; Lechtenberg et al., 2016; Sauve et al., 2018; Yuan et al., 2017) or phospho-ubiquitin for Parkin (Gladkova et al., 2018; Kumar et al., 2017; Wauer et al., 2015). Here we tested if dAbs based on human scaffolds could

be used to stabilize these flexible regions and aid crystallization. Of the ten dAbs selected, dAb3 readily produced crystals in the apo and RBR-bound form. Interestingly, the dAb is dimeric in the apo form and bound to HOIP, with dimerization occurring via an interface similar to that observed between V_H and V_K domains of a conventional antibody, highlighting that synthetic dAbs based on human scaffolds may act as monomers or dimers. Crucially, binding of the dAb enables ready access to stable and reproducible crystals of the HOIP RBR domain that maintain full access to the active site cysteine and therefore are amenable to soaking of small-molecule compounds. Using this approach allowed us to evaluate the mode of binding of compounds based on a pyridone scaffold that had been identified in a covalent fragment-based screen and of a recently reported HOIP inhibitor HOIPIN-8 for which no binding information was available.

Taken together, our study shows that single-dAbs against an RBR E3 ubiquitin ligase are powerful new tools to provide insight into the mechanism of ubiquitin transfer by this ligase family and act as crystallization chaperones to provide easy access to ligand-bound structures to aid inhibitor development.

SIGNIFICANCE

E3 ligases are the key regulators of protein ubiquitination as they select the substrate to be modified, and in some cases can determine what type of ubiquitin chain will be formed. Defects in the ubiquitin system are associated with many diseases and there is a lot of interest in drug discovery to target components of this system. However, progress in developing small molecules that could be used as tool compounds or therapeutics has been slow. Structure-guided drug design is a powerful approach to develop and improve small-molecule compounds, yet access to high-resolution structures of RBR family E3 ligases has been limited. Here, we describe the selection and characterization of single-domain antibodies against the LUBAC subunit HOIP, which provide access to stable and reproducible crystals of the HOIP RBR domain that can be used for soaking of small-molecule inhibitors. Considering the importance of LUBAC in the regulation of immune and apoptotic signaling pathways, these dAbs will help accelerate development of efficient and specific inhibitors of signaling pathways regulated by linear ubiquitin chains.

STAR★METHODS

Detailed methods are provided in the online version of this paper and include the following:

- KEY RESOURCES TABLE
- LEAD CONTACT AND MATERIALS AVAILABILITY
- EXPERIMENTAL MODEL AND SUBJECT DETAILS
- METHOD DETAILS
  - Recombinant Protein Constructs and Purification
  - Biotinylation of HOIP RBR Constructs
  - Domain Antibody Selection
  - Colony PCR and Phage ELISA
  - Soluble dAb Construction
  - Soluble dAb Purification
  - Differential Scanning Fluorimetry
  - Biolayer Interferometry
  - SEC-MALLS
  - Auto-Ubiquitination Assays
  - Hydrogen-Deuterium Exchange Mass Spectrometry (HDX-MS)
  - Protein Crystallization
  - Ligand Soaking
  - Data Collection and Processing
  - General Experimental Information for Compound Synthesis
  - Compound Synthesis
  - Protein LCMS Assay
- QUANTIFICATION AND STATISTICAL ANALYSIS
  - Determination of Dissociation Constant (K_D) Values by Bio-Layer Interferometry (BLI)
- DATA AND CODE AVAILABILITY

SUPPLEMENTAL INFORMATION

Supplemental Information can be found online at https://doi.org/10.1016/j.chembiol.2019.11.007.

ACKNOWLEDGMENTS

We thank Ian Taylor for help with SEC-MALLS experiments, Andy Purkiss for help with X-ray data collection and crystallographic analysis, Philip Drew and Daniel Badcock for guidance and assistance on dAb selection, Nadia Tournier and Trevor Wattam for guidance and assistance on large-scale Octet experiment and data processing, Steve Howell for mass spectrometry analysis and Vangelis Christodoulou for assistance on biotinylation. We acknowledge Diamond Light Source for time on beamlines IO3, IO4, and IO4-1 under proposals MX18566 and MX13775. This work was supported by the Francis Crick Institute which receives its core funding from Cancer Research UK (FC001142 to K.R.), the UK Medical Research Council (FC001142 to K.R.) and the Wellcome Trust (FC001142 to K.R.). We also thank GSK for its commitment to support fundamental discovery research through the establishment of the Crick-GSK Biomedical LinkLabs partnership.

AUTHOR CONTRIBUTIONS

Y.-C.I.T. carried out dAb selection, and designed, ran, and analyzed biochemical and biophysical experiments and crystallographic studies. H.J. synthesized reported compounds and analyzed their reactivity. D.D. and C.-w.C. ran and analyzed HDX-MS studies. S.M. analyzed Octet studies. J.C. oversaw dAb selection. D.H. initiated the project and oversaw compound synthesis and analysis. K.R initiated the project and oversaw all aspects of the project. Y.-C.I.T. and K.R. wrote the manuscript with input from all authors.

DECLARATION OF INTERESTS

D.D., C-w.C., J.C., and D.H. are employees and shareholders of GlaxoSmithKline.

Received: August 7, 2019
Revised: October 15, 2019
Accepted: November 14, 2019
Published: December 5, 2019

REFERENCES

Adams, P.D., Afonine, P.V., Bunkoczi, G., Chen, V.B., Davis, I.W., Echols, N., Headd, J.J., Hung, L.W., Kapral, G.J., Grosse-Kunstleve, R.W., et al. (2010). PHENIX: a comprehensive Python-based system for macromolecular structure solution. Acta Crystallogr. D Biol. Crystallogr. *66*, 213–221.

Berndsen, C.E., and Wolberger, C. (2014). New insights into ubiquitin E3 ligase mechanism. Nat. Struct. Mol. Biol. *21*, 301–307.

Buetow, L., and Huang, D.T. (2016). Structural insights into the catalysis and regulation of E3 ubiquitin ligases. Nat. Rev. Mol. Cell Biol. *17*, 626–642.

Carvalho, A.F., Pinto, M.P., Grou, C.P., Vitorino, R., Domingues, P., Yamao, F., Sa-Miranda, C., and Azevedo, J.E. (2012). High-yield expression in *Escherichia coli* and purification of mouse ubiquitin-activating enzyme E1. Mol. Biotechnol. *51*, 254–261.

Chames, P., Hoogenboom, H.R., and Henderikx, P. (2002). Selection of antibodies against biotinylated antigens. Methods Mol. Biol. *178*, 147–157.

Clabbers, M.T.B., Gruene, T., Parkhurst, J.M., Abrahams, J.P., and Waterman, D.G. (2018). Electron diffraction data processing with DIALS. Acta Crystallogr. D Struct. Biol. *74*, 506–518.

Coomber, D.W. (2002). Panning of antibody phage-display libraries. Standard protocols. Methods Mol. Biol. *178*, 133–145.

Deshaies, R.J., and Joazeiro, C.A. (2009). RING domain E3 ubiquitin ligases. Annu. Rev. Biochem. *78*, 399–434.

Dou, H., Buetow, L., Sibbet, G.J., Cameron, K., and Huang, D.T. (2012). BIRC7-E2 ubiquitin conjugate structure reveals the mechanism of ubiquitin transfer by a RING dimer. Nat. Struct. Mol. Biol. *19*, 876–883.

Dove, K.K., and Klevit, R.E. (2017). RING-Between-RING E3 ligases: emerging themes amid the variations. J. Mol. Biol. *429*, 3363–3375.

Dove, K.K., Olszewski, J.L., Martino, L., Duda, D.M., Wu, X.S., Miller, D.J., Reiter, K.H., Rittinger, K., Schulman, B.A., and Klevit, R.E. (2017). Structural studies of HHARI/UbcH7 approximately Ub reveal unique E2 approximately Ub conformational restriction by RBR RING1. Structure *25*, 890–900.e5.

Dove, K.K., Stieglitz, B., Duncan, E.D., Rittinger, K., and Klevit, R.E. (2016). Molecular insights into RBR E3 ligase ubiquitin transfer mechanisms. EMBO Rep. *17*, 1221–1235.

Duda, D.M., Olszewski, J.L., Schuermann, J.P., Kurinov, I., Miller, D.J., Nourse, A., Alpi, A.F., and Schulman, B.A. (2013). Structure of HHARI, a RING-IBR-RING ubiquitin ligase: autoinhibition of an Ariadne-family E3 and insights into ligation mechanism. Structure *21*, 1030–1041.

Emsley, P., Lohkamp, B., Scott, W.G., and Cowtan, K. (2010). Features and development of coot. Acta Crystallogr. D Biol. Crystallogr. *66*, 486–501.

Enever, C., Coulstock, E., Pupecka-Swider, M., and Hamilton, B. (2014). Single-domain antibodies: an overview. In Handbook of Therapeutic Antibodies, S. Dübel and J.M. Reichert, eds. (Wiley), pp. 311–340.

Gerlach, B., Cordier, S.M., Schmukle, A.C., Emmerich, C.H., Rieser, E., Haas, T.L., Webb, A.I., Rickard, J.A., Anderton, H., Wong, W.W., et al. (2011). Linear ubiquitination prevents inflammation and regulates immune signalling. Nature *471*, 591–596.

Gladkova, C., Maslen, S.L., Skehel, J.M., and Komander, D. (2018). Mechanism of parkin activation by PINK1. Nature *559*, 410–414.

Gottlieb, H.E., Kotlyar, V., and Nudelman, A. (1997). NMR chemical shifts of common laboratory solvents as trace impurities. J. Org. Chem. *62*, 7512–7515.

Hershko, A., and Ciechanover, A. (1998). The ubiquitin system. Annu. Rev. Biochem. *67*, 425–479.

Ignatovich, O., Jespers, L., Tomlinson, I.M., and de Wildt, R.M. (2012). Creation of the large and highly functional synthetic repertoire of human VH and Vkappa domain antibodies. Methods Mol. Biol. *911*, 39–63.

Ikeda, F., Deribe, Y.L., Skanland, S.S., Stieglitz, B., Grabbe, C., Franz-Wachtel, M., van Wijk, S.J., Goswami, P., Nagy, V., Terzic, J., et al. (2011). SHARPIN forms a linear ubiquitin ligase complex regulating NF-kappaB activity and apoptosis. Nature *471*, 637–641.

Jespers, L., Schon, O., James, L.C., Veprintsev, D., and Winter, G. (2004). Crystal structure of HEL4, a soluble, refoldable human V(H) single domain with a germ-line scaffold. J. Mol. Biol. *337*, 893–903.

Johansson, H., Tsai, Y.-C.I., Fantom, K., Chung, C.W., Kumper, S., Martino, L., Thomas, D.A., Eberl, H.C., Muelbaier, M., House, D., et al. (2019). Fragment-based covalent ligand screening enables rapid discovery of inhibitors for the RBR E3 ubiquitin ligase HOIP. J. Am. Chem. Soc. *141*, 2703–2712.

Kabat, E.A., Wu, T.T., Bilofsky, H., Reid-Miller, M., and Perry, H. (1983). Sequences of Proteins of Immunological Interest (U.S. Department of Health and Human Services, Public Health Service, National Institutes of Health).

Katsuya, K., Oikawa, D., Iio, K., Obika, S., Hori, Y., Urashima, T., Ayukawa, K., and Tokunaga, F. (2019). Small-molecule inhibitors of linear ubiquitin chain assembly complex (LUBAC), HOIPINs, suppress NF-kappaB signaling. Biochem. Biophys. Res. Commun. *509*, 700–706.

Kirisako, T., Kamei, K., Murata, S., Kato, M., Fukumoto, H., Kanie, M., Sano, S., Tokunaga, F., Tanaka, K., and Iwai, K. (2006). A ubiquitin ligase complex assembles linear polyubiquitin chains. EMBO J. *25*, 4877–4887.

Komander, D., and Rape, M. (2012). The ubiquitin code. Annu. Rev. Biochem. *81*, 203–229.

Kumar, A., Aguirre, J.D., Condos, T.E., Martinez-Torres, R.J., Chaugule, V.K., Toth, R., Sundaramoorthy, R., Mercier, P., Knebel, A., Spratt, D.E., et al. (2015). Disruption of the autoinhibited state primes the E3 ligase parkin for activation and catalysis. EMBO J. *34*, 2506–2521.

Kumar, A., Chaugule, V.K., Condos, T.E.C., Barber, K.R., Johnson, C., Toth, R., Sundaramoorthy, R., Knebel, A., Shaw, G.S., and Walden, H. (2017). Parkin-phosphoubiquitin complex reveals cryptic ubiquitin-binding site required for RBR ligase activity. Nat. Struct. Mol. Biol. *24*, 475–483.

Lebedev, A.A., Young, P., Isupov, M.N., Moroz, O.V., Vagin, A.A., and Murshudov, G.N. (2012). JLigand: a graphical tool for the CCP4 template-restraint library. Acta Crystallogr. D Biol. Crystallogr. *68*, 431–440.

Lechtenberg, B.C., Rajput, A., Sanishvili, R., Dobaczewska, M.K., Ware, C.F., Mace, P.D., and Riedl, S.J. (2016). Structure of a HOIP/E2-ubiquitin complex reveals RBR E3 ligase mechanism and regulation. Nature *529*, 546–550.

MacKay, C., Carroll, E., Ibrahim, A.F.M., Garg, A., Inman, G.J., Hay, R.T., and Alpi, A.F. (2014). E3 ubiquitin ligase HOIP attenuates apoptotic cell death induced by cisplatin. Cancer Res. *74*, 2246–2257.

Murshudov, G.N., Vagin, A.A., and Dodson, E.J. (1997). Refinement of macromolecular structures by the maximum-likelihood method. Acta Crystallogr. D Biol. Crystallogr. *53*, 240–255.

Plechanovova, A., Jaffray, E.G., Tatham, M.H., Naismith, J.H., and Hay, R.T. (2012). Structure of a RING E3 ligase and ubiquitin-loaded E2 primed for catalysis. Nature *489*, 115–120.

Pruneda, J.N., Littlefield, P.J., Soss, S.E., Nordquist, K.A., Chazin, W.J., Brzovic, P.S., and Klevit, R.E. (2012). Structure of an E3:E2-Ub complex reveals an allosteric mechanism shared among RING/U-box ligases. Mol. Cell *47*, 933–942.

Pruneda, J.N., Stoll, K.E., Bolton, L.J., Brzovic, P.S., and Klevit, R.E. (2011). Ubiquitin in motion: structural studies of the ubiquitin-conjugating enzyme approximately ubiquitin conjugate. Biochemistry *50*, 1624–1633.

Read, R.J., and Schierbeek, A.J. (1988). A phased translation function. J. Appl. Crystallogr. *21*, 490–495.

Riley, B.E., Lougheed, J.C., Callaway, K., Velasquez, M., Brecht, E., Nguyen, L., Shaler, T., Walker, D., Yang, Y., Regnstrom, K., et al. (2013). Structure and function of Parkin E3 ubiquitin ligase reveals aspects of RING and HECT ligases. Nat. Commun. *4*, 1982.

Rittinger, K., and Ikeda, F. (2017). Linear ubiquitin chains: enzymes, mechanisms and biology. Open Biol. *7*, https://doi.org/10.1098/rsob.170026.

Ruiz, E.J., Diefenbacher, M.E., Nelson, J.K., Sancho, R., Pucci, F., Chakraborty, A., Moreno, P., Annibaldi, A., Liccardi, G., Encheva, V., et al. (2019). LUBAC determines chemotherapy resistance in squamous cell lung cancer. J. Exp. Med. *216*, 450–465.

Sakamoto, H., Egashira, S., Saito, N., Kirisako, T., Miller, S., Sasaki, Y., Matsumoto, T., Shimonishi, M., Komatsu, T., Terai, T., et al. (2015). Gliotoxin suppresses NF-kappaB activation by selectively inhibiting linear ubiquitin chain assembly complex (LUBAC). ACS Chem. Biol. *10*, 675–681.

Sauve, V., Sung, G., Soya, N., Kozlov, G., Blaimschein, N., Miotto, L.S., Trempe, J.F., Lukacs, G.L., and Gehring, K. (2018). Mechanism of parkin activation by phosphorylation. Nat. Struct. Mol. Biol. *25*, 623–630.

Schubert, A.F., Gladkova, C., Pardon, E., Wagstaff, J.L., Freund, S.M.V., Steyaert, J., Maslen, S.L., and Komander, D. (2017). Structure of PINK1 in complex with its substrate ubiquitin. Nature *552*, 51–56.

Smit, J.J., Monteferrario, D., Noordermeer, S.M., van Dijk, W.J., van der Reijden, B.A., and Sixma, T.K. (2012). The E3 ligase HOIP specifies linear ubiquitin chain assembly through its RING-IBR-RING domain and the unique LDD extension. EMBO J. *31*, 3833–3844.

Steyaert, J., and Kobilka, B.K. (2011). Nanobody stabilization of G protein-coupled receptor conformational states. Curr. Opin. Struct. Biol. *21*, 567–572.

Stieglitz, B., Morris-Davies, A.C., Koliopoulos, M.G., Christodoulou, E., and Rittinger, K. (2012). LUBAC synthesizes linear ubiquitin chains via a thioester intermediate. EMBO Rep. *13*, 840–846.

Stieglitz, B., Rana, R.R., Koliopoulos, M.G., Morris-Davies, A.C., Schaeffer, V., Christodoulou, E., Howell, S., Brown, N.R., Dikic, I., and Rittinger, K. (2013). Structural basis for ligase-specific conjugation of linear ubiquitin chains by HOIP. Nature *503*, 422–426.

Strickson, S., Campbell, D.G., Emmerich, C.H., Knebel, A., Plater, L., Ritorto, M.S., Shpiro, N., and Cohen, P. (2013). The anti-inflammatory drug BAY 11-7082 suppresses the MyD88-dependent signalling network by targeting the ubiquitin system. Biochem. J. *451*, 427–437.

Ten Eyck, L.F. (1973). Crystallographic fast Fourier transforms. Acta Crystallogr. A *29*, 183–191.

Tokunaga, F., Nakagawa, T., Nakahara, M., Saeki, Y., Taniguchi, M., Sakata, S., Tanaka, K., Nakano, H., and Iwai, K. (2011). SHARPIN is a component of the NF-kappaB-activating linear ubiquitin chain assembly complex. Nature *471*, 633–636.

Tokunaga, F., Sakata, S., Saeki, Y., Satomi, Y., Kirisako, T., Kamei, K., Nakagawa, T., Kato, M., Murata, S., Yamaoka, S., et al. (2009). Involvement of linear polyubiquitylation of NEMO in NF-kappaB activation. Nat. Cell Biol. *11*, 123–132.

Trempe, J.F., Sauve, V., Grenier, K., Seirafi, M., Tang, M.Y., Menade, M., Al-Abdul-Wahid, S., Krett, J., Wong, K., Kozlov, G., et al. (2013). Structure of parkin reveals mechanisms for ubiquitin ligase activation. Science *340*, 1451–1455.

Vonrhein, C., Flensburg, C., Keller, P., Sharff, A., Smart, O., Paciorek, W., Womack, T., and Bricogne, G. (2011). Data processing and analysis with the autoPROC toolbox. Acta Crystallogr. D Biol. Crystallogr. *67*, 293–302.

Walden, H., and Rittinger, K. (2018). RBR ligase-mediated ubiquitin transfer: a tale with many twists and turns. Nat. Struct. Mol. Biol. *25*, 440–445.

Wauer, T., and Komander, D. (2013). Structure of the human Parkin ligase domain in an autoinhibited state. EMBO J. *32*, 2099–2112.

Wauer, T., Simicek, M., Schubert, A., and Komander, D. (2015). Mechanism of phospho-ubiquitin-induced PARKIN activation. Nature *524*, 370–374.

Wenzel, D.M., Lissounov, A., Brzovic, P.S., and Klevit, R.E. (2011). UBCH7 reactivity profile reveals parkin and HHARI to be RING/HECT hybrids. Nature *474*, 105–108.

Winn, M.D., Ballard, C.C., Cowtan, K.D., Dodson, E.J., Emsley, P., Evans, P.R., Keegan, R.M., Krissinel, E.B., Leslie, A.G., McCoy, A., et al. (2011). Overview of the CCP4 suite and current developments. Acta Crystallogr. D Biol. Crystallogr. *67*, 235–242.

Winter, G., Waterman, D.G., Parkhurst, J.M., Brewster, A.S., Gildea, R.J., Gerstel, M., Fuentes-Montero, L., Vollmar, M., Michels-Clark, T., Young, I.D., et al. (2018). DIALS: implementation and evaluation of a new integration package. Acta Crystallogr. D Struct. Biol. *74*, 85–97.

Yau, R., and Rape, M. (2016). The increasing complexity of the ubiquitin code. Nat. Cell Biol. *18*, 579–586.

Yuan, L., Lv, Z., Atkison, J.H., and Olsen, S.K. (2017). Structural insights into the mechanism and E2 specificity of the RBR E3 ubiquitin ligase HHARI. Nat. Commun. *8*, 211.

STAR★METHODS

KEY RESOURCES TABLE

REAGENT or RESOURCE	SOURCE	IDENTIFIER
**Antibodies**		
Mouse anti-M13 phage-HRP conjugate	GE Healthcare	Cat# 27-9421-01; RRID: AB_2616587
**Bacterial and Virus Strains**		
E. coli BL21(DE3) GOLD	Agilent	Cat# 230132
E. coli TG1	Agilent	Cat# 200123
E. coli HB2151	Nordic BioSite	BU-00036
**Chemicals, Peptides, and Recombinant Proteins**		
Compound 2	(Johansson et al., 2019)	N/A
Compound 3	(Johansson et al., 2019)	N/A
Compound 4	This study	N/A
HOIPIN-8 (herein referred to compound 5)	(Katsuya et al., 2019) and this study	N/A
E1	(Carvalho et al., 2012)	N/A
UbcH5C	(Stieglitz et al., 2012)	N/A
UbcH7	(Stieglitz et al., 2012)	N/A
HOIP RBR (residues 697-1072)	(Stieglitz et al., 2012)	N/A
HOIP residues 697-859	This study	N/A
HOIP residues 748-859	This study	N/A
HOIP RING2-LDD	(Stieglitz et al., 2013)	N/A
**Deposited Data**		
Apo dAb3/RBR	This study	PDB: 6SC6
dAb3/RBR-compound 2	This study	PDB: 6SC5
dAb3/RBR-compound 3	This study	PDB: 6SC7
dAb3/RBR-compound 4	This study	PDB: 6SC8
dAb3/RBR-compound 5	This study	PDB: 6SC9
Apo dAb3	This study	PDB: 6T2J
Crystal structure published previously (HOIP RING2-LDD-covalent fragment)	(Johansson et al., 2019)	PDB: 6GZY
Crystal structure published previously (HOIP-RBR/UbcH5B-ubiquitin transfer complex)	(Lechtenberg et al., 2016)	PDB: 5EDV
Crystal structure published previously (HOIP RING2-LDD/ubiquitin transfer complex)	(Stieglitz et al., 2013)	PDB: 4LJO
Crystal structure published previously (Apo HOIP RING2-LDD)	(Stieglitz et al., 2013)	PDB: 4LJQ
Crystal structure published previously (human V_H antibody domain)	(Jespers et al., 2004)	PDB: 1OHQ
**Oligonucleotides**		
Primers for PCR reactions, see Table S2.		
**Recombinant DNA**		
pET156P-TEV-BIRA	MRC PPU (Dundee)	DU43411
**Software and Algorithms**		
CFX Manager version 3.0	Bio-Rad	N/A
Octet Data Acquisition software version 8 & 9	Pall FortéBio	N/A
Octet Data Analysis software version 8	Pall FortéBio	N/A
ASTRA software version 6	Wyatt Technology	N/A
ProteinLynx Global Server v3.0.2	Waters	

(*Continued on next page*)

Continued

REAGENT or RESOURCE	SOURCE	IDENTIFIER
DynamX v3.0	Waters	N/A
Spotfire v7.11	TIBCO Software	N/A
ChemDraw Professional v.17.0.0.206	PerkinElmer	
MestReNova v.12.0.3-21384 software	Mestrelab Research	N/A
MassHunter Workstation Software version B.06.00	Agilent	N/A
autoPROC	(Vonrhein et al., 2011)	N/A
DIALS	(Clabbers et al., 2018; Winter et al., 2018)	N/A
PHENIX	(Adams et al., 2010)	N/A
COOT	(Emsley et al., 2010)	N/A
REFMAC5	(Murshudov et al., 1997)	N/A
JLigand	(Lebedev et al., 2012)	N/A
CCP4 v7.0.076	(Winn et al., 2011)	
PyMOL version 2.3	Schrödinger, LLC	N/A

LEAD CONTACT AND MATERIALS AVAILABILITY

Further information and requests for resources and reagents should be directed to and will be fulfilled by the Lead Contact, Katrin Rittinger (katrin.rittinger@crick.ac.uk) with a completed Materials Transfer Agreement.

EXPERIMENTAL MODEL AND SUBJECT DETAILS

No mammalian cell lines or animal models were used in this study.

METHOD DETAILS

Recombinant Protein Constructs and Purification

Expression and purification of recombinant E1, UbcH5C, UbcH7, HOIP RBR (residues 697-1072), HOIP RING2-LDD (residues 853-1072) have been described previously (Carvalho et al., 2012; Stieglitz et al., 2012, 2013), and are summarised below. HOIP 697-859 (containing RING1 and IBR) and HOIP 748-859 (containing IBR and adjacent linkers) were cloned by Gibson Assembly according to manufacturer's instruction. The BirA expression vector was purchased from the MRC PPU, Dundee (DU43411). All constructs were verified by DNA sequencing. Recombinant proteins with a hexahistidine tag (E1 and BirA) were purified by HisTrap HP column (GE Healthcare) with imidazole elution. E1 was further purified by size exclusion chromatography (SEC) on Superdex 200 (GE Healthcare) in SEC buffer (50 mM HEPES pH 7.5, 150 mM NaCl, 0.5 mM TCEP). Constructs with a GST tag (UbcH5C, UbcH7 and both HOIP constructs) were purified with Glutathione Sepharose 4B or Glutathione Sepharose 4 Fast Flow (GE Healthcare). The GST tag was removed by 3C protease cleavage and proteins were further purified by SEC (Superdex 75 or Superdex 200, GE Healthcare) in SEC buffer. Lyophilised ubiquitin was purchased from Sigma-Aldrich (U6253), dissolved in SEC buffer and purified on Superdex 75 (GE Healthcare). Protein concentrations were determined by UV absorption at 280 nm using their respective extinction coefficients.

Biotinylation of HOIP RBR Constructs

AviTag (peptide GLNDIFEAQKIEWHE) was introduced to C-terminus of HOIP RBR (HOIP residues 697-1072), HOIP 697-859, HOIP 748-859 and HOIP 853-1072 by PCR with primers listed in Table S2.

PCR was carried out using KOD hot start master mix (Novagen) according to manufacturer's instructions. The target fragment was cloned into pET49b (Novagen) by LIC cloning or Gibson Assembly. Biotinylation of HOIP constructs was performed *in vitro* based on an established protocol (Avidity). In short, 100 μL of Biomix-A (0.5 M bicine buffer pH 8.3), 100 μL of Biomix-B (100 mM ATP, 100 mM $MgSO_4$, 0.5 mM d-biotin), 700 μL of 60 μM HOIP RBR, 100 μL of 0.5 mM d-biotin and 20 μL of 1 mM His-BirA were mixed in a micro-centrifuge tube. The reaction was incubated at 20°C with gentle mixing (300 rpm) overnight. Biotinylated HOIP constructs were separated from His-BirA and excess d-biotin by affinity chromatography (HisTrap; GE Healthcare) followed by buffer-exchange into 50 mM HEPES pH 7.5, 150 mM NaCl, 0.5 mM TCEP. Full biotinylation of HOIP constructs was confirmed by Mass Spec.

Domain Antibody Selection

Construction of synthetic human V_H and V_κ domain antibody libraries based on single human framework for V_H (V3-23/DP47 and JH4b) and V_κ (O12/O2/DPK9 and Jκ1) with side chain diversity incorporated at positions in the antigen binding site was described

previously (Ignatovich et al., 2012). In V_H libraries, the combined length of CDR1 and CDR2 is fixed at 22 residues while the CDR3 lengths vary from 7 to 20 residues; in V_κ libraries, the length of CDR1 and CDR2 combined is 18 residues while the CDR3 length is fixed at 9 residues.

Phage selection was based on established methods (Chames et al., 2002; Coomber, 2002). In brief, phage libraries were blocked with 3% milk-PBS for 30 min on a rotor mixer at room temperature (RT). Antigen (biotinylated HOIP RBR) was added to the blocked phage libraries to a final concentration of 100 nM and the mixture was incubated for 1 h on a rotor mixer at RT. Magnetic streptavidin beads (Dynabeads M-280, Invitrogen) were added to the phage libraries/antigen mixture and incubated for 5 min to capture biotinylated RBR and antigen-bound dAb-phage. Eight rounds of washing (PBS supplemented with 0.1% Tween 20 followed by PBS) and elution of phages (0.5 mL PBS supplemented with 1 mg/mL of Trypsin) were carried out with the KingFisher purification system (Thermo Fisher Scientific). Eluted phages (10 μL) were mixed with PBS to create a 10-fold serial dilution from 10^{-1} to 10^{-6}. Phage dilutions (10 μL) were used to infect *E. coli* TG1 strain (90 μL of log-phase culture) at 37°C for 45 min without shaking, followed by spreading 10 μL of infected TG1 culture per library dilutions on a LB agar plate supplemented with 15 μg/mL tetracycline. After overnight incubation at 37°C, the number of eluted phage was determined by: Number of colonies × dilution factor × 10 (volume dilution factor during TG1 transfection) × 50 (to back-calculate eluted phage per 0.5 mL from 10 μL eluted phage used). Outputs of phage libraries were collected by the following procedure: eluted phages (0.25 mL) were incubated with 1.75 mL TG1 log-phase culture at 37°C and the re-suspended pellets were spread on LB agar plates supplemented with tetracycline and incubated at 37°C. After overnight growth, 2 mL of 2X YT media supplemented with 15% glycerol was added to the agar plates. Colonies transfected with phage libraries were collected by a spreader with thorough mixing with the media. Phage libraries were amplified by incubating the re-suspended mixture in 2X YT media supplemented with tetracycline at 37°C, 250 rpm, overnight. After pelleting the cells at 4500 *g* for 30 min at 4°C, the supernatant containing phage was collected while the plasmid DNA was isolated from the cell pellet using QIAfilter Plasmid Maxi kit (Qiagen). Phage was precipitated from supernatant by incubating 40 mL of supernatant with 10 mL of PEG/NaCl (20% PEG 8000, 2.5 M NaCl; Sigma) on ice for 1h. The phage pellet was collected at 4500 *g* for 30 min at 4°C and re-suspended with PBS. The titer of rescued phage was determined by optical density and 10^{10} enriched phage particles were carried to the next round of selection. Two more rounds of selection were executed with the biotinylated HOIP RBR concentration reduced to 20 nM in the second round, and 2 nM in the third round.

Colony PCR and Phage ELISA

Colonies of titer plates from the second and third round of selections were randomly picked and mixed with 25 μL of PCR mix (Platinum Blue PCR Supermix (Invitrogen), 0.2 μM forward primer DOM006 5'-ATGGTTGTTGTCATTGTCGGCGCA-3' and 0.2 μM reverse primer DOM57 5'-ATGAGGTTTTGCTAAACAACTTTC-3'). The PCR reaction was carried out as follows: 1) 96°C, 3 min; 2) 95°C, 45 sec; 3) 55°C, 30 sec; 4) 72°C, 1 min; 5) repeat steps 2-4 for 35 cycles; 6) 72°C, 5 min. Amplified DNA was sequenced and sequence diversity was analysed using Genedata Biologics® (Genedata).

Picked colonies were transferred to 1 mL of 2X YT supplemented with tetracycline and incubated at 37°C, 250 rpm, overnight. Supernatants containing dAb-phage were clarified at 1800 *g* for 10 min at 4°C. Unmodified HOIP RBR was coated onto a 96-well MaxiSorp immunoplate (Nunc) by incubating 50 μL of 5 μg/mL of HOIP RBR per well at 4°C, overnight. Coated HOIP RBR was washed with PBS 3 times before blocking with 2% milk-PBS for 1 h at RT. Wells were washed with PBS 3 times before incubation with the dAb-phage/milk-PBS mixture (50 μL of dAb-phage supernatant and 50 μL of 4% milk-PBS) for 1 h at RT. Wells were washed 3 times with PBST (PBS, 0.1% Tween 20) and once with PBS, followed by incubation with anti-M13 phage-HRP conjugate (GE Healthcare) diluted 1/5000 in 2% milk-PBS for 2 h at RT. Wells were washed with PBST 3 times and PBS once before adding 50 μL of SureBlue 1-Component TMB MicroWell Peroxidase solution (Seracare). The reaction was quenched with 100 μL of 1 M HCl and evaluated by absorbance at 450 nm. Specific binder percentage from the second and third round of selections was calculated to evaluate the enrichment of a given binder.

Soluble dAb Construction

Plasmid DNA isolated after the second and third rounds of selections were digested with SalI/NotI or NcoI/NotI restriction enzyme combinations (New England BioLabs). Host vector pC10 (a vector derived from pUC19) with a PelB leader sequence and C-terminal FLAG-tag for soluble dAb expression was prepared by SalI/NotI digestion or NcoI/NotI digestion (NEB). Cut inserts were ligated to pC10 vector by T4 ligase (NEB). Ligated inserts were transformed into *E. coli* HB2151 (Nordic BioSite; made chemically competent in-house) by heat shock at 42°C for 45 sec. Colonies grown overnight at 37°C on LB agar plates supplemented with 5% glucose and 100 μg/mL carbenicillin were randomly selected. Colony PCR performed with forward primer DM008 (5'-AGCGGATAACAATTTCA CACAGGA-3') and reverse primer DM009 (5'-CGCCAGGGTTTTCCCAGTCACGAC3-3') were used to evaluate the percentage of positive clones containing inserts.

Untagged dAb constructs were cloned by Gibson Assembly according to manufacturer's instruction with the primers listed in Table S2.

Amplified fragments were assembled with pC10 vector and transformed into *E. coli* HB2151 as described above. Sequences of complementarity determining regions (CDRs) of selected dAbs are listed in Table S3 where CDRs are defined by the Kabat numbering scheme (Kabat et al., 1983).

Soluble dAb Purification

Cells were grown in Overnight Express Autoinduction system 1 (Merck) supplemented with 100 μg/mL carbenicillin and foam control agent (Bisomer G30, GEOSC) at 30°C, 250 rpm for 48-72 hr. Supernatants containing soluble dAbs were clarified by centrifugation at 4000 *g* for 60 min at 4°C and applied to MabSelect Xtra resin (GE Healthcare). The resin packed in a gravity column was washed with 55 mM Tris-base, 45 mM acetic acid (pH 7.5) and the sample was applied to the column. The column was washed with 55 mM Tris-base, 45 mM acetic acid, 300 mM sodium acetate, 100 mM sodium octanoate (pH 7.5), followed by buffer containing 55 mM Tris-base, 45 mM acetic acid (pH 7.5). Domain antibodies were eluted in 75 mM acetic acid (pH 3) and were immediately neutralized with 1 M Tris (pH 7.4). Eluted dAbs were concentrated and buffer-exchanged into PBS with a 5 kDa MWCO Vivaspin 20 concentrator. Protein purity was evaluated by SDS-PAGE and quantity was estimated by UV absorption at 280 nm.

Differential Scanning Fluorimetry

Protein stability was assessed using differential scanning fluorimetry. For purified dAb evaluation, samples were prepared at 1 mg/mL. For complex evaluation, HOIP RBR was prepared at 1 mg/mL and mixed with dAbs at a 1:1 molar ratio. Samples were prepared in PBS buffer at 20 μL. Sypro Orange (Sigma Aldrich S5692) was diluted 500-fold in milliQ water at 20 μL and added to protein samples. After mixing the dye and samples in a PCR 96-well plate (Thermo Fisher Scientific AB1400), samples were spun down by centrifugation at 1000 *g* for 2 min. The experiment was started with incubating the samples at 40°C for 1 min, followed by a temperature gradient of 40-95°C with 0.5°C per minute increment (Bio-Rad CFX96 real-time PCR detection system). The data were recorded and reported with CFX Manager version 3.0 (Bio-Rad).

Biolayer Interferometry

For preliminary binder selection, soluble domain antibodies (C-terminally FLAG-tagged) were expressed in 1 mL of Overnight Express Autoinduction system 1 (Merck) supplemented with carbenicillin at 30°C, 850 rpm for 48-72 h in an incubator maintained with 90% humidity. Soluble dAbs were secreted into the medium and supernatants were clarified by filtration (96-well filter plate, Millipore). To immobilise target proteins, biotinylated HOIP RBR or its truncated versions (HOIP 697-859, HOIP 748-859 or HOIP 853-1072) were diluted to 10 μg/mL in PBSF (PBS supplemented with 0.1% IgG-free Bovine Serum Albumin) and incubated with Streptavidin (SA) biosensors (Pall FortéBio Corp., Menlo Park, CA, USA) for 3 min. The sensors were subsequently washed with PBSF for 1 min and incubated with PBSF for an additional minute for baseline stabilization. Association was performed by incubating the sensors with supernatants containing dAbs for 90 sec, followed by dissociation with expression medium for 1 min. Sensors were regenerated with 20 mM NaOH and 0.5% Surfactant P20 (GE Healthcare) and neutralised with PBSF. For further selection and binding validation with purified dAbs, FLAG-tagged dAbs were prepared at 10 μM in PBSF. Immobilization of target proteins (full-length or truncated HOIP RBR) and association of dAbs were performed as described above. Dissociation was performed in PBSF. The sensors were regenerated and neutralised as described above. Both preliminary binder and purified dAb selections were carried out with Octet RED 384 biolayer interferometer (Pall FortéBio) at a constant reaction temperature of 25°C. Data were recorded with Octet Data Acquisition software (version 8) and evaluated with Octet Data Analysis software (version 8) and MS Excel.

To determine the binding affinity based on association and dissociation rate constants, Streptavidin (SA) biosensors were incubated with biotinylated HOIP RBR at 10 μg/mL in PBSF for 3 min for immobilization. The sensors were subsequently washed with PBSF for 1 min and incubated with PBSF for an additional minute for baseline stabilization. Association was followed for up to 5 min by incubating the sensors with purified dAbs prepared at adequate serial dilutions in PBSF. Dissociation was followed for 10 min by incubating the sensors with PBSF. Measurements were carried out with Octet RED 96e (Pall FortéBio) at a constant reaction temperature of 25°C. Data were recorded and exported with Octet Data Acquisition software (version 9). The association phase was analyzed as a double exponential function to account for a small amount of unspecific binding. A plot of the observed rate (k_{obs}) for the major component was linearly dependent on dAb concentration and this gave an association rate constant (k_{on}) from the slope (Table 1 and Figure S1). A value for k_{off} was determined from independent analysis of the dissociation phase and the equilibrium dissociation constant (K_D) was calculated as k_{off}/k_{on} (Table 1).

SEC-MALLS

For an initial evaluation of the soluble state of the dAbs selected, samples were prepared at 1 mg/mL in PBS at 100 μL and applied onto a TSK 2000 column (Tosoh Corporation) equilibrated with 0.1 M Sodium phosphate (monobasic), 0.2 M NaCl, 15% n-propanol, pH 7.4 at a flow rate of 0.5 mL/min at 25°C. Scattered light intensity was recorded using a Wyatt miniDawn Treos multi-angle laser photometer and an Optilab differential refractive index (dRI) detector (Wyatt Technology). Data were evaluated by ASTRA software version 6 (Wyatt Technology).

The oligomeric state of HOIP RBR, dAb3, and HOIP RBR in complex with dAb2, dAb3, dAb34 and dAb40 were evaluated under the following conditions. HOIP RBR was prepared at 1, 2, 4, 8 mg/mL while dAb3 was prepared at 1, 2, 4, 5 mg/mL. Complex samples were prepared at RBR:dAb = 1:1, 1:2, 1:4 and 2:1 molar ratio. Samples were incubated on ice for 30 min and filtered with centrifugal filters (Ultrafree-MC 0.22 μm pore size, Millipore) before analysis. Samples (100 μL) were applied to a Superdex 75 or Superdex 200 10/300 GL column (GE Healthcare) equilibrated with 50 mM HEPES pH 7.5, 150 mM NaCl, 3 mM NaN_3 at a flow rate of 1 mL/min at 25°C. The scattered light intensity was recorded using a Wyatt DAWN-HELEOS II laser photometer and an Optilab differential refractive index (dRI) detector (Wyatt Technology). The data were evaluated with ASTRA software version 6 (Wyatt Technology).

Auto-Ubiquitination Assays

Assays were adapted from an established protocol (Stieglitz et al., 2012). Domain antibodies (15 μM) were incubated with HOIP RBR (5 μM) at three times molar ratio for 30 min on ice before mixing with reaction mixture containing: E1 (0.5 μM), E2 (UbcH5C or UbcH7, 1.25 μM), ubiquitin (40 μM), ATP (10 mM), in 50 mM HEPES pH 7.5, 150 mM NaCl, 20 mM $MgCl_2$. In control experiments, the dAb/RBR mixture was replaced by 5 μM of HOIP RBR or 5 μM of HOIP RING2-LDD. Total reaction volume was 100 μL and the reaction was carried out at 25°C. Aliquots were taken at specified time points: 0, 5, 10, 15, 30, 60 and 120 min. Samples were mixed with 4X NuPAGE LDS sample buffer (Invitrogen) and heated for 10 min at 95°C. Samples were separated on SDS-PAGE using NuPAGE MES-SDS running buffer (Invitrogen) and gels were stained with Quick Coomassie Stain (Generon).

Hydrogen-Deuterium Exchange Mass Spectrometry (HDX-MS)

Apo-HOIP RBR was prepared at 15 μM in dilution buffer (50 mM sodium phosphate, 100 mM NaCl, pH 7.0). Mixtures of HOIP RBR and dAb were prepared at 15 μM HOIP RBR and the following molar ratios to saturate the binding based on available affinity data: dAbs 2, 6, 13, 25 & 27 were mixed 2(dAb):1(HOIP RBR); other dAbs were mixed 3(dAb):1(HOIP RBR).

Sample handling and mixing were performed using a LEAP H/D-X PAL robot (LEAP Technologies, Carrboro, NC, USA) and liquid chromatography was performed using an Acquity M class UPLC (Waters, Manchester, UK). Samples were subjected to a standard deuteration method using 20-fold dilution into deuteration buffer (50 mM sodium phosphate, 100 mM NaCl in D_2O, pD 7.0). Duplicate samples were run for deuteration periods of 0, 0.5 and 5 min at 20°C, followed by quenching by addition of an equal volume of quench buffer (400 mM potassium phosphate, 6 M guanidine hydrochloride, 0.5 M TCEP, pH 2.5). After a 1 min quench samples were then injected at 90 μl/min onto a protease column at 15°C (Waters Enzymate BEH pepsin column; 2.1 × 30 mm). The column was washed with 0.2% formic acid for 4 min, with eluted peptides captured on a Waters BEH C18 VanGuard Pre-column (2.1 × 5 mm). This was then switched in-line with a BEH C18 analytical column (1.0 × 100 mm; 1.7 μm particles, 130 Å pore size) and peptides eluted with a linear 8 min gradient from 12% B to 36% B at 40 μl/min followed by a further 1 min gradient to 95% B (HPLC solvents were: A = 0.2% formic acid + 0.03% TFA in water; B = acetonitrile + 0.2% formic acid). Chromatography was performed at 0°C. During chromatography the pepsin column was washed twice with 80 μl injections of 2 M guanidine hydrochloride, 0.8% formic acid, 5% acetonitrile, 5% propan-2-ol, pH 2.5. The C18 column eluate was analysed by a Waters Synapt G2-Si mass spectrometer using positive-mode electrospray ionization and TOF detection operating in resolution mode from *m/z* 250 to *m/z* 2000. A lock-mass infusion of [Glu^1]-fibrinopeptide B was used for internal calibration. To identify HOIP peptides, additional non-deuterated, uncomplexed samples were processed identically except that an MS^e fragmentation method was used. The output was processed using ProteinLynx Global Server v3.0.2 (Waters), using low- and high-energy thresholds of 250 and 100 respectively. Identified ion peaks were searched against the HOIP construct sequence used, using non-specific peptide cleavage and methionine oxidation as a variable modification. Results were analysed using DynamX v3.0 (Waters). PLGS-identified peptides were filtered to those of length 7-to 25 with a PLGS peptide score > 7.0. A low energy signal threshold of 500 was applied. Peptide and ion assignments were manually checked and refined where necessary.

Peptide-level deuteration data was exported from DynamX and further analysed in Spotfire v7.11 (TIBCO Software Inc., Palo Alto, CA, USA). Similarity clustering of this data across dAbs and peptides was performed using UPGMA (unweighted pair group method with arithmetic mean) clustering with a Euclidean distance measure, as implemented in Spotfire.

Protein Crystallization

A complex of HOIP RBR and dAb3 was prepared by mixing RBR:dAb3 at 1:3 molar ratio. The mixture was loaded onto a Superdex 75 10/300 GL column (GE Healthcare) with 50 mM HEPES pH 7.5, 150 mM NaCl and purified at a flow rate of 0.8 mL/min. Sample purity was evaluated by SDS-PAGE (Figures S4B and S4C). Fractions containing dAb3 and HOIP RBR were concentrated to 4-6 mg/mL with 5 kDa MWCO Vivaspin 20 and Vivaspin 500 centrifugal concentrators (Sartorius). The complex was crystallized in 1.22 M ammonium sulfate, 30-100 mM NaCl, 0.1 M HEPES pH 7.0 with 1:1 v/v protein:reservoir ratio at 20°C by vapor diffusion (Figure S4D). Apo dAb3 crystallized in 1 M ammonium dihydrogen phosphate, 0.1 M Tris pH 8.5. For this condition, crystallization trials were set up with dAb3/RBR complex at 8 mg/mL, but only dAb3 crystallized under this condition. Protein crystals were harvested with MicroLoops LD (MiTeGen), incubated with reservoir supplemented with 30% trehalose (for complex crystals) or cryo oil (Hampton Research) (for dAb3 crystals) as cryo-protectant and flash-frozen with liquid nitrogen.

Ligand Soaking

Inhibitor compounds were stored at 10-20 mM in 100% DMSO. Prior to soaking, compounds were diluted by 100-fold in crystallization reservoir solution to achieve final concentration of 1% DMSO. Trehalose was supplemented in the reservoir for a final concentration of 30% in the drop. Compound-soaked protein crystals were harvest after 24 h with MicroLoops LD and flash-frozen with liquid nitrogen.

Data Collection and Processing

dAb3, apo HOIP RBR/dAb3 complex crystals and crystals soaked with **3** or **4** were collected on MX beamline IO4 (wavelength 0.9795 Å); crystals soaked with **2** were collected on MX beamline IO4-1 (wavelength 0.9159 Å) while crystals soaked with **5** were collected on MX beamline IO3 (wavelength 0.9763 Å) at Diamond Light Source (UK) at 100K. Data were processed with autoPROC (Vonrhein et al., 2011) and DIALS (Clabbers et al., 2018; Lebedev et al., 2012; Winter et al., 2018). The dAb3 structure was solved by

molecular replacement using PHENIX (Adams et al., 2010) with a human VH antibody domain (PDB 1OHQ) (Jespers et al., 2004) as the search model. Apo dAb3/RBR complex structure was solved by molecular replacement using PHENIX with dAb3 molecules described above and the apo HOIP RING2 domain (PDB 4LJQ) (Stieglitz et al., 2013) as search models in addition to anomalous scattering. Fragment-bound RBR/dAb3 structures were solved by molecular replacement using PHENIX with the apo RBR/dAb3 structure as the search model. Iterative rounds of manual model building and refinement were performed with COOT (Emsley et al., 2010), REFMAC5 (Murshudov et al., 1997) and PHENIX. For the fragment-bound structures, fragment difference density consistent with the formation of a covalent bond to the sulphur of HOIP C885 can be clearly seen. Fragments were built and modified with JLigand (Lebedev et al., 2012). PHENIX was used to validate the final model. Structure figures were prepared in PyMOL (The PyMOL Molecular Graphics System, Version 2.3 Schrödinger, LLC.). To display electron density of compounds in PyMOL, 2Fo-Fc maps were created by fft (Read and Schierbeek, 1988; Ten Eyck, 1973) in CCP4 version 7.0.076 (Winn et al., 2011). Further details on data collection and refinement statistics are summarized in Table S1.

General Experimental Information for Compound Synthesis

Anhydrous solvents were commercially acquired in 100 mL bottles and used as received. Commercially acquired chemicals were used without further purification. Chemical names were generated in the ChemDraw Professional (v.17.0.0.206; PerkinElmer). Microwave reactions were carried out in sealed Biotage microwave vials in a Biotage Initiator+ microwave equipped with IR temperature control. For working up reaction mixtures, hydrophobic filters were generally used to remove traces of water from the extraction solvent.

Column chromatography was carried out on a Biotage Isolera One automated equipment. Biotage SNAP (Ultra) KP-Sil cartridges were used for normal phase. The samples were generally loaded dissolved in CH_2Cl_2 and eluted with mixtures of petroleum ether (bp 40-60), ethyl acetate and MeOH typically over 12 column volumes (CV).

UPLC-MS analysis was conducted on an Acquity UPLC CSH C18 column (50 mm × 2.1 mm, i.d. 1.7 μm packing diameter) at 40°C. Injection volume: 1 μL. The UV detection was a summed-up signal from wavelengths between 210 to 400 nm. UPLC retention times (*tr*) are reported in minutes. The solvents employed were A (0.1% formic acid in water, v/v) and B (0.1% formic acid in acetonitrile, v/v) in a gradient of 5→97% B in A (v/v) over 4 min. Flow rate 0.5 mL/min.

Preparative HPLC purification was conducted on an Agilent 5 C18 column (50 mm × 21.2 mm) at ambient temperature. The solvents employed for purification were A (0.1% formic acid in water, v/v) and B (0.1% formic acid in acetonitrile, v/v). Flow rate: 25 mL/min. Injection volume: <1 mL. The DAD UV detection was a summed-up signal from wavelengths between 210 to 360 nm, and the HPLC was coupled to an Agilent Infinitylab LC/MSD unit with positive/negative electrospray ionization (scan range 100 to 1000 AMU) and mass- and UV-based automated fraction collection.

^{1}H, ^{13}C and 2D NMR spectra were recorded on a Bruker 400 MHz instrument equipped with a 5 mm probe. The obtained FID-files were processed with MestReNova v.12.0.3-21384 software (Mestrelab Research). Signals are reported in ppm (δ) using the solvent as reference (DMSO-d_6: 2.50 ppm (^{1}H), 39.52 ppm (^{13}C); $CDCl_3$: 7.27 (^{1}H), 77.16 (^{13}C)) (Gottlieb et al., 1997). Coupling constants (*J*) are given in Hertz (Hz), and J_{HH} were rounded to the nearest 0.1 Hz. Multiplet patterns were assigned the following abbreviations or combinations of these: br – broad, m – multiplet, s – singlet, d – doublet, t – triplet. Signal assignment was made from unambiguous chemical shifts and COSY, HSQC and HMBC experiments. Unassigned aromatic signals were denoted Ar-C and Ar-CH respectively.

Low resolution mass spectrometry (LRMS) was recorded on a Waters ZQ MS unit with alternate scan positive/negative electrospray ionization (scan range 100 to 1000 AMU).

High resolution mass spectrometry (HRMS) was recorded on a Waters XEVO G2-XS MS unit with positive electrospray ionization (scan range 100 to 1200 AMU) after sample elution from an Acquity UPLC equipped with an CSH C18 column (100 mm × 2.1 mm, i.d. 1.7 μm packing diameter), held at 50°C, and using a solvent gradient of 97% A in B, v/v → 100% B over 8.5 min. The solvents employed were A (0.1% v/v formic acid in water, v/v) and B (0.1% formic acid in acetonitrile, v/v). Flow rate: 0.8 mL/min. Injection volume: 0.2 μL. Molecular masses were calculated in the ChemDraw Professional (v.17.0.0.206; PerkinElmer).

Thin layer chromatography (TLC) was carried out using pre-coated silica gel 60 plates that were eluted using mixtures of solvents as indicated. The plates were visualized using UV light (254 nm) or potassium permanganate stain, and retention factor (*Rf*) values were rounded to the nearest 0.05. The yields reported are from single reaction runs.

Compound Synthesis

The synthesis of compounds **2** and **3** have previously been reported (Johansson et al., 2019), while the synthesis of analogue **4** is described below. The synthesis of inhibitor **5** (HOIPIN-8) was carried out largely according to the procedure previously published by Katsuya et al. (Katsuya et al., 2019). Copies of NMR spectra and UPLC chromatograms for synthesized compounds are available in supplementary file Data S1.

2-(Methylamino)-2-oxoethyl (E)-4-(2-oxo-1,2,5,6,7,8-hexahydroquinoline-3-carboxamido)but-2-enoate (4)

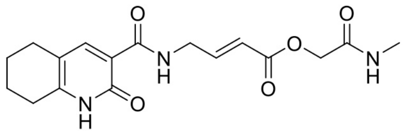

Activated ester formation: As previously reported (Johansson et al., 2019), (*E*)-4-(2-oxo-1,2,5,6,7,8-hexahydroquinoline-3-carboxamido)but-2-enoic acid **SI-1** (300 mg, 1.09 mmol) was stirred with 4-nitrophenyl 2-iodoacetate (367 mg, 1.2 mmol) and $NaHCO_3$ (142 mg, 1.7 mmol) in DMSO (3.5 mL) at ambient temperature for 24 h. The reaction mixture was filtered and adsorbed onto a Biotage SNAP cartridge, dried, and then purified by column chromatography (Biotage Ultra 25 g KP-Sil column) using a mixture of cyclohexane and EtOAc (50→100% EtOAc, v/v) to afford the activated ester **SI-2** (130 mg), which was used without further purification.

Amide formation: The activated ester **SI-2** (65 mg, 0.14 mmol) was stirred with *N,N*-diisopropylethylamine (49 μL, 0.28 mmol) and methylamine (72 μL of 2 M solution in THF, 0.14 mmol) in anhydrous DMF (1 mL) at ambient temperature under N_2 for 1 h. The reaction mixture was filtered and purified by preparative HPLC to afford the product amide **4** (10 mg, 5% over 2 steps) as a white solid. **UPLC**: t_r = 1.75 min; **^{1}H NMR** (400 MHz, DMSO-d_6): δ 12.28 (s, 1H; pyridone-NH), 10.07 (t, *J* = 5.9 Hz, 1H; N*H*CH_2), 8.07 (s, 1H; pyridone-H4), 7.99-7.93 (m, 1H; N*H*CH_3), 7.01 (dt, *J* = 15.7, 4.6 Hz, 1H; CH_2*CH*), 5.91 (dt, *J* = 15.7, 1.9 Hz, 1H; CHCOO), 4.48 (s, 2H; OCH_2), 4.16 (ddd, *J* = 6.3, 4.6, 2.0 Hz, 2H; *CH_2*CH), 2.62-2.56 (m, 5H; Ar-*CH_2*, NH*CH_3*), 1.68 (m, 4H; CH_2*CH_2CH_2*CH_2). One set of Ar-CH_2 signals overlaps with the solvent peak; **^{13}C NMR** (101 MHz, DMSO-d_6): δ 166.9 (CH_2*C*=O), 164.8 (ester-C=O), 163.7 ((*C*=O)NHCH_2), 162.0 (pyridone-C=O), 148.3 (pyridone-C6), 147.1 (CH_2*C*H), 145.3 (pyridone-C4), 119.4 (*C*HCOO), 116.9 (pyridone-C3), 113.9 (pyridone-C5), 62.4 (OCH_2), 26.3, 25.3 (*C*H_2CH$_2$CH$_2$*C*H$_2$), 25.2 (NHCH_3), 21.8, 20.9 (CH_2*C*H$_2$*C*H$_2$CH$_2$). The peak corresponding to *C*H_2CH overlaps with the solvent signal; **HRMS:** HRMS (ESI+) m/z [M + H]+ Calcd for $C_{17}H_{22}N_3O_5^+$ 348.1554, found 348.1569.

5-Bromo-3-hydroxyisobenzofuran-1(3H)-one (SI-3)

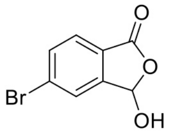

NBS bromination: Similar to the method of Katsuya et al. (Katsuya et al., 2019) 5-Bromoisobenzofuran-1(3H)-one (3.00 g, 14.1 mmol), *N*-bromosuccinimide (2.56 g, 14.4 mmol) and dibenzoyl peroxide (0.23 g, 0.7 mmol; 75 wt% hydrated solid) were dissolved in anhydrous $CHCl_3$ in a 100 mL round-bottom flask. The flask was fitted with a condenser and the reaction mixture was refluxed at 70°C for 4 h, then allowed to cool. CH_2Cl_2 (20 mL) was added and the combined organic fractions were washed with saturated aqueous $NaHCO_3$, water and brine (40 mL each). The organic solution was filtered through a hydrophobic frit and concentrated *in vacuo* to afford the crude product as a yellow oil that solidified upon standing. Purification by column chromatography (50 g SNAP Ultra column, 0→15% EtOAc in petroleum ether (40-60), v/v, over 12 column volumes) gave 3,5-dibromoisobenzofuran-1(3H)-one **SI-3-1** (2.94 g) as a white solid. **TLC**: R_f = 0.45 (30% EtOAc in petroleum ether, v/v); **^{1}H NMR** (400 MHz, $CDCl_3$): δ 7.83-7.74 (m, 3H; 3 × Ar-H), 7.35 (s, 1H; CHBr); **^{13}C NMR** (101 MHz, $CDCl_3$): δ 166.5 (C=O), 150.6 (Ar-C), 134.7 (Ar-CH), 130.6 (Ar-C), 127.3, 127.1 (2 × Ar-CH), 123.1 (Ar-C), 73.4 (CHBr).

Nucleophilic substitution: The intermediate 3,5-dibromoisobenzofuran-1(3H)-one **SI-3-1** (2.94 g, 10.1 mmol) was transferred to a 20 mL microwave vial and water (20 mL) was added. The vial was capped and the mixture was heated by microwave irradiation at 120°C for 3 h, then allowed to cool. The afforded solid was filtered off, washed with water (2 × 20 mL) and dried *in vacuo* to afford alcohol **SI-3** (2.20 g, 68% over 2 steps) as a white solid. **TLC**: R_f = 0.25 (30% EtOAc in petroleum ether, v/v); **UPLC**: t_r = 1.90 min; **^{1}H NMR** (400 MHz, DMSO-d_6): δ 8.28 (s, 1H; OH), 7.93 (s, 1H; Ar-H4), 7.86 (d, *J* = 8.3 Hz, 1H), 7.77 (d, *J* = 8.1 Hz, 1H) (Ar-H6, H7), 6.65 (s, 1H; OCH); **^{13}C NMR** (101 MHz, DMSO-d_6): δ 167.5 (C=O), 149.4 (Ar-C), 133.8 (Ar-CH), 128.5 (Ar-C), 127.0, 126.5 (2 × Ar-CH), 125.8 (Ar-C), 97.7 (OCH); **LRMS:** 229 $[M+H]^+$.

3-Hydroxy-5-(1-methyl-1H-pyrazol-4-yl)isobenzofuran-1(3H)-one (SI-4)

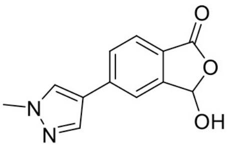

According to the method of Katsuya et al. (Katsuya et al., 2019) a 20 mL microwave vial was charged with bromide **SI-3** (0.50 g, 2.18 mmol), 1-methyl-4-(4,4,5,5-tetramethyl-1,3,2-dioxaborolan-2-yl)-1H-pyrazole (0.545 g, 2.62 mmol), Pd(dppf)Cl_2 (0.080 g, 5 mol%), and K_2CO_3 (0.905 g, 6.55 mmol). The vial was pump-filled with nitrogen and de-gassed 1,4-dioxane (18 mL) and H_2O (2 mL) was added. The mixture was heated in the microwave at 100°C for 2 h. The reaction mixture was quenched with 1 M aqueous HCl (13 mL) and filtered through a plug of Celite. The solids were washed with EtOAc (2 × 20 mL). The phases were separated, and the aqueous phase extracted with EtOAc (3 × 15 mL). The combined organic fractions were filtered through a hydrophobic frit and concentrated *in vacuo* to afford the crude product as a red solid. Purification by column chromatography (25 g SNAP Ultra column, 0→5% MeOH in EtOAc, v/v over 12 CV) afforded a solid that was washed with Et_2O (10 mL) and dried to give biaryl product **SI-4** (0.33 g, 65%) as a pink solid. **TLC**: R_f = 0.40 (5% MeOH in EtOAc, v/v); **UPLC**: t_r = 1.62 min; **^{1}H NMR** (400 MHz, DMSO-d_6): δ 8.39 (s, 1H; pyrazol-H), 8.16 (br s, 1H; OH), 8.08 (s, 1H; pyrazol-H), 7.87-7.83 (m, 2H), 7.81-7.76 (m, 1H) (3 × Ar-H), 6.65 (s, 1H; OCH), 3.88 (s, 3H; CH_3); **^{13}C NMR** (101 MHz, DMSO-d_6): δ 168.2 (C=O), 148.6 (Ar-C), 139.0 (Ar-C), 137.0, 129.3 (2 × pyrazol-CH), 126.9, 125.2 (2 × Ar-CH), 123.7, 120.7 (Ar-C), 119.2 (Ar-CH), 98.1 (OCH), 38.8 (CH_3); **LRMS:** 231 $[M+H]^+$.

Sodium (E)-2-(3-(2,6-difluoro-4-(1H-pyrazol-4-yl)phenyl)-3-oxoprop-1-en-1-yl)-4-(1-methyl-1H-pyrazol-4-yl) benzoate (5)

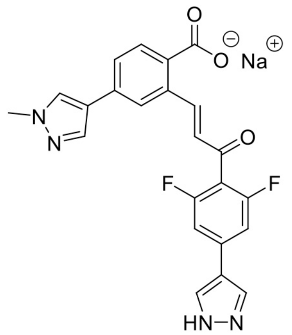

Alkylation: **SI-4** (0.29 g, 1.26 mmol) and 1-(4-bromo-2,6-difluorophenyl)ethan-1-one (0.30 g, 1.26 mmol) were dissolved in EtOH (15 mL) and 6 M aqueous NaOH was added (0.95 mL, 5.7 mmol). The mixture was stirred at ambient temperature for 30 min. The reaction was quenched with 1 M aqueous HCl (6.3 mL, 6.3 mmol) and the mixture was stirred for 10 min. Water (10 mL) was added and the EtOH was removed *in vacuo*. The residue was washed with cold water and EtOAc then dried to afford the product mixture of **SI-5a** and **SI-5b** (0.43 g) as a white solid.

Suzuki reaction: A 20 mL microwave vial was charged with **SI-5a** and **SI-5b** (0.43 g, 0.96 mmol), tert-butyl 4-(4,4,5,5-tetramethyl-1,3,2-dioxaborolan-2-yl)-1H-pyrazole-1-carboxylate (0.40 g, 1.35 mmol), Pd(dppf)Cl_2 (0.070 g, 10 mol%), and K_2CO_3 (0.40 g, 2.88 mmol), capped and pump-filled with nitrogen (3×). De-gassed THF (15 mL) and H_2O (1.5 mL) was added and the mixture was purged with nitrogen, then heated in the microwave at 90°C for 2 h. The reaction mixture was quenched on ice with 1 M HCl (7 mL), filtered through a plug of Celite and the solids were washed with EtOAc (3 × 10 mL). The phases were separated, and the aqueous phase was extracted with EtOAc (2 × 10 mL). The combined organic fractions were filtered through a hydrophobic frit and concentrated *in vacuo* to afford the crude biaryl product as a brown film, which was used in the next step without further purification.

Boc-deprotection: 4M HCl in 1,4-dioxane (15 mL, 60 mmol) was added and the crude mixture was stirred at ambient temperature under nitrogen 1.5 h. The solvent was removed *in vacuo* to give an orange solid. Purification by preparative HPLC gave **SI-6** (0.19 g) as a white solid.

Ring-opening: **SI-6** (0.155 g, 0.31 mmol) was dispersed in EtOH (10 mL) and H_2O (2 mL) and cooled to 0°C in an ice-water bath. 6 M aqueous NaOH (89 μL, 0.54 mmol) was then added, and the mixture was stirred at 0°C for 15 min and at ambient temperature for 45 min. The mixture was cooled to 0°C and ice-cold Et_2O (40 mL) was added. The mixture was stirred at 0°C for 1 h to give a precipitate, which was filtered off and washed with Et_2O (10 mL). Carboxylate **5** (0.133 g, 28% over 4 steps) sodium salt was isolated as a brown solid. **UPLC**: t_r = 2.20 min (C); **^{1}H NMR** (400 MHz, DMSO-d_6): δ 8.80 (d, J = 16.3 Hz, 1H; alkene-H), 8.25 (s, 1H), 8.18 (s, 2H), 7.96 (s, 1H) (4 × pyrazole-CH), 7.91 (d, J = 1.8 Hz, 1H), 7.62 (d, J = 8.0 Hz, 1H), 7.52 (dd, J = 8.0, 1.8 Hz, 1H), 7.45 (d, J = 9.4 Hz, 2H) (5 × Ar-H), 7.15 (d, J = 16.3 Hz, 1H; alkene-H), 3.86 (s, 3H; CH_3). The pyrazole-H was not detected. **^{13}C NMR** (101 MHz, DMSO-d_6): δ 187.9 (ketone-C=O), 170.0 (carboxylate-C=O), 160.9, 158.5 (2 × Ar-CF), 148.9 (alkene-CH), 142.4 (Ar-C), 136.2 (pyrazole-CH), 132.5 (br, 2 × pyrazole-CH), 132.3, 131.6 (2 × Ar-C), 130.2 (Ar-CH), 128.1 (pyrazole-CH), 126.5 (Alkene-CH), 126.3, 122.1 (2 × Ar-CH), 121.4, 118.7, 113.9 (3 × Ar-C), 107.9, 107.7 (2 × Ar-CH), 38.6 (CH_3). One Ar-C signal was not detected. **HRMS** (ESI+) m/z $[M+H]^+$ Calcd for $C_{23}H_{17}F_2N_4O_3^+$ 435.1263, found 435.1273.

CellPress

Protein LCMS Assay

HOIP RBR (2 μM) was incubated with compound **4** or **5** (20 μM), 1% v/v final DMSO concentration, in 50 mM HEPES pH 7.5, 150 mM NaCl buffer at 23°C. Final volume 75 μL. Aliquots (25 μL) were withdrawn after 0.5, 4 and 24 h incubation, flash frozen in liquid nitrogen and stored at -80°C. Each sample were individually defrosted immediately before injection into the LCMS. Acquisition of mass spectrometry data was carried out using positive electrospray ionisation (ESI) on an Agilent 6224 TOF LCMS system equipped with a Diode Array Detector (DAD), a Wellplate sampler (held at 10°C) and an Agilent PLRP-S reverse phase column (50 mm, 1 mm diameter, 5 μm particle size) held at 70°C. The following mobile phases were used for the LC: Mobile phase A (MP A) = 0.2% Formic acid in water, v/v; Mobile phase B (MP B) = 0.2% Formic acid in MeCN, v/v. The following LC method was employed: 0-0.5 min (10→30% B in A, v/v), 0.5-5 min (30→55% B in A, v/v), 5-5.7 min (55→60% B in A, v/v), 5.7-5.71 min (60→100% B in A, v/v), 5.71-6.2 min (100% B), 6.2-6.21 min (100→10% B in A, v/v), 6.21-8 min (10% B in A, v/v). Flow rate: 0.5 mL/min. Injection volume: 10 μL. The run length was 8 min, and equilibration time 1 min after each sample. DAD absorption was an average of wavelengths between 210 and 280 nm. The TOF mass spectrometer recorded 1 spectrum per second with a scan range between 700 and 2200 AMU. Proteins were deconvoluted in the Agilent MassHunter Workstation Software version B.06.00 build 6.0.633.10 using maximum entropy deconvolution with 1 Da mass step and baseline factor 7 subtractions.

QUANTIFICATION AND STATISTICAL ANALYSIS

Determination of Dissociation Constant (K_D) Values by Bio-Layer Interferometry (BLI)

Data were analysed using the Octet Data software (Pall FortéBio, version 9) and Microsoft Excel. The association rate constant (k_{on}) and error values were obtained by using the LINEST function in MS Excel, which uses the least squares method to calculate the statistics for a straight line. The error values of k_{off} were obtained from the standard deviation of independent analysis of the rate of dissociation from at least 4 different protein concentrations. The error values of the equilibrium dissociation constant (K_D) were obtained using the following equation in Excel:

$$\text{Error} = K_D * \sqrt{\left(\frac{k_{on}\ (\text{error})}{k_{on}\ (\text{value})}\right)^2 + \left(\frac{k_{off}\ (\text{error})}{k_{off}(\text{value})}\right)^2}$$

The values are reported in Table 1.

DATA AND CODE AVAILABILITY

Crystal structures generated in this study (PDB codes 6SC5, 6SC6, 6SC7, 6SC8, 6SC9, 6T2J) are available at RCSB Protein Data Bank (https://www.rcsb.org/).

Cell Chemical Biology, Volume *27*

Supplemental Information

Single-Domain Antibodies as Crystallization Chaperones to Enable Structure-Based Inhibitor Development for RBR E3 Ubiquitin Ligases

Yi-Chun Isabella Tsai, Henrik Johansson, David Dixon, Stephen Martin, Chun-wa Chung, Jane Clarkson, David House, and Katrin Rittinger

Supplemental Information

SI Item List

Figure S1

Figure S2

Figure S3

Figure S4

Figure S5

Figure S6

Table S1

Table S2

Table S3

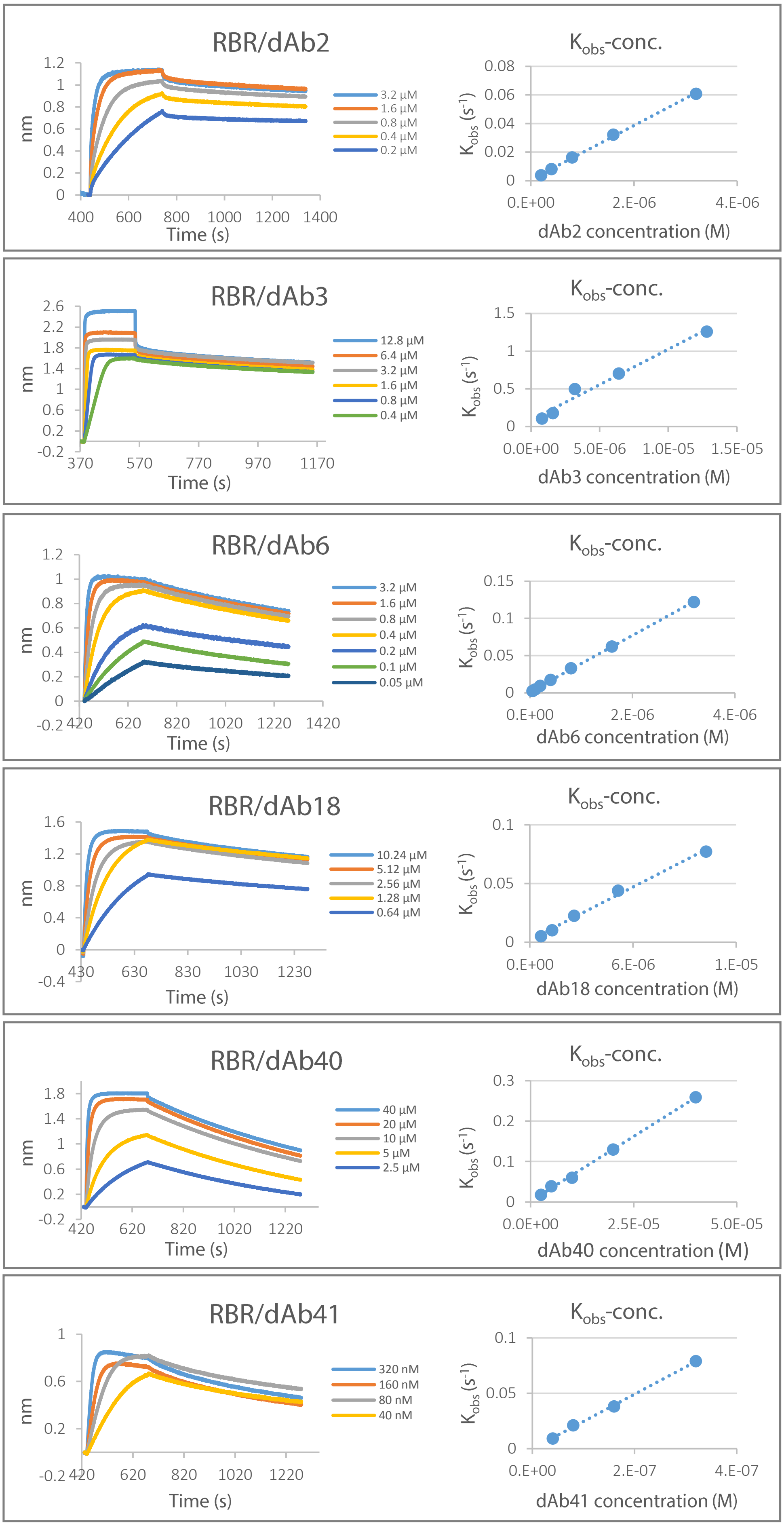

Figure S1. Related to Table 1. Kinetic analysis of the interaction between HOIP RBR and selected dAbs
Sensorgram traces displaying association and dissociation steps. Association rate constants (k_{on}) were obtained from the slope of the observed rate (k_{obs}) plotted versus dAb concentration.

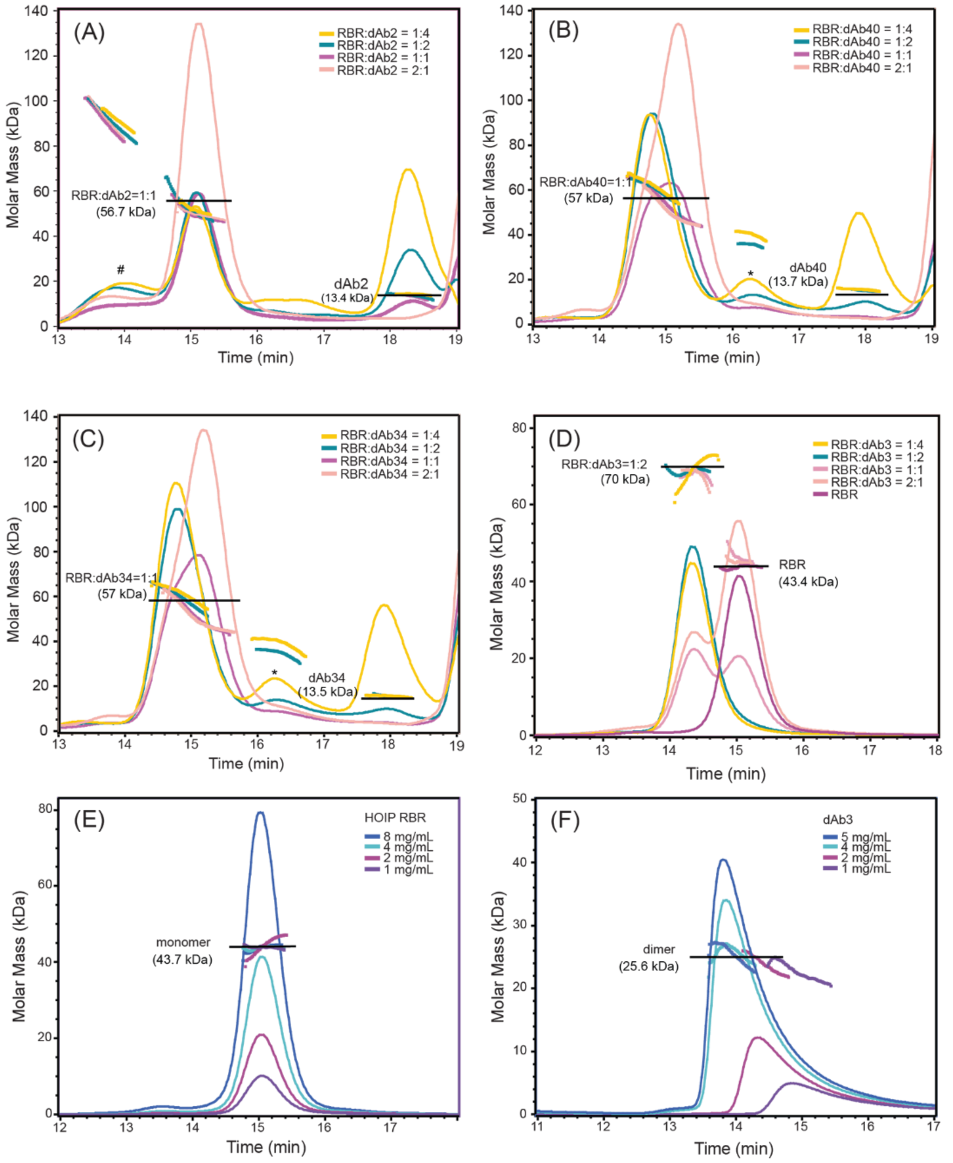

Figure S2. Related to Figure 1. SEC-MALLS analysis

(A) HOIP RBR and dAb2 (neutral dAb) were mixed at different ratios. All combinations demonstrated the same complex MW at 56.7 kDa, which is the MW of a 1:1 complex. # marks a HMW impurity present in the dAb2 preparation.
(B) HOIP RBR and dAb40 (neutral dAb) were mixed at different ratios. All combinations demonstrate the same complex MW at 57 kDa, which is the MW of a 1:1 complex. * marks a small fraction of self-associating dAb.
(C) HOIP RBR and dAb34 (differential dAb) were mixed at different ratios. All combinations demonstrate the same complex MW at 57 kDa, which is the MW of a 1:1 complex. * marks a small fraction of self-associating dAb.
(D) HOIP RBR and dAb3 were mixed at different ratios. HOIP RBR and dAb3 were saturated at a 1:2 ratio with an estimated complex MW at 70 kDa.
(E) HOIP RBR was analysed at 1-8 mg/mL. The estimated MW of HOIP RBR (43.7 kDa) is independent of the concentration indicating a monomeric behaviour in solution.
(F) dAb3 was analysed at 1-5 mg/mL. Protein at lower concentrations had longer retention times, which is likely due to sample-resin interaction under the buffer condition used. dAb3 is dimeric in solution.

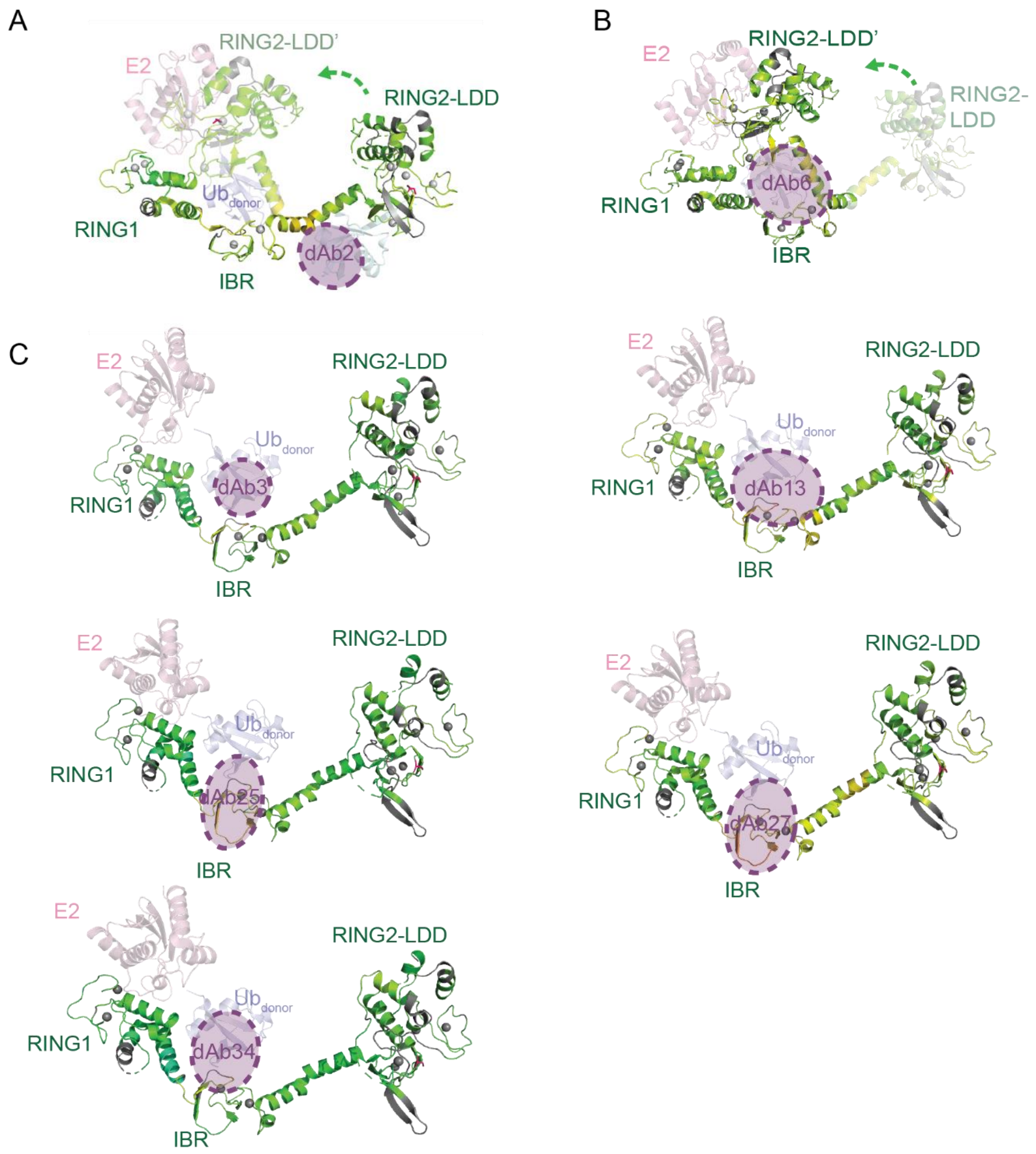

Figure S3. Related to Figure 2. Binding models of HOIP/dAb complexes

HDX-MS heatmaps displayed on the structure of HOIP RBR in complex with UbcH5B-Ub (PDB 5EDV). The elongated HOIP RBR molecule found in the crystal structure is indicated by RING2-LDD, whereas the closed form that is suggested to bind UbcH5B-Ub is indicated by RING2-LDD' (Lechtenberg et al., 2016). Structures are coloured according to the deuteration heatmap shown in Figure 2A, grey indicates regions for which no peptides were recovered.
(A) Model of possible binding mode of dAb2. For the neutral dAb2 it is suggested to bind to a site close to the linker between IBR and RING2-LDD.
(B) Model of possible binding mode of dAb6. Like dAb41 (Figure 2D) both inhibitory dAbs may bind close to active site C885 and possibly trap HOIP RBR in a closed conformation.
(C) Models of possible binding modes of dAb13, dAb25, dAb27 and dAb34, including dAb3 for comparison. These 5 differential dAbs are likely to bind close to the IBR and limit the flexibility of its adjacent linkers and thereby impact ubiquitin transfer.

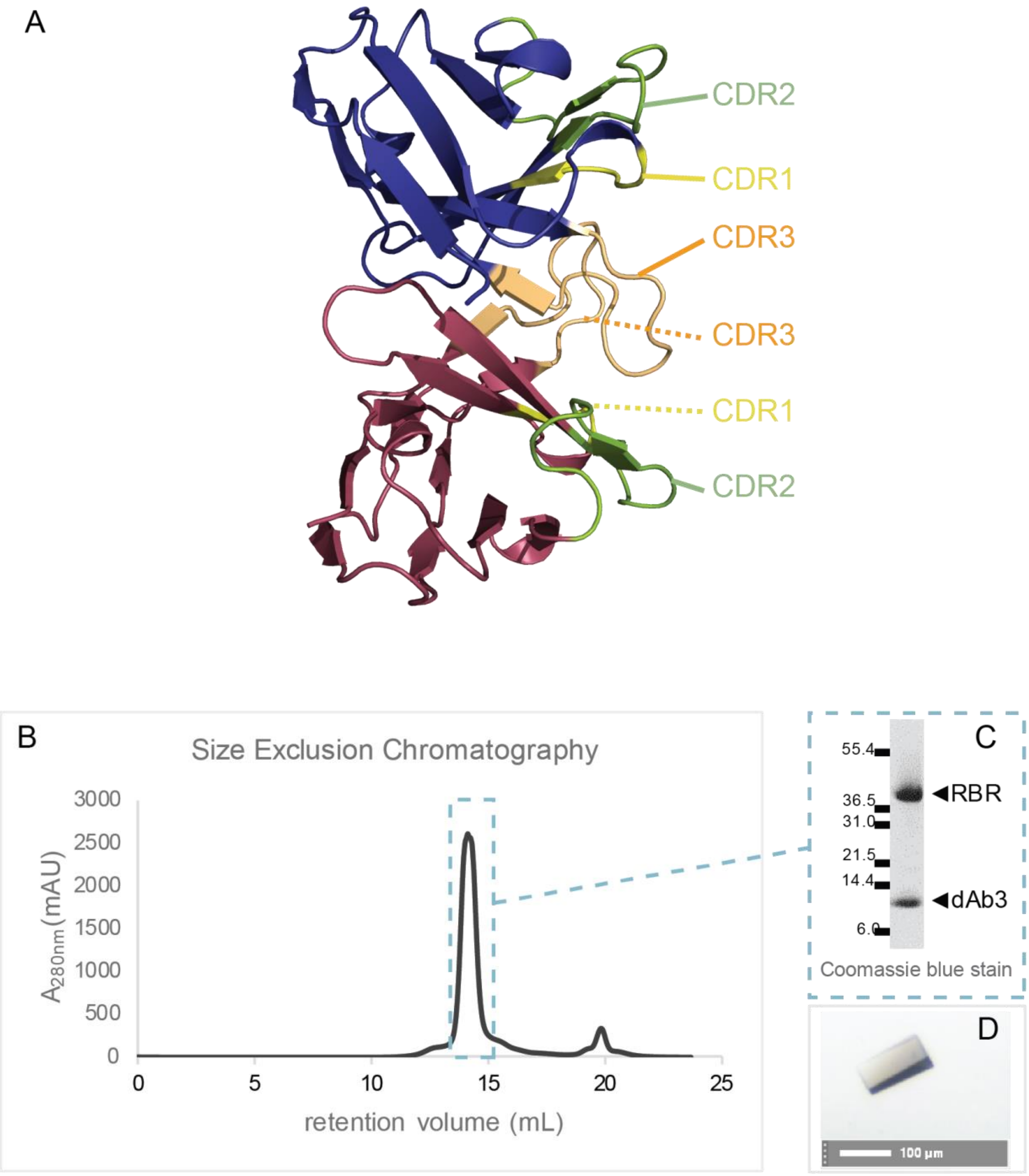

Figure S4. Related to Figure 3. Crystal structure of apo dAb3 and preparation of HOIP RBR/dAb3 complex for crystallization

(A) Colour scheme of the two dAb3 molecules (red and blue) is the same as presented in HOIP RBR/dAb3 structure in Figure 3A. CDR1 of the two dAbs is highlighted in yellow, CDR2 in green and CDR3 in orange.
(B) The HOIP RBR/dAb3 complex eluted in a single peak in size exclusion chromatography.
(C) The composition and purity of the complex examined by SDS-PAGE. The gel has been stained with Coomassie Blue and converted to grey scale.
(D) Crystal produced under the condition of 1.22 M ammonium sulfate, 30-100 mM NaCl, 0.1 M HEPES pH 7.0. Scale bar of 100 µm is shown.

A

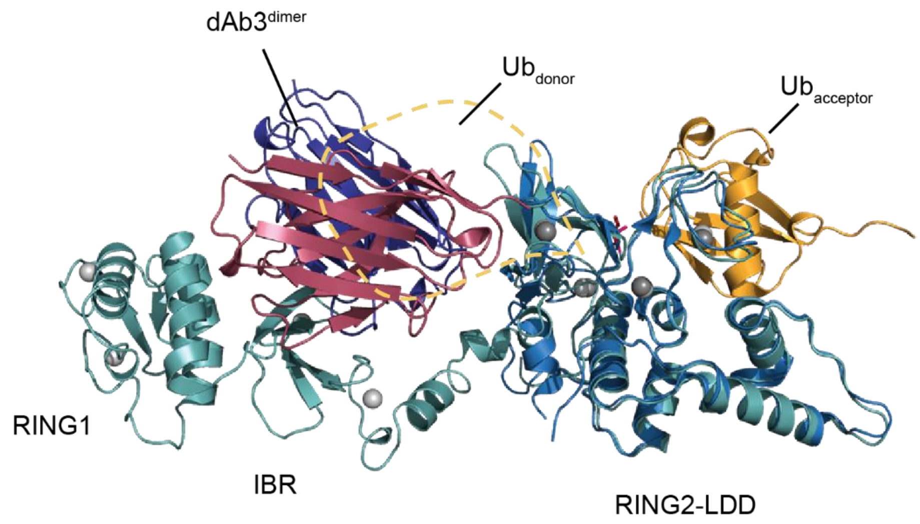

B

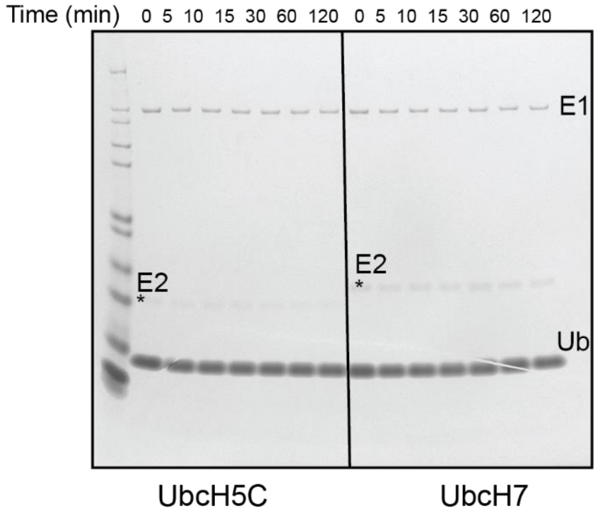

Figure S5. Related to Figures 1 and 4. Structural comparison of dAb-bound and ubiquitin-bound HOIP and control ubiquitination assays without E3 ligase

(A) Structural comparison of the HOIP RBR domain (in teal) in complex with dAb3 (in red and blue) and ubiquitin-bound RING2-LDD (4LJO, in light blue), overlapped via the RING2-LDD domain. The acceptor ubiquitin is shown in orange and the positon of the donor ubiquitin is indicated by yellow-dotted line.

(B) Control ubiquitination assays without E3 ligase to test for possible background activities of UbcH5C and UbcH7. Assays were carried out under the same conditions as those presented in Figure 1. The assays show that there is no background activity without the RBR domain of HOIP.

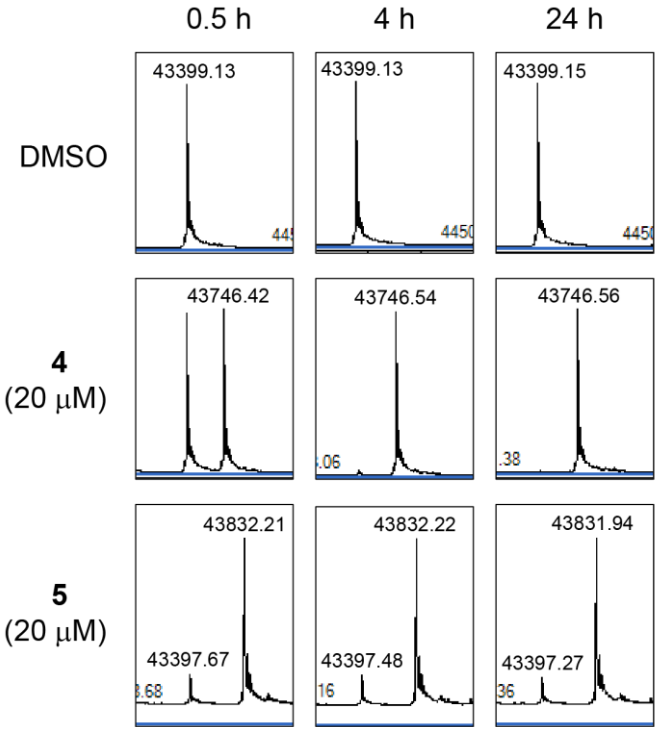

Figure S6. Related to Figure 5. Analysis of labelling of HOIP RBR (2 μM) by covalent compounds 4 and 5 (20 μM) after 0.5, 4 and 24 h incubation.

Table S1. Related to STAR Methods section of data collection and processing. Data collection and refinement statistics

	dAb3/HOIP-RBR (apo)	dAb3/HOIP-RBR - compound (**2**)	dAb3/HOIP-RBR - compound (**3**)	dAb3/HOIP-RBR - compound (**4**)	dAb3/HOIP-RBR - HOIPIN-8	dAb3 (apo)
PDB	6SC6	6SC5	6SC7	6SC8	6SC9	6T2J
**Data collection**						
Space group	I 2 2 2	I 2 2 2	I 2 2 2	I 2 2 2	I 2 2 2	C 1 2 1
Cell dimensions						
a, *b*, *c* (Å)	65.95, 87.49, 241.93	65.58, 86.66, 240.22	65.71, 86.70, 240.55	65.92, 87.77, 241.83	66.13, 87.05, 241.54	85.74, 59.31, 63.85
α, β, γ (°)	90, 90, 90	90, 90, 90	90, 90, 90	90, 90, 90	90, 90, 90	90, 131.97, 90
R_{merge}	2.86 (26.09)*	2.48 (26.38)	3.66 (50.46)	2.94 (77.70)	4.89 (70.14)	7.44 (38.36)
$I / \sigma I$	21.22 (4.97)	13.75 (2.29)	9.93 (1.33)	13.86 (0.77)	5.57 (1.02)	11.36 (2.78)
Completeness (%)	98.95 (96.62)	99.93 (99.90)	99.89 (99.60)	99.35 (95.48)	99.81 (98.51)	99.78 (99.35)
Multiplicity	2.0 (2.0)	2.0 (2.0)	2.0 (2.0)	2.0 (2.0)	2.0 (2.0)	4.8 (4.8)
**Refinement**						
Resolution (Å)	60.48 - 2.25 (2.33 - 2.25)	63.26 - 2.1 (2.18 - 2.1)	60.14 - 2.56 (2.65 - 2.56)	48.32 - 2.11 (2.18 - 2.11)	81.9 - 2.47 (2.56 - 2.47)	43.42 - 1.70 (1.76 - 1.70)
No. reflections	33399 (3205)	40481 (3997)	22647 (2218)	40843 (3847)	25596 (2510)	26230 (2586)
R_{work}	19.93 (29.17)	21.92 (29.57)	22.38 (31.74)	21.87 (38.34)	20.98 (31.78)	17.51 (23.64)
R_{free}	24.62 (34.88)	25.29 (34.91)	26.71 (36.43)	26.08 (40.96)	25.25 (39.55)	20.81 (27.95)
No. atoms						
Macromolecules	4724	4631	4620	4647	4597	1828
Ligands	35	50	91	50	52	5
Solvent	169	105	74	151	68	179
B-factors						
Macromolecules	52.28	56.87	66.14	66.05	67.36	17.0
Ligand/ion	75.47	66.30	73.60	76.85	70.14	40.4
Water	48.24	51.97	59.28	64.80	63.49	28.3
R.m.s. deviations						
Bond lengths (Å)	0.003	0.002	0.002	0.003	0.002	0.009
Bond angles (°)	0.61	0.46	0.47	0.57	0.50	1.04

*Values in parentheses are for highest-resolution shell.

Table S2. Related to STAR Methods section of oligonucleotides in key resources table.

Oligonucleotides		
HOIP-(697-1072)-AviTag FW primer	Sigma Aldrich Oligos	5’CAGGGACCCGGTCAGGAGTGTGC CGTGTGTGGC
HOIP-(697-1072)-AviTag RV primer 1	Sigma Aldrich Oligos	5’CTTCAAAGATGTCGTTCAGACCCT TCCGCCTGCGGGGGATACTC
HOIP-(697-1072)-AviTag RV primer 2	Sigma Aldrich Oligos	5’TTCGTGCCATTCGATTTTCTGCGC TTCAAAGATGTCGTTCAGACC
HOIP-(697-1072)-AviTag RV primer 3	Sigma Aldrich Oligos	5’GGCACCAGAGCGTTATTCGTGCCA TTCGATTTTCTGCG
HOIP-(697-859)-AviTag FW primer	Sigma Aldrich Oligos	5’CCTCTTTCAGGGACCCGGTCA GGAGTGTGCCGTGTGTGGC
HOIP-(697-859)-AviTag RV primer 1	Sigma Aldrich Oligos	5’CTTCAAAGATGTCGTTCAGACC GCCCTGGGCCTGGTATTCTGGG
HOIP-(697-859)-AviTag RV primer 2	Sigma Aldrich Oligos	5’TTCGTGCCATTCGATTTTCTGC GCTTCAAAGATGTCGTTCAGACC
HOIP-(697-859)-AviTag RV primer 3	Sigma Aldrich Oligos	5’CCGCGTGGCACCAGAGCGTTA TTCGTGCCATTCGATTTTCTGCG
HOIP-(748-859)-AviTag FW primer	Sigma Aldrich Oligos	5’CCTCTTTCAGGGACCCGGTGGCC GCCCCGACCTCACCG
HOIP-(748-859)-AviTag RV primer 1	Sigma Aldrich Oligos	5’CTTCAAAGATGTCGTTCAGACCGC CCTGGGCCTGGTATTCTGGG
HOIP-(748-859)-AviTag RV primer 2	Sigma Aldrich Oligos	5’TTCGTGCCATTCGATTTTCTGCGC TTCAAAGATGTCGTTCAGACC
HOIP-(748-859)-AviTag RV primer 3	Sigma Aldrich Oligos	5’CCGCGTGGCACCAGAGCGTTATTC GTGCCATTCGATTTTCTGCG
HOIP-(853-1072)-AviTag FW primer	Sigma Aldrich Oligos	5’CCTCTTTCAGGGACCCGGTCCAGA ATACCAGGCCCAGGGCC
HOIP-(853-1072)-AviTag RV primer 1	Sigma Aldrich Oligos	5’CTTCAAAGATGTCGTTCAGACCCT TCCGCCTGCGGGGGATACTC
HOIP-(853-1072)-AviTag RV primer 2	Sigma Aldrich Oligos	5’TTCGTGCCATTCGATTTTCTGCGC TTCAAAGATGTCGTTCAGACC
HOIP-(853-1072)-AviTag RV primer 3	Sigma Aldrich Oligos	5’CCGCGTGGCACCAGAGCGTTATTC GTGCCATTCGATTTTCTGCG
DOM006	Sigma Aldrich Oligos	5’ATGGTTGTTGTCATTGTCGGCGCA
DOM008	Sigma Aldrich Oligos	5’AGCGGATAACAATTTCACACAGGA
DOM009	Sigma Aldrich Oligos	5’CGCCAGGGTTTTCCCAGTCACGAC
DOM57	Sigma Aldrich Oligos	5’ATGAGGTTTTGCTAAACAACTTTC
V_H clones FW primer	Sigma Aldrich Oligos	5’CCGTCTCGAGCTAATAAGAATTCA CTGGCCGTCGT
V_H clones RV primer	Sigma Aldrich Oligos	5’GAATTCTTATTAGCTCGAGACGGT GACCAGGGTTCC
Vκ clones FW primer	Sigma Aldrich Oligos	5’GGAAATCGAACGGTAATAAGAATT CACTGGCCGTCGT
Vκ clones RV primer	Sigma Aldrich Oligos	5’GAATTCTTATTACCGTTCGATTTCC ACCTTGGTCCC

Table S3. Related to STAR Methods section of soluble dAb construction. CDR sequences of select dAbs defined by Kabat numbering scheme

Clone ID	Clone Name		CDR1	CDR2	CDR3
CL-208735	49D077-26F11-1	dAb 3	GYSMA	TISPIGTYTYYADSVKG	GSYSRGTPF---------DY
CL-208673	49D077-26H02-1	dAb 2	FYSME	SIDAIGGRTYYADSVKG	VLPVSAENRF--------DY
CL-209011	49D077-30B01-1	dAb 6	DVDMG	AISEKGSGTYYADSVKG	SPSFGHF-----------DY
CL-209398	49D077-34E05-1	dAb 13	PAVMG	GIQEYGSKTYYADSVKG	DPYPRALGVF--------DV
CL-210069	49D077-42D01-1	dAb 18	RGWMY	SIPALGPDTYYADSVKG	RGSHGF------------DY
CL-210519	49D077-47F02-1	dAb 25	QPSML	SISGDGFHTYYADSVKG	TPTYAVEVLPVGEY----DV
CL-210525	49D077-47D03-1	dAb 27	GYSMI	SITEDGSATYYADSVKG	TPTYAVEVLPVGEY----DV
CL-209486	49D077-35E05-1	dAb 34	GRIMG	TISDEGPRTYYADSVKG	PVVLGYEGGPTPDF----DV
CL-210427	49D077-46B02-1	dAb 40	KAKMG	TIGHTGVPTYYADSVKG	PLPTSRPTGF--------DY
CL-209805	49D077-39D01-1	dAb 41	EYPMR	TISYDGSHTYYADSVKG	VILAQSSPLTRF------DY
